# CtBP1/2 restrict DUX-dependent and independent 2C-like programs in mouse embryonic stem cells

**DOI:** 10.1038/s44319-026-00881-7

**Published:** 2026-08-03

**Authors:** Kazuma Yoshioka, Maki Ichisakino, Kota Sugiyama, Nao Hayakawa, Selma Alamanda Abadi, Heekyoung Yoon, Miyu Marutani, Ryo Masuda, Kyo Takahashi, Yoshiyuki Seki

**Affiliations:** Department of Biomedical Sciences, School of Biological and Environmental Sciences, Kwansei Gakuin University, Hyogo, Japan

**Keywords:** Chromatin, Transcription & Genomics, Signal Transduction, Stem Cells & Regenerative Medicine

## Abstract

After fertilization, maternally deposited mRNAs are cleared, and de novo transcription is initiated through zygotic genome activation (ZGA), a core event of the maternal-to-zygotic transition in mice. 2-cell-like cells (2CLCs), a rare MERVL-positive subpopulation of mouse embryonic stem cells, partially recapitulate transcriptional features of 2-cell embryos. Although canonical MERVL-high 2CLCs depend on DUX, *Dux* knockout embryos can develop to term, suggesting that 2CLC models do not fully capture DUX-independent pathways associated with preimplantation transcriptional programs. Here, we show that disruption of C-terminal binding protein 1/2 (Ctbp1/2) activates both DUX-dependent minor ZGA-associated genes and DUX-independent major ZGA- and post-ZGA-associated programs. *Pramel7* is derepressed independently of DUX and contributes to subsets of both programs. PRAMEL7 overexpression partially rescues transcriptional defects caused by *Dux* deletion and is associated with UHRF1 downregulation and DNA demethylation-linked activation of post-ZGA-associated genes. These findings identify CtBP1/2 as repressors of multiple early embryonic transcriptional programs in mouse embryonic stem cells.

## Synopsis

CtBP1/2 loss activates both DUX-dependent minor-ZGA and DUX-independent major/post-ZGA transcriptional programs in mouse ESCs. PRAMEL7 links these pathways by promoting Dux expression and UHRF1 downregulation-associated DNA demethylation.

- *Ctbp1/2* deletion expands MERVL-EGFP-positive ESCs and reveals distinct DUX-dependent high-GFP and DUX-independent low-GFP populations.
- CtBP1/2 directly represses rapid-response major-ZGA genes, including *Pramel7* and *Gata1*.
- DUX controls delayed minor-ZGA genes.
- *Ctbp1/2* depletion upregulates PRAMEL7, which activates post-ZGA-associated genes through UHRF1 downregulation.

## Abstract (web summary)

CtBP1/2 loss activates both DUX-dependent minor-ZGA and DUX-independent major/post-ZGA transcriptional programs in mouse ESCs. PRAMEL7 links these pathways by promoting Dux expression and UHRF1 downregulation-associated DNA demethylation.

## Graphical abstract

**Figure:**
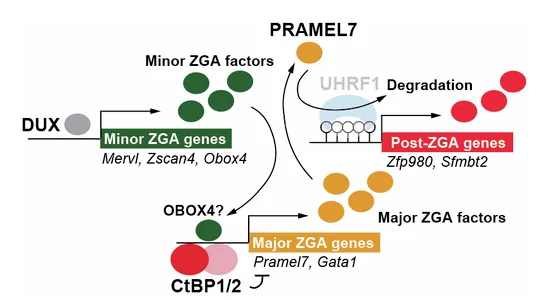

**Figure:**
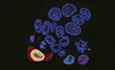

## Introduction

After fertilization, maternal factors direct development and trigger zygotic genome activation (ZGA) around the late one-cell stage in mice, as part of the maternal-to-zygotic transition (MZT) (Eckersley-maslin et al, 2018). Early cleavage-stage embryos acquire cellular totipotency to generate both embryonic proper and extraembryonic tissues during MZT. Although totipotency was thought to be exclusive to the early-cleavage-stage embryos, part of this molecular program is recapitulated in the totipotent-like cells (2-cell-like cells; 2CLCs) marked by the expression of endogenous retrovirus MERVL, which are an endogenous subpopulation of mouse embryonic stem cells (ESCs) (Macfarlan et al, 2012; Rodriguez-Terrones et al, 2018). Recent work further demonstrated that MERVL transcription is not merely a marker of the two-cell state, but is functionally required for preimplantation embryo development and contributes to the regulation of two-cell-stage gene expression and the transition from the totipotent to pluripotent state in early mouse embryos (Sakashita et al, 2023).

The transition from pluripotent stem cells into 2CLCs is a useful system for identifying the gene regulatory network for ZGA, and several genes that control the emergence of 2CLCs from pluripotent cells have been identified (Eckersley-Maslin et al, 2019; Hu et al, 2020; Ishiuchi et al, 2015; Percharde et al, 2018; Rodriguez-Terrones et al, 2018). The transcription factor DUX (DUX4 in humans) is a critical factor for regulating the transition from pluripotent stem cells into 2CLCs and the expression of ZGA genes (Whiddon et al, 2017; Hendrickson et al, 2017; De Iaco et al, 2017). Ectopic expression of DUX enhances the population of 2CLCs, whereas *Dux* disruption eliminates the emergence of spontaneous 2CLCs during mESC culture. Several 2CLC regulatory genes have been identified, but they are either direct activators (DPPA2/4, p53, and NELFA) or direct repressors (CAF-1, LINE-1, and SETDB1) of *Dux* expression (Ishiuchi et al, 2015; Percharde et al, 2018; Hu et al, 2020; Eckersley-Maslin et al, 2019; Grow et al, 2021). However, both zygotic and maternal *Dux* knockout embryos can develop to term (Guo et al, 2019; Chen and Zhang, 2019), suggesting that conventional DUX-dependent 2CLC models may not fully capture DUX-independent regulatory pathways associated with early embryonic transcriptional programs.

CtBP1 and CtBP2 are transcriptional corepressors involved in multiple developmental and cellular processes (Hildebrand and Soriano, 2002; Stankiewicz et al, 2014). We previously showed that CtBP1/2 cooperate with PRC2 to regulate transcriptional repression during the transition from primed to naive pluripotency (Yamamoto et al, 2020). In this study, we show that disruption of *Ctbp1/2* activates two separable transcriptional programs in mESCs: a DUX-dependent program enriched for minor ZGA-associated genes and a DUX-independent program enriched for major ZGA- and post-ZGA-associated genes. We further show that *Pramel7* is derepressed by *Ctbp1/2* loss even in the absence of DUX and contributes to a subset of DUX-dependent and DUX-independent transcriptional programs, partly through UHRF1 downregulation and DNA demethylation-dependent regulation. These findings reveal that CtBP1/2 restricts multiple early embryonic transcriptional programs in mESCs through both DUX-dependent and DUX-independent mechanisms.

## Results

### CtBP1/2 act as negative regulators of the transition from pluripotent ESCs to 2-cell-like cells (2CLCs)

**Figure 1:**
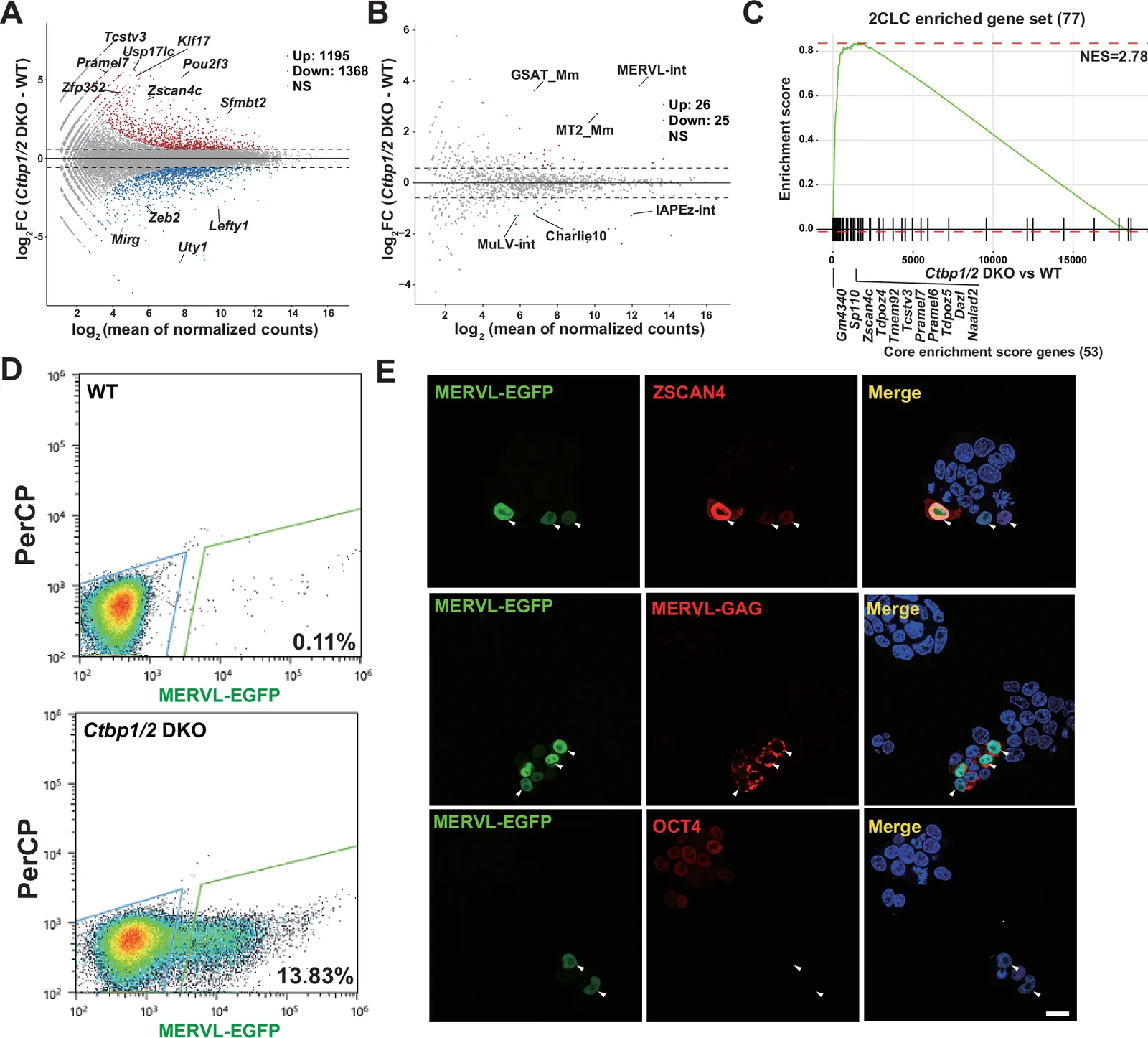
Disruption of *Ctbp1/2* expands the MERVL-EGFP-positive 2-cell-like cell population. (**A**, **B**) MA plots showing the relationship between mean normalized counts and log2 fold change between *Ctbp1/2* DKO and WT ESCs for genes (**A**) and repetitive elements (**B**). RNA-seq was performed using *n* = 2 biological replicates. Differential expression was assessed using DESeq2 based on a Wald test, and *P* values were adjusted for multiple testing using the Benjamini–Hochberg method. Differentially expressed features were defined as adjusted *P* ≤ 0.05 and absolute fold change ≥2. Red and blue dots indicate significantly upregulated and downregulated features, respectively; gray dots indicate non-significant features. (**C**) Gene set enrichment analysis (GSEA) showing enrichment of a 2 CLC-enriched gene set among genes upregulated in *Ctbp1/2* DKO ESCs relative to WT ESCs. (**D**) Representative flow cytometry plots of MERVL-EGFP reporter activity in WT and *Ctbp1/2* DKO ESCs. (**E**) Immunofluorescence analysis showing that MERVL-EGFP-positive cells co-express ZSCAN4 and MERVL-GAG proteins, and are mutually exclusive with OCT4 expression. Nuclei were counterstained with DAPI. Scale bar: 10 μm. Source data are available online for this figure.

We have previously shown that CtBP1/2 are essential for the transition from formative to naive pluripotency in mouse embryonic stem cells (mESCs) (Yamamoto et al, 2020). To investigate their roles beyond pluripotency maintenance, we performed RNA sequencing (RNA-seq) to compare the gene expression profiles between wild-type (WT) and *Ctbp1/2* double-knockout (DKO) ESCs. MA-plots revealed a marked upregulation of two-cell (2C)-specific genes, including *Dux*, *Zscan4*, and *Zfp352*, together with repeat elements such as MERVL, MT2, and GSAT_Mm in *Ctbp1/2* DKO ESCs (Fig. 1A,B). Gene set enrichment analysis (GSEA) using a reference set of 2C-like cell (2CLC)-enriched genes (*n* = 77 (Macfarlan et al, 2012)) confirmed a strong enrichment of 2CLC-related transcripts among those upregulated in the absence of CtBP1/2 (Fig. 1C).

To directly monitor the induction of the 2CLC state, we generated *Ctbp1/2* DKO ESCs carrying the MERVL-EGFP reporter (Ishiuchi et al, 2015). The EGFP-positive fraction was ~0.1% in WT ESCs, whereas *Ctbp1/2* DKO ESCs exhibited more than a 100-fold increase in EGFP-positive cells (13.83%), indicative of robust MERVL-EGFP reporter activation (Fig. 1D). These EGFP-positive cells co-expressed hallmark 2CLC markers such as ZSCAN4 and MERVL-GAG, while pluripotency-associated factors such as OCT4 were markedly reduced (Fig. 1E), consistent with previous reports (Macfarlan et al, 2012).

### Loss of *Ctbp1/2* drives both DUX-independent and DUX-dependent MERVL-EGFP activation

**Figure 2:**
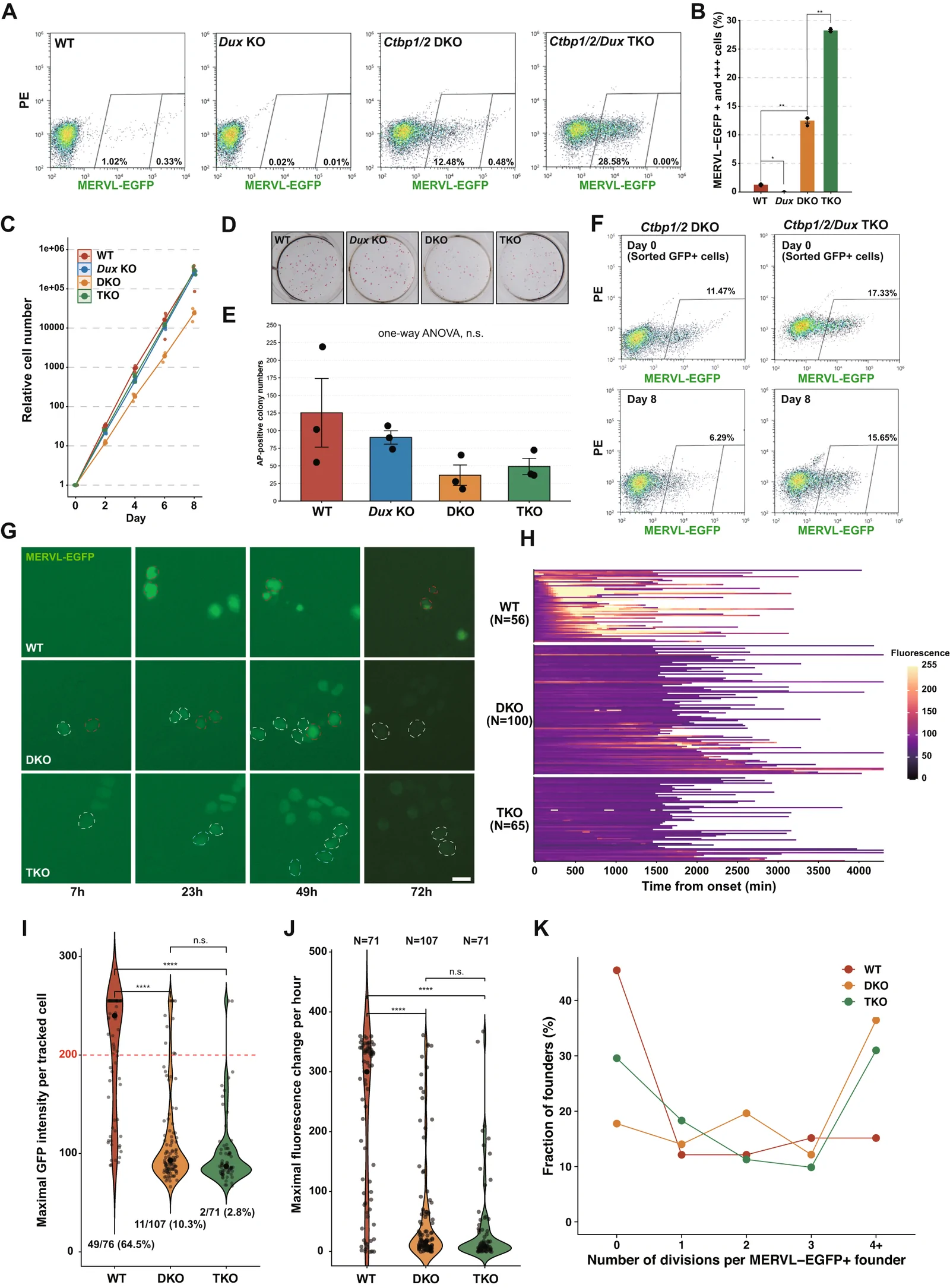
Loss of *Ctbp1/2* activates both DUX-independent and DUX-dependent programs, leading to MERVL-EGFP activation. (**A**) Flow cytometry analysis (FACS) of MERVL::TurboGFP reporter expression in wild-type (WT), *Dux* knockout (KO), *Ctbp1/2* double knockout (DKO), and *Ctbp1/2*/*Dux* triple knockout (TKO) ESCs. (**B**) Percentage of MERVL-EGFP-positive cells obtained by FACS. Bars indicate mean ± SEM from three biological replicates; individual data points are shown. Statistical significance was assessed by a two-sided Welch’s *t* test followed by a Bonferroni correction for multiple comparisons. *P < 0.05, **P < 0.01, ***P < 0.001, ****P < 0.0001; n.s., not significant. Exact adjusted *P* values for the annotated comparisons are: WT versus *Dux* KO, *P* = 0.0273; WT versus *Ctbp1/2* DKO, *P* = 0.00640; *Ctbp1/2* DKO versus *Ctbp1/2/Dux* TKO, *P* = 0.00117. Complete pairwise adjusted *P* values are provided in Source Data. (**C**) Growth curves of WT, *Dux* KO, *Ctbp1/2* DKO, and *Ctbp1/2/Dux* TKO ESCs. Relative cell number is shown over the indicated culture periods. Lines indicate mean ± SEM from three biological replicates. Statistical significance was assessed between cell types at each time point using two-sided Welch’s *t* tests on log10-transformed relative cell numbers, followed by Bonferroni correction for pairwise comparisons within each time point. Day 0 was not tested because all samples were normalized to the baseline relative cell number of 1. Statistical annotations are not shown in the panel to avoid overloading the growth curve; complete same-day pairwise adjusted *P* values, including n.s. comparisons, are provided in Source Data. (**D**) Representative images of alkaline phosphatase (AP)-positive colonies derived from WT, *Dux* KO, DKO, and TKO ESCs. (**E**) Quantification of AP-positive colony numbers is shown in (**D**). Bars represent mean ± SEM, and dots indicate individual biological replicates. Statistical significance was assessed by one-way ANOVA followed by Tukey’s HSD multiple-comparison test. No significant differences were detected among the groups. Exact adjusted *P* values for all pairwise comparisons are provided in Source Data. (**F**) Representative flow cytometry plots of sorted MERVL-EGFP-positive cells from DKO and TKO ESCs immediately before sorting (day 0) and after 8 days of re-culture. Numbers indicate the percentage of GFP-positive cells. (**G**) Representative time-lapse images of MERVL-EGFP signals in WT, DKO, and TKO ESCs at the indicated time points. Red dashed circles indicate collapsed cells. White dashed circles indicate dividing cells. Light blue dashed circles indicate cells reverted to GFP-negative cells. Scale bar: 10 μm. (**H**) Heatmaps showing GFP fluorescence intensity traces of individual tracked cells from WT, DKO, and TKO ESCs, aligned to the onset of GFP expression. The number of tracked cells is indicated for each genotype. (**I**) Violin plots showing the maximal GFP intensity of each tracked cell in WT, DKO, and TKO ESCs. The red dashed line indicates the threshold of 200 fluorescence units. Numbers below indicate the number and percentage of cells exceeding this threshold. Statistical significance was assessed by two-sided Wilcoxon rank-sum tests followed by Bonferroni correction for multiple comparisons. *P < 0.05, ****P < 0.0001; n.s., not significant. Exact adjusted *P* values for the annotated comparisons are: WT versus DKO, *P* = 1.65 × 10^−17^; WT versus TKO, *P* = 1.09 × 10^−18^; DKO versus TKO, *P* = 0.0775. Complete pairwise adjusted *P* values are provided in Source Data. (**J**) Violin plots showing the maximal fluorescence change per hour of each tracked cell in WT, DKO, and TKO ESCs. The number of analyzed cells is indicated above each group. Statistical significance was assessed by two-sided Wilcoxon rank-sum tests followed by Bonferroni correction for multiple comparisons. ****P < 0.0001; n.s., not significant. Exact adjusted *P* values for the annotated comparisons are: WT versus DKO, *P* = 3.92 × 10^−11^; WT versus TKO, *P* = 1.10 ×  10^−11^; DKO versus TKO, *P* = 0.0745. Complete pairwise adjusted *P* values are provided in Source Data. (**K**) Fraction of MERVL-EGFP-positive founder lineages classified by the number of cell divisions during tracking. Only selected comparisons are shown in the figure. Complete pairwise adjusted *P* values are provided in Source Data. Source data are available online for this figure.

DUX, a homeobox transcription factor, is both essential and sufficient for the emergence of spontaneous 2CLCs from ESCs (Whiddon et al, 2017; Hendrickson et al, 2017; De Iaco et al, 2017). To determine whether the 2C-like program triggered by *Ctbp1/2* deficiency depends on DUX, we deleted the *Dux* cluster in both WT and *Ctbp1/2* DKO ESCs (Appendix Fig. S1C). We first compared the population of MERVL-EGFP-positive cells among WT, *Dux* KO, *Ctbp1/2* DKO, and *Ctbp1/2*/*Dux* triple-KO (TKO) ESCs. Based on fluorescence intensity, the MERVL-EGFP-positive population was subdivided into GFP+ and GFP + ++ fractions. Interestingly, although the total percentage of the MERVL-EGFP-positive population in *Ctbp1/2* DKO ESCs was significantly expanded compared with that in WT ESCs, the percentage of GFP + ++ cells was comparable between WT and DKO ESCs. The high GFP + + + /GFP+ ratio in WT cells suggests that the GFP+ state is largely a transient intermediate that efficiently progresses to the GFP + ++ state. In contrast, DKO cells accumulated in the GFP+ fraction with only limited progression to the GFP + ++ fraction. In TKO ESCs, the GFP + ++ fraction was largely lost, whereas the lower-intensity GFP+ fraction remained detectable and was expanded relative to DKO ESCs (Fig. 2A,B). We next examined the growth and self-renewal properties of *Ctbp1/2* DKO and *Ctbp1/2/Dux* TKO ESCs. Loss of *Ctbp1/2* impaired cell growth, and this defect was rescued by *Dux* deletion, suggesting that activation of the DUX-dependent 2CLC program contributes to the reduced growth rate of *Ctbp1/2*-deficient ESCs (Fig. 2C). Alkaline phosphatase staining showed that *Ctbp1/2* DKO ESCs formed fewer AP-positive colonies than WT ESCs, and this reduction was not restored by additional *Dux* deletion (Fig. 2D,E). To monitor whether MERVL-EGFP-positive cells revert to a GFP-negative state, we sorted GFP-positive cells from *Ctbp1/2* DKO or *Ctbp1/2/Dux* TKO ESCs and re-cultured them for 8 days. In both genotypes, a GFP-negative fraction reappeared during re-culture (Fig. 2F).

We next performed time-lapse recording to monitor the kinetics of MERVL-EGFP signals and cell division/death. In WT ESCs, MERVL-EGFP signals were rapidly induced upon cell division, and many strongly GFP-positive cells subsequently collapsed, consistent with a previous report (Olbrich et al, 2021) (Fig. 2G,H; Movie EV1). In contrast, in *Ctbp1/2* DKO ESCs, rapidly emerging, strongly GFP-positive 2CLCs were rarely observed. Instead, the majority of cells remained weakly GFP-positive and continued to divide (Movie EV2). A smaller fraction ( ~ 10%) of cells gradually increased GFP intensity to an intermediate or high level, after which they either continued dividing, collapsed, or reverted to a GFP-negative state (Movie EV3). In TKO ESCs, only MERVL-EGFP-weak-positive cells emerged, followed by cell division and reversion to GFP-negative cells (Movie EV4). To compare the maximal GFP intensity, maximal fluorescence change, and number of divisions among WT, DKO, and TKO, we tracked the fluorescence intensity and division history of each lineage (Fig. 2I–K). Most tracked WT cells (64.5%) reached GFP intensities greater than 200, whereas DKO cells were separated into two populations; one showing high GFP intensity (10.3%) and the other remaining at low GFP intensity. In contrast, most TKO cells remained in the low-intensity population. A similar trend was observed for maximal fluorescence change, with WT cells showing large increases, DKO cells consisting of both high- and low-change populations, and TKO cells remaining mostly in the low-change population (Fig. 2J). Finally, to compare the number of divisions after GFP onset among WT, DKO, and TKO cells, we tracked cell divisions in each lineage (Fig. 2K). Approximately half of WT cells failed to undergo further division after becoming GFP-positive, and lineages with more than four divisions were rare. In contrast, lineages undergoing more than three divisions became the predominant population in DKO and TKO cells.

Taken together, these findings indicate that loss of *Ctbp1/2* activates both DUX-dependent and partially DUX-independent routes leading to MERVL-EGFP activation. Although a DUX-dependent route to stronger GFP activation is retained, its mode of emergence is markedly altered in *Ctbp1/2*-deficient ESCs: instead of the abrupt appearance and frequent collapse seen in WT ESCs, GFP-positive cells more often persist in an intermediate state, continue dividing, or revert to the GFP-negative state. By contrast, the DUX-independent route follows a distinct trajectory characterized by a weak GFP-positive state, continued cell division, and reversibility, and is observed in both DKO and TKO ESCs.

### Loss of CtBP1/2 derepresses DUX-independent gene modules associated with the early/intermediate 2CLC transition and major ZGA-related programs

**Figure 3:**
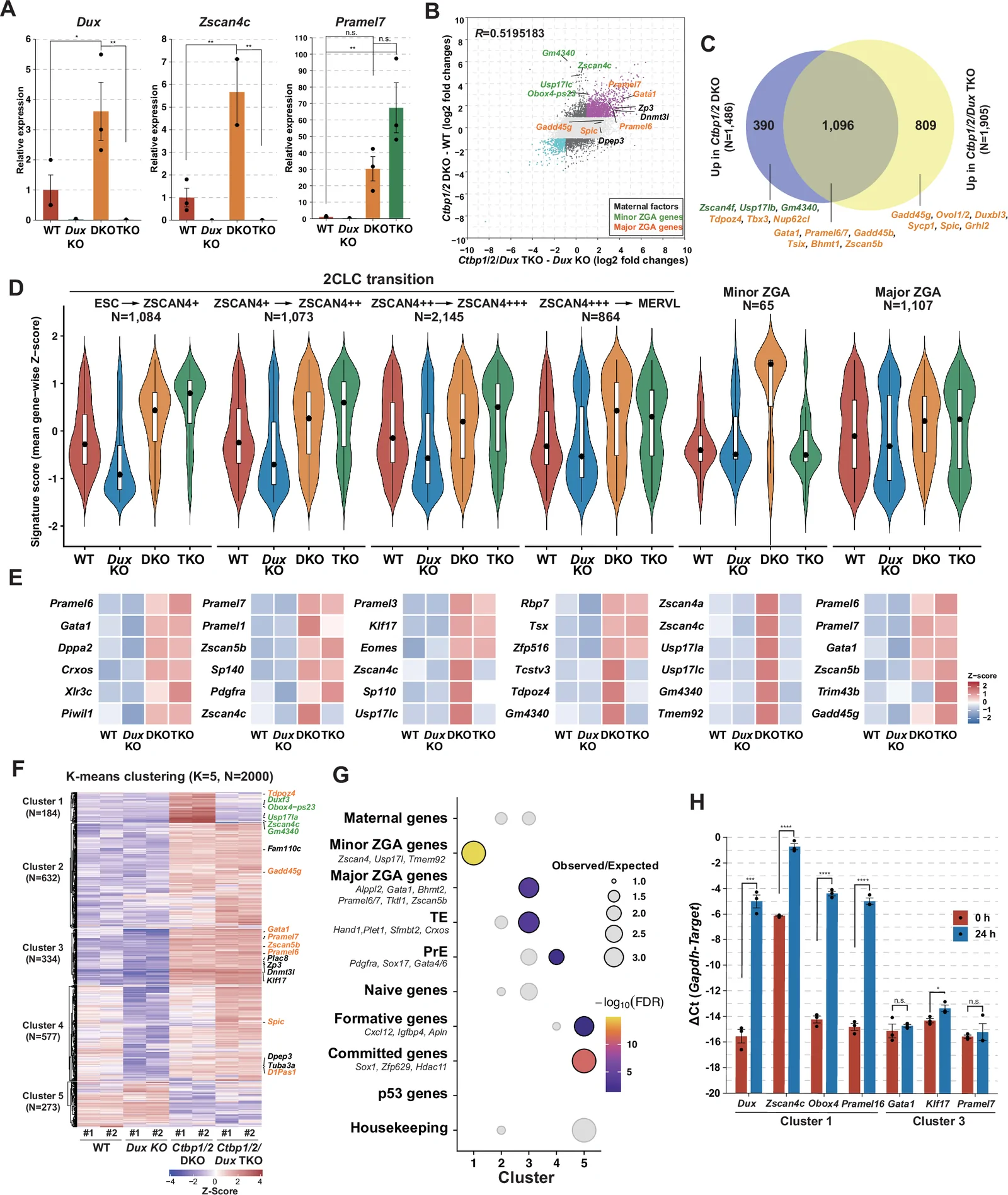
Loss of *Ctbp1/2* derepresses DUX-independent gene modules associated with the early/intermediate 2CLC transition and major ZGA-related programs. (**A**) RT-qPCR analysis of 2C-associated genes in WT, *Dux* KO, *Ctbp1/2* DKO, and *Ctbp1/2/Dux* TKO ESCs. Values are shown as relative expression with WT set to 1. Bars indicate mean ± SEM from *n* = 3 biological replicates; individual data points are shown. Statistical significance was assessed separately for each gene by one-way ANOVA followed by Tukey’s HSD multiple-comparison test. *P < 0.05, **P < 0.01; n.s., not significant. Exact adjusted *P* values for the annotated comparisons are: *Dux*, WT versus *Ctbp1/2* DKO, *P* = 0.0378; *Dux*, *Ctbp1/2* DKO versus *Ctbp1/2/Dux* TKO, *P* = 0.00678; *Zscan4c*, WT versus *Ctbp1/2* DKO, *P* = 0.00570; *Zscan4c*, *Ctbp1/2* DKO versus *Ctbp1/2/Dux* TKO, *P* = 0.00227; *Pramel7*, WT versus *Ctbp1/2/Dux* TKO, *P* = 0.00242; *Pramel7*, *Ctbp1/2* DKO versus *Ctbp1/2/Dux* TKO, *P* = 0.0580. Complete pairwise adjusted *P* values are provided in Source Data. (**B**) Scatter plot comparing gene expression changes induced by *Ctbp1/2* DKO in WT (*Y* axis) and *Dux* KO (*X* axis) backgrounds. (**C**) Venn diagram showing the overlap of genes upregulated in *Ctbp1/2* DKO and TKO. (**D**) Violin plot showing the signature scores of each gene selected from the upregulated genes during transition from ESCs to ZSCAN4 + , ZSCAN4+ to ZSCAN4 + + , ZSCAN4 + + to ZSCAN4 + + + , ZSCAN4 + ++ to MERVL+ in WT, *Dux* KO, *Ctbp1/2* DKO, and *Ctbp1/2*/*Dux* TKO ESCs. For each gene, replicate expression values were averaged within each genotype, and gene-wise z-scores were calculated across the four genotypes. Violin plots show the distribution of gene-wise z-scores. In the embedded box plots, the center line indicates the median, the lower and upper bounds of the box indicate the 25th and 75th percentiles, respectively, and the lower and upper whisker ends indicate the smallest and largest values within 1.5× the interquartile range. Outliers outside this range are not shown. Black dots indicate the median. (**E**) Heatmap showing representative genes of the violin plot. (**F**) K-means clustering analysis (K = 5) of the top 2,000 most variable genes across WT, *Dux* KO, *Ctbp1/2* DKO, and *Ctbp1/2/Dux* TKO ESCs. (**G**) Enrichment analysis of selected developmental gene sets across the five clusters shown in (**F**). Dot size indicates the observed/expected ratio, and color indicates −log_10_(FDR). Representative genes included in each gene set are shown on the left. (**H**) RT-qPCR after *Dux* induction (0 h vs 24 h) for selected genes (Cluster 1: *Dux, Zscan4c, Obox4, Pramel16,* and* Cluster 3: Gata1, Klf17, Pramel7*). The ΔCt of each gene was calculated as Ct(*Gapdh*) − Ct(*target gene*). Means of ΔCt were calculated from *n* = 3 biological replicates. Error bars indicate SEM. Statistical significance was assessed separately for each gene by one-way ANOVA followed by Tukey’s HSD multiple-comparison test. *P < 0.05, **P < 0.01, ***P < 0.001, ****P < 0.0001; n.s., not significant. Exact adjusted *P* values for 0 h versus 24 h are: *Zscan4c*, *P* = 2.50 × 10^−5^; *Obox4*, *P* = 7.31 × 10^−6^; *Pramel16*, *P* = 1.24 × 10^−5^; *Gata1*, *P* = 0.510; *Klf17*, *P* = 0.0383; *Pramel7*, *P* = 0.621. Complete adjusted *P* values are provided in the Source Data. Source data are available online for this figure.

Re-analysis of the published embryo RNA-seq dataset from a previous study (Guo et al, 2019) showed that *Pramel7* was upregulated at the 2-cell stage in both WT and *Dux* KO embryos (Appendix Fig. S2A). RT-qPCR analysis further confirmed that *Pramel7* remained elevated in *Ctbp1/2* DKO and was further increased in *Ctbp1/2/Dux* TKO ESCs, indicating that derepression of *Pramel7* upon *Ctbp1/2* loss occurs independently of DUX (Fig. 3A). In contrast, *Zscan4c* upregulation upon *Ctbp1/2* depletion was abolished by *Dux* deletion, indicating strict DUX dependency. To assess transcriptional activity of MERVL long terminal repeat (MERVL-LTR) sequences, we performed a luciferase reporter assay (Appendix Fig. S2B). *Ctbp1/2* loss led to robust activation of the MERVL-LTR reporter, and mutation of the DUX-binding motif (DUXmut-MERVL-LTR) markedly reduced, but did not abolish, this activity. Reporter activity in *Ctbp1/2/Dux* TKO ESCs remained substantially higher than *Dux* KO, indicating that CtBP1/2 also represses MERVL through DUX-independent mechanisms.

Published DUX ChIP-seq data (Hendrickson et al, 2017) revealed DUX occupancy near both the *Zscan4c* and *Naalad2* loci, with strong binding signals overlapping MERVL-LTR or MT2 family elements (Appendix Fig. S2C). At the *Zscan4c* locus, DUX bound both the proximal regulatory region, with a strong peak, and distal MERVL elements. At the *Naalad2* locus, DUX-binding sites overlapped with MERVL sequences. Reporter assays confirmed that MERVL-independent elements proximal to the *Zscan4c* locus were activated by *Ctbp1/2* loss in a DUX-dependent manner, whereas MERVL-LTR-containing elements derived from the *Zscan4c* and *Naalad2* loci retained partial activity even in *Ctbp1/2/Dux* TKO ESCs (Appendix Fig. S2D,E). These luciferase assay results were consistent with our RNA-seq data (Appendix Fig. S2F).

We next performed RNA-seq to identify genes induced by *Ctbp1/2* loss in a DUX-dependent or DUX-independent manner. A scatter plot comparing gene expression changes between WT and *Ctbp1/2* DKO ESCs (*Y* axis) and between *Ctbp1/2/Dux* TKO and *Dux* KO (*X* axis) ESCs revealed a moderate correlation (*R* = 0.52), suggesting that a substantial fraction of CtBP1/2-regulated genes are induced independently of DUX (Fig. 3B). A Venn diagram showed that 1096 of 1486 genes upregulated in DKO ESCs (73.8%) were also upregulated in TKO ESCs (Fig. 3C; Dataset EV1). To further examine whether genes derepressed upon *Ctbp1/2* loss are preferentially associated with CtBP2-bound regions, we used a publicly available CtBP2 ChIP-seq dataset (Kim et al, 2015) and assigned CtBP2 ChIP-seq peaks to nearby genes using GREAT with the “Two nearest genes” rule. DKO-upregulated genes were significantly enriched for CtBP2-associated genes compared with all genes detected in our RNA-seq dataset (451/1481, 30.5%, versus 4589/20,380, 22.5%) (Appendix Fig. S2G). DKO-downregulated genes also showed enrichment for CtBP2-associated genes (237/820, 28.9% versus 4589/20,380, 22.5%), indicating that CtBP2-associated loci are broadly represented among genes whose expression is altered upon *Ctbp1/2* loss. To address the possibility that 2CLC expansion upon loss of *Ctbp1/2* reflected a stressed cellular state, we assessed the expression of a curated p53 target gene set and found no significant differences among WT, *Dux* KO, DKO, and TKO ESCs (Appendix Fig. S2H). Consistently, the canonical p53 target genes *Cdkn1a* and *Mdm2* were unchanged (Appendix Fig. S2I). Together, these data argue against a p53-mediated stress response as the primary explanation for MERVL-EGFP activation following *Ctbp1/2* loss (Grow et al, 2021).

To further define the identity of this residual GFP+ population, we examined gene signatures associated with sequential stages of 2CLC transition, including the transitions from ESCs to ZSCAN4-low cells, from ZSCAN4-low to ZSCAN4-mid cells, from ZSCAN4-mid to ZSCAN4-high cells, and from ZSCAN4-high cells to MERVL-EGFP-positive cells, based on the trajectory reported for the transition from ESC to 2CLC (Rodriguez-Terrones et al, 2018) (Fig. 3D,E). Genes associated with early entry into the 2CLC program, particularly those linked to the ESC-to-ZSCAN4-low transition, were reduced in *Dux* KO ESCs, but remained upregulated in *Ctbp1/2* DKO ESCs even in the absence of DUX and tended to be further elevated in *Ctbp1/2/Dux* TKO ESCs. By contrast, the fully activated MERVL-high/minor-ZGA state was impaired in TKO ESCs. These findings suggest that loss of *Ctbp1/2* promotes entry into an early/intermediate 2CLC transition program independently of DUX, whereas DUX is required for progression to the canonical MERVL-high 2CLC state. Accordingly, *Dux* deletion in the *Ctbp1/2* DKO background abolishes the GFP + ++ population and prevents progression to the canonical 2CLC state, while allowing cells to accumulate in an early/intermediate stage of 2CLC transition. We next examined gene signatures of minor and major ZGA genes. Induction of the minor ZGA program by *Ctbp1/2* loss was abolished by *Dux* KO, whereas relative upregulation of the major ZGA program was retained in *Ctbp1/2/Dux* TKO ESCs.

Because previous studies have shown that OBOX family members can activate ZGA genes in ESCs (Ji et al, 2023; Sakamoto et al, 2024; Guo et al, 2024), we next examined the expression of *Obox* genes in WT, *Dux* KO, DKO, and TKO ESCs. *Obox4* was elevated upon loss of *Ctbp1/2*, but this increase was attenuated, although not completely abolished, by *Dux* deletion (Appendix Fig. S2J). In contrast, *Pramel7* expression was further upregulated by *Dux* KO in *Ctbp1/2* DKO ESCs, suggesting that its regulation in this context is not fully accounted for by OBOX induction alone.

To classify these regulatory patterns, we applied K-means clustering analysis (*K* = 5) using the 2000 most variable genes across WT, *Dux* KO, *Ctbp1/2* DKO, and *Ctbp1/2/Dux* TKO ESCs (Fig. 3F; Appendix Fig. S2K). Cluster 1 genes, including *Zscan4* and *Usp17l* family genes, were slightly repressed in *Dux* KO ESCs but strongly upregulated in *Ctbp1/2* DKO ESCs, and this upregulation was abolished by *Dux* KO. Cluster 2 genes, including *Gadd45g*, were upregulated in both *Ctbp1/2* DKO and *Ctbp1/2/Dux* TKO ESCs, representing DUX-independent targets. Cluster 3 genes, including *Gata1*, *Pramel7*, and *Zscan5b*, showed moderate downregulation in *Dux* KO ESCs and were modestly upregulated in both *Ctbp1/2* DKO and *Ctbp1/2/Dux* TKO ESCs. Cluster 4 genes, including *Spic*, were downregulated by *Dux* KO in WT ESCs. However, this downregulation by *Dux* KO was not observed in *Ctbp1/2* DKO ESCs, indicating that the loss of CtBP1/2 abolishes the dependency of these genes on DUX. Cluster 5 genes were consistently downregulated in both *Ctbp1/2* DKO and *Ctbp1/2/Dux* TKO ESCs, indicating positive regulation by CtBP1/2 in a DUX-independent manner.

To evaluate the expression dynamics of the upregulated clusters (Cluster 1–4) in early mouse embryos, we compared these clusters with public RNA-seq data from early embryos (Zhang et al, 2016), excluding genes with an FPKM average ≤1 (Appendix Fig. S2L). Cluster 1 genes were transiently activated at the 2–4-cell stage, typical of minor ZGA genes, whereas Cluster 2–4 included maternal genes that were downregulated after fertilization and zygotic genes activated after the late 2-cell stage. To further examine whether the transcriptional changes induced by loss of *Ctbp1/2* were selective rather than non-specific, we performed enrichment analysis of selected gene sets across the individual clusters (Fig. 3G; Appendix Fig. S2M). Consistent with our previous analyses, minor ZGA genes were significantly enriched in Cluster 1. In contrast, the other *Ctbp1/2* loss-induced clusters showed distinct developmental associations: Cluster 3 was enriched for major ZGA genes and trophectoderm-associated genes, including *Cdx2*, *Eomes*, and *Krt18*, whereas Cluster 4 was enriched for primitive endoderm-associated genes, including *Gata6*, *Sox17*, and *Pdgfra*. In addition, Cluster 5 was enriched for formative and committed gene sets. These results suggest that loss of *Ctbp1/2* does not cause indiscriminate derepression, but rather activates multiple discrete developmental gene modules.

Finally, to examine the dependency of genes upregulated by *Ctbp1/2* DKO on DUX, we measured the expression changes of genes belonging to Cluster 1 and Cluster 3 upon inducible *Dux* expression using doxycycline-inducible *Dux* ESCs (Fig. 3H). Consistent with our RNA-seq data, Cluster 1 genes were consistently upregulated, whereas Cluster 3 genes showed little or no change following short-term DUX induction. Taken together, these results demonstrate that *Ctbp1/2* depletion upregulates DUX-dependent minor ZGA genes and DUX-independent major ZGA genes and post-ZGA genes, revealing a dual regulatory system that links CtBP1/2-mediated repression to early embryonic transcriptional programs.

### Temporal regulation of ZGA-associated genes by CtBP1/2 defines rapid and delayed response genes

**Figure 4:**
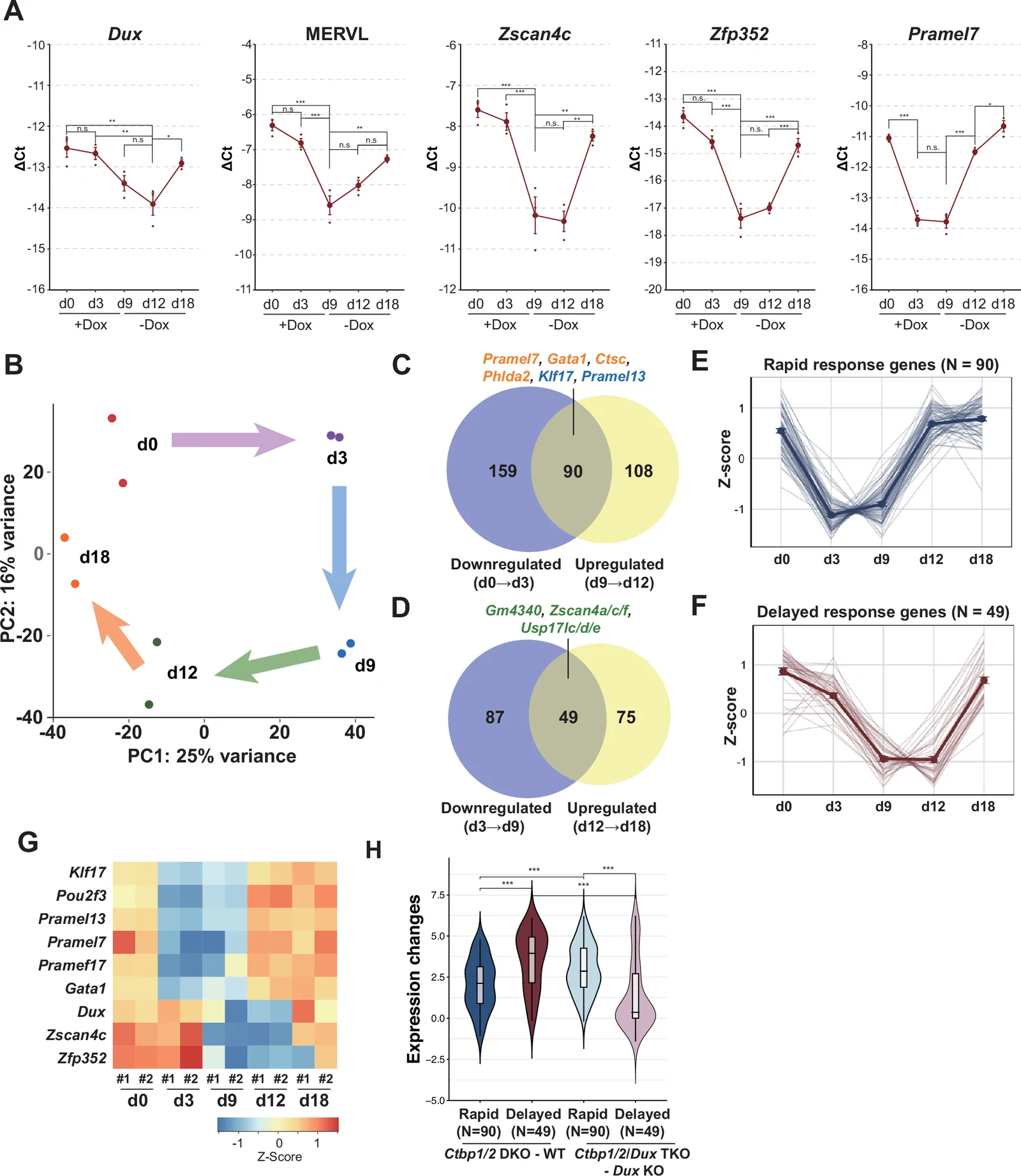
Temporal regulation of ZGA genes by CtBP1/2 defines rapid and delayed responses. (**A**) RT-qPCR analysis of *Dux*, MERVL*, Zscan4c*, *Zfp352*, and *Pramel7* in *Ctbp1/2* DKO ESCs with or without doxycycline (Dox). The ΔCt was calculated as Ct (*Gapdh*) – Ct (target gene). Means were calculated from *n *= 3 biological replicates, and error bars indicate SEM. Statistical significance was assessed separately for each gene by one-way ANOVA followed by Tukey’s HSD multiple-comparison test. *P < 0.05, **P < 0.01, ***P < 0.001; n.s., not significant. Exact adjusted *P* values for the annotated comparisons are: *Dux*, d0 versus d3, *P* = 0.988; d0 versus d12, *P* = 0.00376; d3 versus d12, *P* = 0.00751; d9 versus d12, *P* = 0.396; d12 versus d18, *P* = 0.0280. MERVL, d0 versus d3, *P* = 0.277; d0 versus d9, *P* = 1.6 × 10^−5^; d3 versus d9, *P* = 1.4 × 10^−4^; d9 versus d12, *P* = 0.186; d9 versus d18, *P* = 0.00159; d12 versus d18, *P* = 0.0571. *Zscan4c*, d0 versus d9, *P* = 3.48 × 10^−4^; d3 versus d9, *P* = 9.00 × 10^−4^; d9 versus d12, *P* = 0.994; d9 versus d18, *P* = 0.00328; d12 versus d18, *P* = 0.00191. *Zfp352*, d0 versus d3, *P* = 0.128; d0 versus d9, *P* = 6.0  × 10^−6^; d3 versus d9, *P* = 7.5 × 10^−5^; d9 versus d12, *P* = 0.796; d9 versus d18, *P* = 1.12 × 10^−4^; d12 versus d18, *P* = 4.10 ×  10^−4^. *Pramel7*, d0 versus d3, *P* = 1.0 × 10^−6^; d3 versus d9, *P* = 0.996; d9 versus d12, *P* = 4.27 × 10^−6^; d12 versus d18, *P* = 0.0139. Complete pairwise adjusted *P* values are provided in Source Data. (**B**) Principal component analysis (PCA) of the CtBP2 induction/depletion time course in *Ctbp1/2* DKO ESCs with or without Dox. (**C**) Venn diagram showing the overlap between genes downregulated during early CtBP2 induction (days 0–3) and genes upregulated during early CtBP2 depletion (days 9–12). (**D**) Venn diagram showing the overlap between genes downregulated during prolonged CtBP2 induction (days 3–9) and genes upregulated during late CtBP2 depletion (days 12–18). (**E**, **F**) Temporal expression dynamics of rapid- and delayed-response genes upon CtBP2 induction or depletion. Individual gene trajectories (thin lines) and the mean ± SEM (bold lines with error bars) are shown for Rapid response genes (*N* = 90, **E**) and delayed response genes (*N* = 49, **F**). (**G**) Heatmap showing the expression profiles of selected rapid and delayed response genes. (**H**) Box plot showing expression changes of rapid- and delayed-response genes in WT versus *Ctbp1/2* DKO ESCs and in *Dux* KO versus *Ctbp1/2/Dux* TKO ESCs. In each box plot, the center line indicates the median, the lower and upper bounds of the box indicate the 25th and 75th percentiles, respectively, and the lower and upper whisker ends indicate the smallest and largest values within 1.5× the interquartile range. Statistical significance was assessed by two-sided Wilcoxon rank-sum tests followed by Benjamini–Hochberg correction for multiple comparisons. *P < 0.05, **P < 0.01, ***P < 0.001; n.s., not significant. Exact adjusted *P* values for the annotated comparisons are: Rapid versus Delayed in *Ctbp1/2* DKO − WT, *P* = 1.3 × 10^−5^; Rapid *Ctbp1/2* DKO − WT versus Rapid *Ctbp1/2/Dux* TKO − *Dux* KO, *P* = 5.27 × 10^−4^; Delayed *Ctbp1/2* DKO − WT versus Delayed *Ctbp1/2/Dux* TKO − Dux KO, *P* = 1.3 × 10^−5^; Rapid versus Delayed in *Ctbp1/2/Dux* TKO − Dux KO, *P* = 6.0 × 10^−6.^ Complete pairwise adjusted *P* values are provided in Source Data. Source data are available online for this figure.

To monitor the dynamics of ZGA-associated gene expression in response to CtBP2 induction and depletion in ESCs, we established *Ctbp1/2* DKO ESCs carrying a doxycycline-inducible CtBP2 transgene. Using this system, we analyzed the expression of representative ZGA-related genes by RT-qPCR. *Dux*, *Mervl*, *Zscan4c*, and *Zfp352* were markedly downregulated between day 3 and day 9 following CtBP2 induction and subsequently reactivated from day 12 to day 18 upon CtBP2 depletion (Fig. 4A). In contrast, *Pramel7* responded more rapidly, showing marked downregulation between day 0 and day 3, and rapid reactivation between day 9 and day 12.

To distinguish between rapid and delayed transcriptional responses, we performed RNA-seq at defined time points. Principal component analysis (PCA) revealed that short-term CtBP2 induction (days 0–3) and depletion (days 9–12) drove opposing changes along the PC1 axis, whereas long-term induction (days 3–9) and depletion (days 12–18) contributed to opposite shifts along PC2 (Fig. 4B). By identifying the overlap between genes downregulated during CtBP2 induction and those upregulated during CtBP2 depletion, we identified two kinetically distinct groups: rapid-response genes, including major ZGA genes (*Pramel7*, *Gata1*, *Phlda2*) and maternal factors (*Klf17*, *Pramel13*) (Fig. 4C; Dataset EV1), and delayed-response genes, comprising minor ZGA genes such as *Zscan4*, *Usp17l* family genes and *Gm4340* (Fig. 4D; Dataset EV1). Temporal trajectory analysis showed that rapid-response genes (*N* = 90) were immediately repressed upon CtBP2 induction (days 0–3) and quickly reactivated following CtBP2 depletion (days 9–12) (Fig. 4E). By contrast, delayed-response genes (*N* = 49) displayed gradual downregulation during CtBP2 expression and delayed recovery upon CtBP2 depletion (Fig. 4F). Heatmap analysis illustrated their distinct temporal expression patterns (Fig. 4G).

Finally, to assess the DUX dependency of these gene sets, we compared gene expression changes induced by *Ctbp1/2* DKO in WT and *Dux* KO backgrounds. Rapid-response genes were similarly upregulated in both backgrounds, whereas the induction of delayed-response genes was significantly attenuated in the absence of DUX (Fig. 4H). Collectively, these results indicate that CtBP1/2 restrains two transcriptional cascades with distinct temporal kinetics: a rapidly responding DUX-independent program linked to major ZGA and maternal genes, and a delayed DUX-dependent program linked to minor ZGA genes.

### CtBP2 directly represses the major ZGA-associated genes *Pramel7* and *Gata1* in ESCs

**Figure 5:**
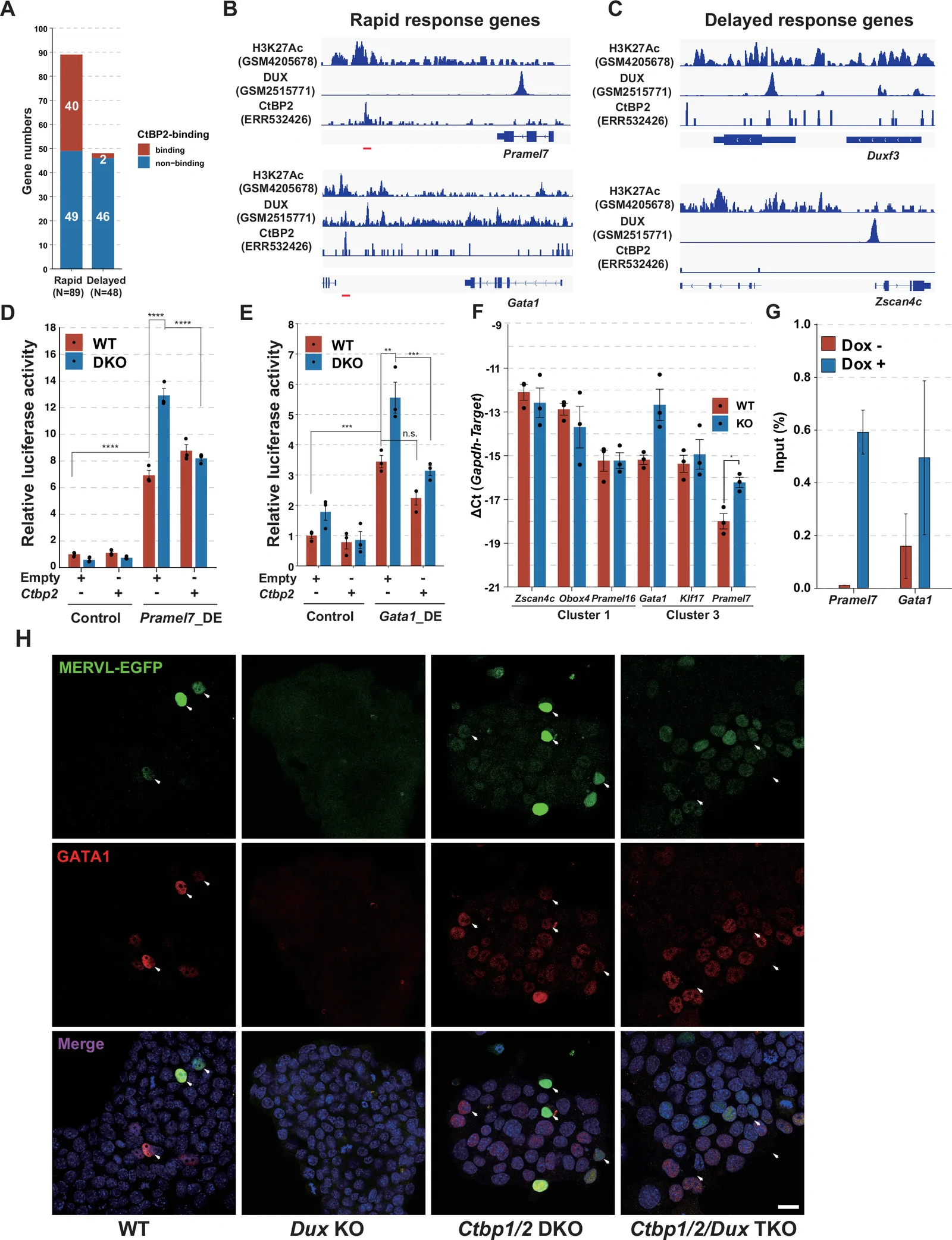
CtBP2 directly represses the major ZGA-associated genes *Pramel7* and *Gata1* in ESCs. (**A**) Proportion of CtBP2-binding genes among rapid- and delayed-response genes based on public CtBP2 ChIP-seq data. CtBP2 peaks were assigned to nearby genes using the Genomic Regions Enrichment of Annotations Tool (GREAT) under the “two nearest genes” rule, with a maximum extension of 1 Mb. Gene numbers are shown in the stacked bars. Statistical significance was assessed by a two-sided Fisher’s exact test. Exact *P* value: rapid-response genes versus delayed-response genes, *P* = 1.67 × 10^−7^. (**B**, **C**) Genome browser views showing H3K27Ac, DUX, and CtBP2 ChIP-seq signals at representative rapid-response gene loci (**B**) and delayed-response gene loci (**C**). Red horizontal bars indicate distal regulatory regions selected for Luciferase assays. (**D**, **E**) Luciferase reporter assays using distal regulatory regions from the *Pramel7* (**D**) or *Gata1* (**E**) loci in wild-type (WT) and *Ctbp1/2* DKO ESCs with either empty vector or CtBP2 expression vector. Bars indicate mean relative luciferase activity from *n* = 3 biological replicates, error bars indicate SEM, and dots indicate individual replicate values. Statistical significance was assessed by one-way ANOVA followed by Tukey’s HSD multiple-comparison test. ****P < 0.0001; n.s., not significant. (**D**) Exact adjusted *P* values for the annotated comparisons are: Control empty WT versus *Pramel7*_DE empty WT, *P* = 4.04 × 10^−9^; *Pramel7*_DE empty WT versus *Pramel7*_DE empty DKO, *P* = 3.48 × 10^−9^; *Pramel7*_DE empty DKO versus *Pramel7*_DE *Ctbp2* DKO, *P* = 1.08 × 10^−7^. (**E**) Exact adjusted *P* values for the annotated comparisons are: Control empty WT versus *Gata1*_DE empty WT, *P* = 2.17 × 10^−4^; *Gata1*_DE empty WT versus *Gata1*_DE empty DKO, *P* = 1.07 × 10^−3^; *Gata1*_DE empty WT versus *Gata1*_DE *Ctbp2* WT, *P* = 0.0947; *Gata1*_DE empty DKO versus *Gata1*_DE *Ctbp2* DKO, *P* = 2.51 × 10^−4^. Complete pairwise adjusted *P* values are provided in Source Data. (**F**) RT-qPCR analysis of representative Cluster 1 and Cluster 3 genes in WT and *Suz12* KO ESCs. ΔCt values were calculated relative to *Gapdh*. Bars indicate mean ΔCt values from *n* = 3 biological replicates, error bars indicate SEM, and dots indicate individual replicate values. Statistical significance was assessed separately for each gene by two-sided Welch’s *t* tests. *P < 0.05; n.s., not significant. Exact *P* value for the annotated comparison is: *Pramel7*, WT versus *Suz12* KO, *P* = 0.0187. Complete *P* values are provided in the Source Data. (**G**) CtBP2 ChIP–qPCR analysis using an anti-FLAG antibody at the *Pramel7* and *Gata1* loci in ESCs with or without doxycycline-induced FLAG-CtBP2 expression. ChIP signals are shown as a percentage of input. Bars indicate the mean of technical duplicate qPCR measurements, and error bars indicate SEM. No statistical test was performed. (**H**) Immunofluorescence analysis of WT, *Dux* KO, *Ctbp1/2* DKO, and *Ctbp1/2/Dux* TKO ESCs stained with anti-GFP antibody to detect MERVL-EGFP (green) and anti-GATA1 antibody (red). Nuclei were counterstained with DAPI. Arrowheads indicate representative MERVL-EGFP-positive cells. Scale bar, 10 μm. Source data are available online for this figure.

To examine whether CtBP2 directly regulates major ZGA-associated genes in ESCs, we analyzed the CtBP2 occupancy at rapid- and delayed-response genes using a public ChIP-seq dataset (Kim et al, 2015). Genomic peaks were annotated to nearby genes using the Genomic Regions Enrichment of Annotations tools (GREAT) with the “two nearest genes” setting (McLean et al, 2010), which assigns each regulatory region to the two closest transcription start sites (TSSs) within 1 Mb. Approximately half of the rapid-response genes (40 out of 89), but only 2 out of 48 delayed-response genes, were associated with CtBP2-binding peaks (Fig. 5A; Dataset EV1). Genome browser views of H3K27Ac (Zhang et al, 2020), a marker for active enhancers, DUX (Hendrickson et al, 2017), and CtBP2 occupancy showed that CtBP2 was enriched at regulatory regions of rapid-response genes such as *Pramel7* and *Gata1*, whereas DUX was enriched at delayed-response loci such as *Duxf3* and *Zscan4c* (Fig. 5B,C).

To functionally validate the repressive activity of CtBP2-bound regions, we performed luciferase reporter assays using distal enhancer fragments derived from the *Pramel7* and *Gata1* loci in WT and *Ctbp1/2* DKO ESCs. The activity of each enhancer was elevated in *Ctbp1/2* DKO ESCs and was suppressed upon CtBP2 re-expression (Fig. 5D,E), supporting the idea that CtBP2 directly represses *Pramel7* and *Gata1* transcription through their distal enhancer elements in ESCs. Because we have previously shown that CtBP1/2 cooperate with SUZ12, a component of the PRC2 complex, to repress target genes (Yamamoto et al, 2020), we examined the expression of genes belonging to Clusters 1 and 3 in WT and *Suz12* KO ESCs. Expression of Cluster 1 genes remained largely unchanged, whereas *Gata1* and *Pramel7* were significantly upregulated by *Suz12* KO in ESCs (Fig. 5F). CtBP2 binding at the *Pramel7* and *Gata1* distal enhancers was further confirmed by ChIP–qPCR analysis (Fig. 5G).

Immunofluorescence analysis further showed that MERVL-EGFP-positive cells expressed GATA1 in WT ESCs, whereas GATA1 expression was abolished in *Dux* KO ESCs (Fig. 5H). Consistent with the FACS profiles described earlier (Fig. 2A), *Ctbp1/2* DKO ESCs contained both GFP+ and GFP + ++ populations, and both populations expressed GATA1. Deletion of *Dux* in *Ctbp1/2* DKO ESCs selectively eliminated the GFP-strong/GATA1-positive cells, whereas GFP-weak-positive, GATA1-positive cells persisted. These findings suggest that Ctbp1/2 DKO ESCs comprise two transcriptionally distinct MERVL-EGFP-positive populations: a DUX-dependent, GFP + + + , GATA1-positive subset corresponding to classical MERVL-high 2CLCs, and a DUX-independent, GFP + , GATA1-positive subset associated with major ZGA-related gene activation.

### PRAMEL7 controls subsets of DUX-dependent and DUX-independent ZGA-associated genes

**Figure 6:**
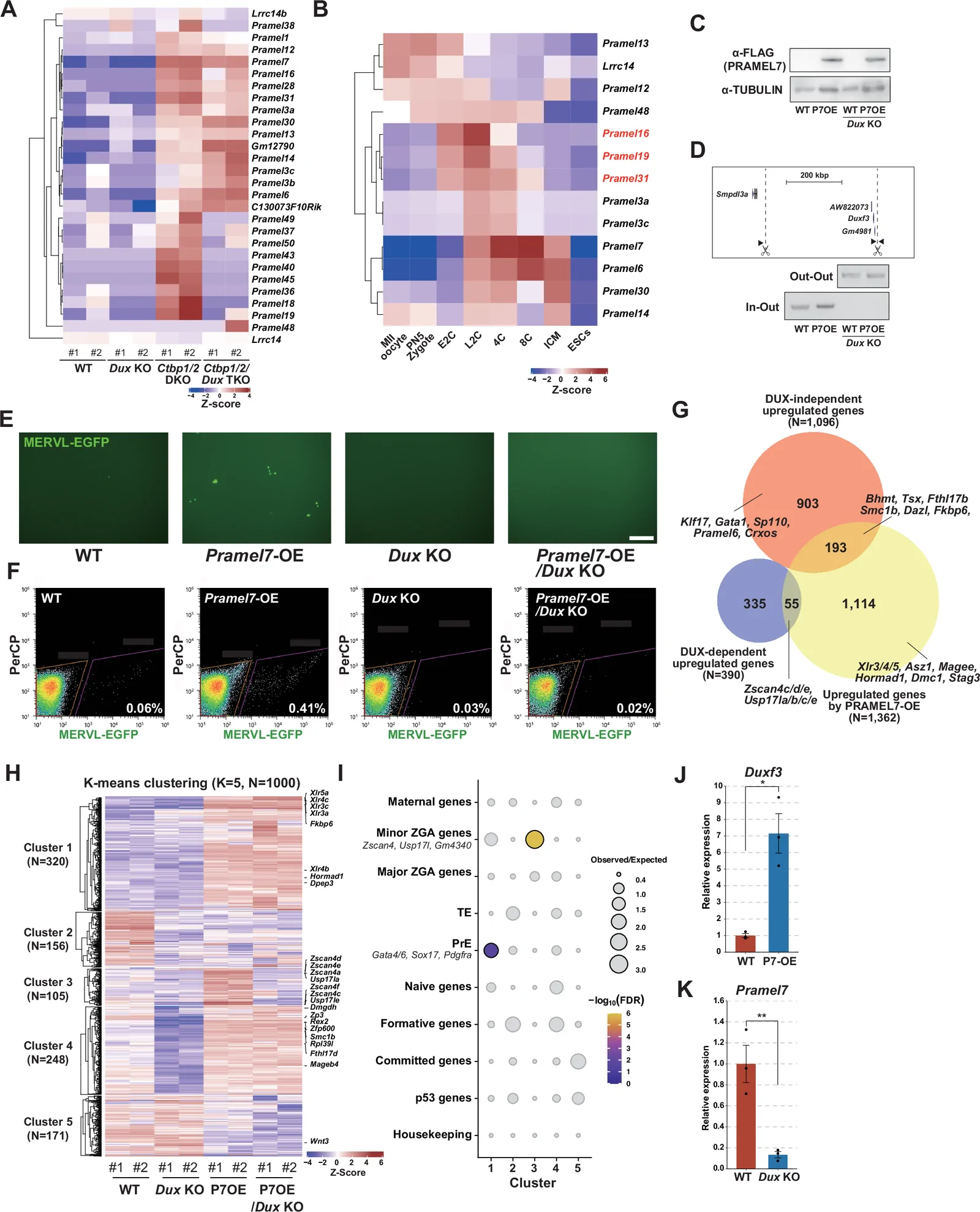
PRAMEL7 controls subsets of ZGA-associated genes through DUX-dependent and -independent mechanisms. (**A**) Hierarchical clustering heatmap of PRAME family gene expression from RNA-seq of wild-type (WT), *Dux* KO, *Ctbp1/2* DKO, and *Ctbp1/2*/*Dux* TKO ESCs. (**B**) Expression dynamics of PRAME family genes during early mouse embryogenesis (Zhang et al, 2016). Red letters indicate DUX-dependent genes. (**C**) Western blot analysis of FLAG-tagged PRAMEL7 protein levels in WT, PRAMEL7-overexpressing (P7OE), *Dux* KO, and P7OE/*Dux* KO ESCs. (**D**) Genotype PCR confirming deletion of the *Dux* cluster using primer sets flanking the gRNA target site. (**E**) Fluorescence images showing MERVL-EGFP signals in WT, P7OE, *Dux* KO, and P7OE/*Dux* KO ESCs. Scale bar, 200 μm. (**F**) FACS analysis showing the percentage of MERVL-EGFP-positive cells in WT, P7OE, *Dux* KO, and P7OE/*Dux* KO ESCs. (**G**) Venn diagram showing the overlap of genes upregulated by *Ctbp1/2* DKO (with or without DUX) and those induced by PRAMEL7 overexpression. (**H**) K-means clustering (*K* = 5) of the top 1000 most variable genes from RNA-seq of WT, P7OE, *Dux* KO, and P7OE/*Dux* KO ESCs. (**I**) Gene set enrichment analysis of each cluster identified in (**H**). (**J**) RT-qPCR analysis of *Duxf3* expression in WT and P7OE ESCs. ΔCt values were calculated relative to *Gapdh*, and error bars indicate the standard error of means (SEM) from three biological replicates. Statistical significance was assessed by a two-sided Welch’s *t* test. *P < 0.05. WT versus *Pramel7* OE, *P* = 0.0345. (**K**) RT-qPCR analysis of *Pramel7* expression in WT and *Dux* KO ESCs. ΔCt values were calculated relative to *Gapdh*, and error bars indicate the standard error of means (SEM) from three biological replicates. Statistical significance was assessed by a two-sided Welch’s *t* test using ΔCt values. **P < 0.01. Exact *P* value: WT versus *Dux* KO, *P* = 0.00439. Source data are available online for this figure.

*Pramel7* belongs to the PRAME (preferentially expressed antigen in melanoma) gene family, whose expression is normally restricted to the testis and cancers (Kern et al, 2021). Because the PRAME family is one of the largest gene families in the mouse genome (Church et al, 2009), we examined its regulation in wild-type (WT), *Dux* KO, *Ctbp1/2* DKO, and *Ctbp1/2/Dux* TKO ESCs. A phylogenetic tree constructed from amino acid sequences annotated in mm39 revealed that PRAME family genes are organized into six chromosomal clusters, with high intracluster similarity (Appendix Fig. S3A,B). RNA-seq analysis showed that most PRAME family genes, except for *Lrrc14*, *Lrrc14b*, and *Pramel48*, were markedly upregulated in *Ctbp1/2* DKO ESCs (Fig. 6A). Based on expression patterns across WT, *Dux* KO, *Ctbp1/2* DKO and *Ctbp1/2/Dux* TKO ESCs, PRAME genes were classified into four groups: (1) genes unaffected by *Ctbp1/2* DKO (e.g., *Lrrc14*); (2) genes further upregulated by *Dux* KO (e.g., *Pramel14/30*); (3) genes suppressed by *Dux* KO (e.g., *Pramel16/19/31*, DUX-dependent); and (4) genes unaffected by *Dux* KO (e.g., *Pramel7/12*, DUX-independent). Re-analysis using published single-cell RNA-seq data from early embryonic stages (Zhang et al, 2016) revealed that DUX-dependent PRAME genes (e.g., *Pramel16/19/31*) are transiently induced at the mid-2-cell stage (Fig. 6B). In contrast, DUX-independent PRAME genes could be further subdivided into two categories: (i) *Pramel6/7/30*, which are upregulated from mid-2-cell and peak at ICM before being silenced in ESCs; and (ii) *Pramel12/13*, which are maternally expressed and downregulated by the 4-cell stage.

Given the DUX-independent function of *Pramel7* and its known importance in early mouse development (Graf et al, 2017), we generated ESCs overexpressing *Pramel7* (P7OE) in both WT and *Dux* KO backgrounds (Fig. 6C,D). *Pramel7* overexpression modestly increased the fraction of MERVL-EGFP-positive cells in WT ESCs (Fig. 6E,F), although the increase was much smaller than that observed in *Ctbp1/2* DKO ESCs. In contrast to Ctbp1/2 DKO ESCs, in which a MERVL-EGFP-weak-positive population (GFP + ) persisted even after Dux deletion (Fig. 2A), *Pramel7* overexpression failed to generate either GFP + ++ or GFP+ cells in the *Dux* KO background. These results suggest that the residual GFP+ population induced by Ctbp1/2 depletion without DUX function arises independently of PRAMEL7.

We next compared genes upregulated by PRAMEL7 with those upregulated by *Ctbp1/2* DKO in either DUX-dependent or -independent contexts. Venn diagram analysis revealed that a subset of genes activated in a DUX-dependent manner (55 out of 390) as well as in a DUX-independent manner (193 out of 1096) upon *Ctbp1/2* DKO were also upregulated by PRAMEL7 overexpression (Fig. 6G; Dataset EV1). To further investigate the transcriptional programs controlled by PRAMEL7, we performed K-means clustering (*K* = 5) of the top 1000 most variable genes from RNA-seq of WT, *Dux* KO, P7OE, and P7OE/*Dux* KO ESCs (Fig. 6H). Cluster 1 contained PRAMEL7-induced genes unaffected by *Dux* KO, including germline-specific and DNA methylation-repressed genes (e.g., *Xlr3/4/5*, *Hormad1*) (Escalier et al, 2002; Shin et al, 2010), and primitive endoderm-associated genes were enriched in this cluster (Fig. 6I). Cluster 3 genes, including *Zscan4* and *Usp17l* family members, were upregulated by PRAMEL7 overexpression but this induction was abolished in the *Dux* KO background and enriched in minor ZGA genes, indicating that PRAMEL7 promotes the expression of minor ZGA genes through DUX, consistent with increased *Duxf3* expression (Fig. 6J). Cluster 4 comprised genes that were downregulated in *Dux* KO ESCs relative to WT ESCs but remained expressed in P7OE/Dux KO ESCs. Because *Pramel7* expression was significantly reduced upon *Dux* KO in WT ESCs, but remained strongly upregulated, and was further increased, in the *Ctbp1/2* DKO background even in the absence of DUX (Fig. 6K), this result suggests that at least part of the *Dux* KO-dependent downregulation of Cluster 4 genes may be attributable to reduced *Pramel7* expression. Taken together, these results identify PRAMEL7 as a modulator that links DUX-dependent and -independent transcriptional programs associated with the 2C-like state.

### PRAMEL7 overexpression induces post-ZGA-associated genes through UHRF1 repression

**Figure 7:**
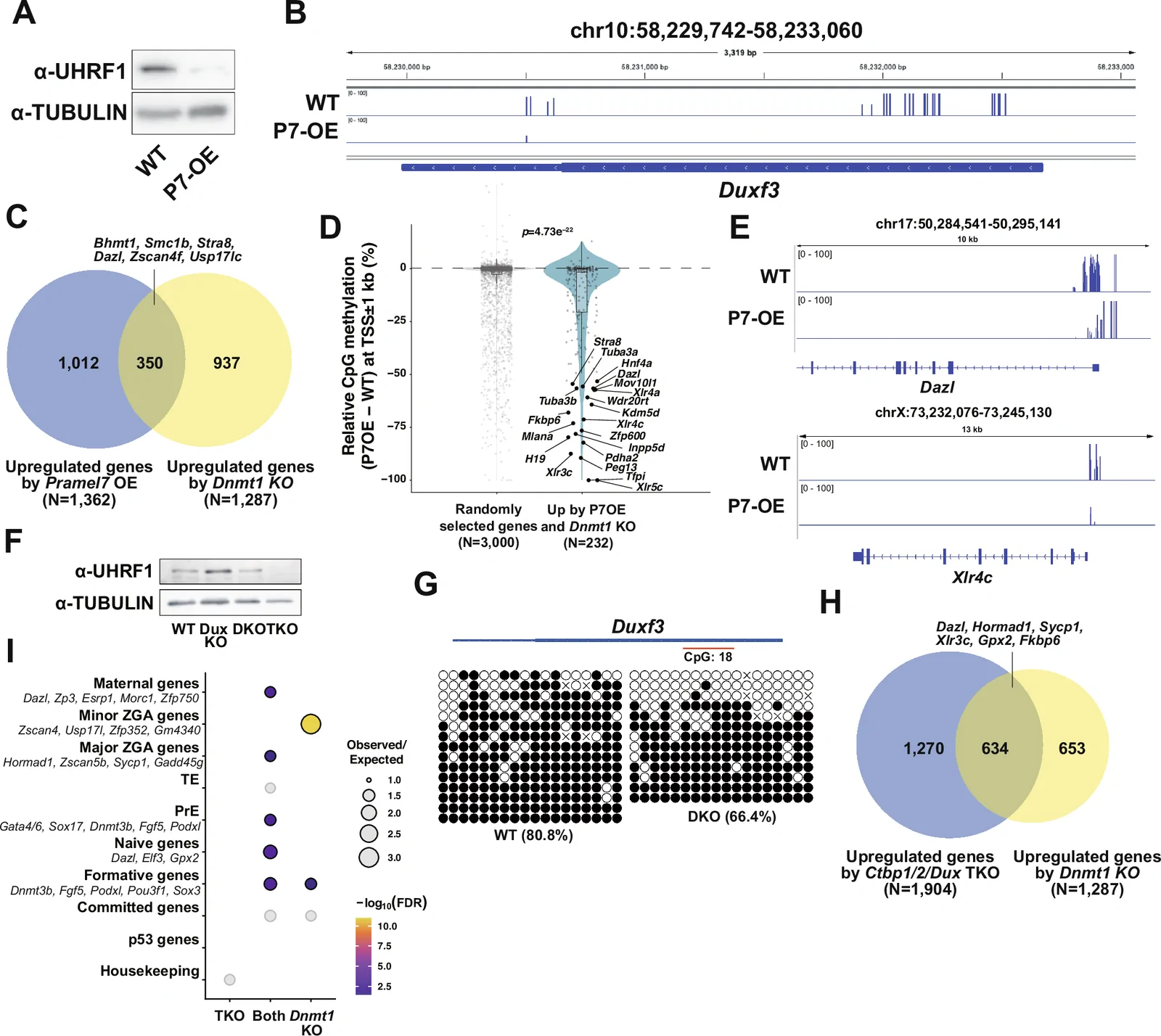
PRAMEL7 overexpression promotes DNA demethylation-linked activation of post-ZGA-associated genes through UHRF1 downregulation. (**A**) Western blot analysis of UHRF1 protein levels in wild-type (WT) and PRAMEL7-OE (P7OE) ESCs. α-TUBULIN was used as a loading control. (**B**) Representative genome browser view of RRBS methylation profiles at the *Duxf3* locus in WT and P7OE ESCs. (**C**) Venn diagram showing the overlap between genes upregulated by P7OE and those upregulated in *Dnmt1 KO* ESCs. Representative overlapping genes are indicated. (**D**) Violin plots showing relative CpG methylation changes at TSS ± 1 kb regions in randomly selected genes and genes upregulated in both P7OE and *Dnmt1* KO ESCs. Relative CpG methylation was calculated as P7OE − WT. Dots indicate individual genes; boxes indicate the median and interquartile range. In the embedded box plots, the center line indicates the median, the lower and upper bounds of the box indicate the 25th and 75th percentiles, respectively, and the lower and upper whisker ends indicate the smallest and largest values within 1.5× the interquartile range. Selected genes with large methylation decreases are labeled. Distributions were compared using a two-sided Wilcoxon rank-sum test. Exact *P* value: randomly selected genes versus genes upregulated in both P7OE and *Dnmt1* KO, *P* = 4.73 × 10^−22^. (**E**) Representative genome browser views of RRBS methylation profiles at the *Dazl* and *Xlr4c* loci in WT and P7OE ESCs. (**F**) Western blot analysis of UHRF1 protein levels in WT, *Dux* KO, *Ctbp1/2* DKO, and *Ctbp1/2/Dux* TKO ESCs. α-TUBULIN was used as a loading control. (**G**) Bisulfite sequencing analysis of the *Duxf3* locus in WT and *Ctbp1/2* DKO ESCs. Filled circles indicate methylated CpGs, open circles indicate unmethylated CpGs, and crosses indicate undetermined CpGs. Percentages indicate the overall methylation level. (**H**) Venn diagram showing the overlap between genes upregulated in *Ctbp1/2/Dux* TKO ESCs and those upregulated in *Dnmt1* KO ESCs. Representative overlapping genes are indicated. (**I**) Enrichment analysis of selected developmental gene sets among genes upregulated only in *Ctbp1/2/Dux* TKO ESCs (TKO), genes commonly upregulated in *Ctbp1/2/Dux* TKO and *Dnmt1* KO ESCs (Both), and genes upregulated only in *Dnmt1* KO ESCs. Dot size indicates the observed/expected ratio, and color indicates −log10(FDR). Source data are available online for this figure.

Overexpression of PRAMEL7 decreases the protein level of UHRF1, which is required for the maintenance of DNA methylation (Fig. 7A), consistent with a previous study (Graf et al, 2017). Reduced Representation Bisulfite Sequencing (RRBS) revealed that high DNA methylation around the *Duxf3* locus was markedly reduced by the overexpression of PRAMEL7 (Fig. 7B), suggesting that the upregulation of *Duxf3* by PRAMEL7 overexpression occurs through DNA demethylation. Next, to determine whether genes upregulated by PRAMEL7 overexpression depend on DNA demethylation, we compared genes upregulated in PRAMEL7-overexpressing (P7OE) ESCs with those upregulated in *Dnmt1* KO ESCs based on the RNA-seq data (Fig. 7C; Dataset EV1). Notably, 350 of 1,362 genes upregulated by PRAMEL7-OE ESCs (25.7%) were also upregulated in *Dnmt1* KO ESCs. To examine whether genes commonly upregulated in P7OE and *Dnmt1* KO ESCs are associated with altered DNA methylation, we compared CpG methylation levels at TSS ± 1 kb between P7OE and WT ESCs (Fig. 7D). Of the 350 overlapping genes, 232 could be mapped to the methylation dataset and were included in the analysis. These genes showed significantly lower CpG methylation than randomly selected genes (*N* = 3000), indicating that genes commonly upregulated in P7OE and *Dnmt1* KO ESCs are preferentially associated with promoter-proximal DNA hypomethylation. Consistent with this trend, representative loci such as *Dazl* and *Xlr4c* displayed reduced CpG methylation in P7OE ESCs compared with WT ESCs (Fig. 7E).

Next, to determine whether *Pramel7* upregulation in *Ctbp1/2*-deficient backgrounds contributes to transcriptional regulation through UHRF1 degradation, we examined UHRF1 protein levels in WT, *Dux* KO, *Ctbp1/2* DKO, and *Ctbp1/2/Dux* TKO ESCs. UHRF1 expression levels were decreased both in DKO and TKO ESCs, with more pronounced reduction in TKO ESCs, consistent with further upregulation of PRAMEL7 in TKO ESCs (Figs. 7F and 3A). Bisulfite-sequencing analysis at the *Duxf3* locus further revealed a modest reduction in DNA methylation in DKO ESCs compared with WT ESCs (Fig. 7G), suggesting that DNA demethylation may contribute to *Dux* upregulation and the associated induction of minor ZGA genes in DKO ESCs. To identify genes activated through DNA demethylation in a DUX-independent manner upon *Ctbp1/2* loss, we compared genes upregulated in *Ctbp1/2/Dux* TKO ESCs with those upregulated in *Dnmt1* KO ESCs. Among the 1,904 genes upregulated in *Ctbp1/2/Dux* TKO ESCs, 634 genes (33.3%) were also upregulated in *Dnmt1* KO ESCs (Fig. 7H; Dataset EV1). This overlap suggests that PRAMEL7-mediated UHRF1 downregulation may activate a subset of *Ctbp1/2* loss-induced, DUX-independent genes through DNA demethylation. Consistent with this interpretation, these overlapping genes were enriched for maternally expressed genes, major ZGA genes, and post-ZGA-associated genes (Fig. 7I).

### PRAMEL7-mediated UHRF1 downregulation contributes to DNA methylation-dependent regulation of post-ZGA-associated genes

**Figure 8:**
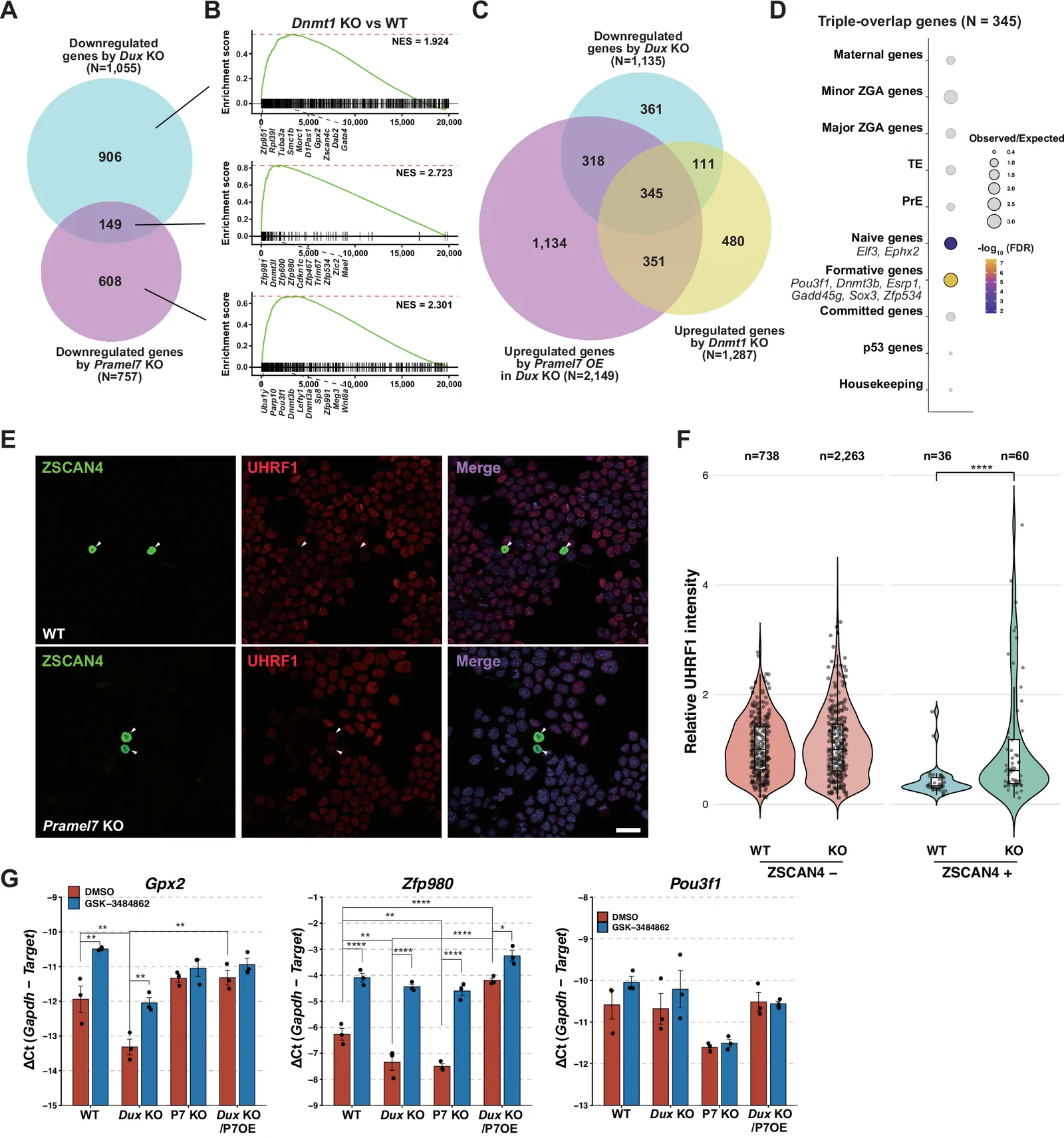
PRAMEL7-mediated UHRF1 downregulation contributes to DNA methylation-dependent regulation of post-ZGA-associated genes. (**A**) Venn diagram showing the overlap between genes downregulated in *Dux* KO ESCs and those downregulated in *Pramel7* KO ESCs. (**B**) Gene set enrichment analysis of genes upregulated in *Dnmt1* KO ESCs among the three categories shown in (**A**): *Dux* KO-specific, commonly downregulated, and *Pramel7* KO-specific genes. NES values are indicated. (**C**) Venn diagram showing the overlap among genes downregulated in *Dux* KO ESCs, genes upregulated by *Pramel7* overexpression in the *Dux* KO background, and genes upregulated in *Dnmt1* KO ESCs. (**D**) Enrichment analysis of selected developmental gene sets among the triple-overlap genes shown in (**C**). Dot size indicates the observed/expected ratio, and color indicates −log10(FDR). (**E**) Representative immunofluorescence images of ZSCAN4 and UHRF1 in WT and *Pramel7* KO ESCs. Arrows indicate ZSCAN4-positive cells. Scale bar, 20 μm. (**F**) Quantification of relative UHRF1 intensity in ZSCAN4-negative and ZSCAN4-positive cells from WT and *Pramel7* KO ESCs. Cells were classified as ZSCAN4-negative or ZSCAN4-positive based on ZSCAN4 mean intensity. UHRF1 intensity was normalized to the genotype-matched median UHRF1 intensity of ZSCAN4-negative cells. Violin plots show the distribution of normalized single-cell UHRF1 intensity. Boxes indicate the median and interquartile range, and dots indicate individual cells. In the embedded box plots, the center line indicates the median, the lower and upper bounds of the box indicate the 25th and 75th percentiles, respectively, and the lower and upper whisker ends indicate the smallest and largest values within 1.5× the interquartile range. The number of analyzed cells is indicated above each group. Statistical significance was assessed by two-sided Wilcoxon rank-sum tests followed by Bonferroni correction for the two comparisons. Exact adjusted *P* values are: ZSCAN4-negative WT versus *Pramel7* KO, *P* = 1.00; ZSCAN4-positive WT versus *Pramel7* KO, *P* = 1.3 × 10^−5^. (**G**) RT-qPCR analysis of representative genes from the three categories shown in (**A**) after treatment with the DNMT1 inhibitor GSK-3484862 or vehicle control DMSO. *Gpx2*, *Zfp980*, and *Pou3f1* represent *Dux* KO-specific, commonly downregulated, and *Pramel7* KO-specific genes, respectively. ΔCt values were calculated as Ct (*Gapdh*) – Ct (target gene). Bars indicate mean ± SEM from biological triplicates, and dots indicate individual replicates. For visualization, bars were drawn upward from gene-specific baselines, while statistical analyses were performed using the original ΔCt values. For each gene, statistical significance was assessed by two-way ANOVA with Cell and Treatment as factors, followed by planned comparisons among cell types under the DMSO condition, among cell types under the GSK-3484862 condition, and between DMSO and GSK-3484862 within each cell type. *P* values were adjusted using Holm correction within each gene. Exact adjusted *P* values for the annotated comparisons are as follows. For *Gpx2*: WT DMSO versus *Dux* KO DMSO, *P* = 0.00404; *Dux* KO DMSO versus *Dux* KO/P7OE DMSO, *P* = 0.000101; WT DMSO versus WT GSK-3484862, *P* = 0.00266; *Dux* KO DMSO versus *Dux* KO GSK-3484862, *P* = 0.00768. For *Zfp980*: WT DMSO versus *Dux* KO DMSO, *P* = 0.00597; WT DMSO versus P7 KO DMSO, *P* = 0.00223; WT DMSO versus *Dux* KO/P7OE DMSO, *P* = 7.60 × 10^−6^; WT DMSO versus WT GSK-3484862, *P* = 4.31 × 10^−6^; *Dux* KO DMSO versus *Dux* KO GSK-3484862, *P* = 9.65 × 10^−8^; P7 KO DMSO versus P7 KO GSK-3484862, *P* = 9.65 × 10^−8^; *Dux* KO/P7OE DMSO versus *Dux* KO/P7OE GSK-3484862, *P* = 0.0145. For panels with statistical annotations, significance labels are defined as follows: *P < 0.05; **P < 0.01; ***P < 0.001; ****P < 0.0001; n.s., not significant. Complete statistical results are provided in the accompanying README and Source Data files. Source data are available online for this figure.

To investigate the functional relationship between DUX and PRAMEL7, we generated *Pramel7* KO ESCs and compared genes downregulated in *Dux* KO and *Pramel7* KO ESCs and visualized their overlap using a Venn diagram (Fig. 8A; Dataset EV1). Of the 1,055 genes downregulated in *Dux* KO ESCs, 149 genes (14.1%) were also downregulated in *Pramel7* KO ESCs. To examine whether DNA methylation contributes to the downregulation of these genes, we divided them into three categories―*Dux* KO-specific, commonly downregulated, and *Pramel7* KO-specific genes―and assessed their enrichment among genes upregulated in *Dnmt1* KO ESCs (Fig. 8B). All three categories showed significant enrichment, with the commonly downregulated genes exhibiting the strongest enrichment. Next, we compared genes downregulated in *Dux* KO ESCs, genes upregulated by *Pramel7* overexpression in *Dux* KO ESCs, and genes upregulated in *Dnmt1* KO ESCs using a Venn diagram. Among the 663 *Dux* KO-downregulated genes rescued by *Pramel7* overexpression, 345 genes (52.0%) were also upregulated in *Dnmt1* KO ESCs (Fig. 8C; Dataset EV1), suggesting that a substantial fraction of PRAMEL7-rescued transcriptional defects in *Dux* KO ESCs is associated with DNA methylation-dependent repression. These triple-overlapped genes were enriched for naive and formative pluripotency-associated gene sets (Fig. 8D).

Because proteasome-mediated degradation of UHRF1 has been shown to occur in 2CLCs (Dan et al, 2017), we compared UHRF1 protein levels in ZSCAN4-positive 2CLCs between WT and *Pramel7* KO ESCs. Notably, although ZSCAN4-positive cells were still present in *Pramel7* KO ESCs, UHRF1 downregulation was significantly impaired in the ZSCAN4-positive population, but not completely abolished (Fig. 8E,F). Finally, we examined whether pharmacological inhibition of DNMT1 by the DNMT1 inhibitor GSK-3484862 could rescue the representative genes from the three categories: *Gpx2* (*Dux* KO-specific), *Zfp980* (commonly downregulated), and *Pou3f1* (*Pramel7* KO-specific). GSK-3484862 restored the expression of *Gpx2* in *Dux* KO ESCs and *Zfp980* in both *Dux* KO and *Pramel7* KO ESCs, consistent with DNA methylation-dependent repression. In contrast, Pou3f1 was not rescued by GSK-3484862 but was restored by *Pramel7* overexpression (Fig. 8G). These results suggest that impaired UHRF1 downregulation in *Pramel7* KO 2 CLCs affects the DNA demethylation-dependent activation of a subset of post-ZGA-associated genes in ESC cultures.

## Discussion

**Figure 9:**
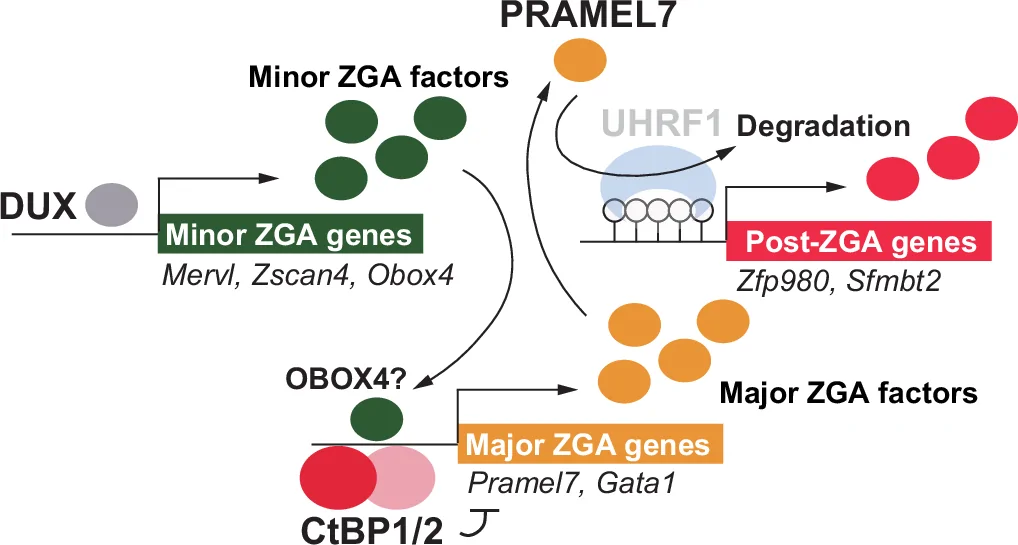
A schematic model for the regulation of early embryo-associated transcriptional programs by DUX, CtBP1/2, and PRAMEL7.

In this study, we demonstrate that the loss of *Ctbp1/2* in ESCs activates both DUX-dependent and DUX-independent transcriptional cascades associated with 2C-like and early embryonic transcriptional programs (Fig. 9). Most DUX-dependent genes activated upon *Ctbp1/2* depletion correspond to minor ZGA genes transiently expressed at the two-cell (2 C) stage. In contrast, DUX-independent genes upregulated upon *Ctbp1/2* loss can be classified into major ZGA genes and post-ZGA-associated genes. Among them, *Pramel7* is directly upregulated by *Ctbp1/2* loss in a DUX-independent manner and further modulates both the DUX-dependent program, through *Dux* activation, and DUX-independent programs, at least in part through UHRF1 downregulation-mediated DNA demethylation. Collectively, these findings uncover a dual regulatory framework in which CtBP1/2 represses both DUX-mediated and DUX-independent transcriptional programs, providing new insights into the molecular mechanisms that constrain early embryogenesis-associated transcriptional states in ESCs.

Time-lapse recording revealed that the kinetics and cellular behavior of MERVL-EGFP-positive cells derived from *Ctbp1/2* DKO ESCs were distinct from those of 2 CLCs that spontaneously emerge from WT ESCs. In WT ESCs, MERVL-EGFP-positive cells rapidly acquired high EGFP intensity and frequently underwent cellular collapse, consistent with previous reports (Olbrich et al, 2021). In contrast, *Ctbp1/2* DKO ESCs rarely generated cells showing an abrupt increase in MERVL-EGFP intensity followed by rapid collapse. Instead, most MERVL-EGFP-positive cells in *Ctbp1/2* DKO ESCs emerged gradually, reached low to intermediate levels of MERVL-EGFP expression, and often retained the ability to undergo cell division. These observations suggest that loss of *Ctbp1/2* does not simply amplify the canonical, transient MERVL-high 2 CLC state observed in WT ESCs. Rather, *Ctbp1/2* deficiency appears to generate an altered MERVL-EGFP-positive state with slower activation kinetics and greater proliferative persistence. One possibility is that transcriptional programs induced by *Ctbp1/2* deficiency, including major ZGA-associated genes, attenuate abrupt entry into a fragile MERVL-high state and thereby allow MERVL-EGFP-positive cells to persist and proliferate. Consistent with this interpretation, genes associated with early and intermediate phases of 2CLC transition were upregulated in both *Ctbp1/2* DKO and *Ctbp1/2/Dux* TKO ESCs. This suggests that loss of *Ctbp1/2* can promote entry into an early or intermediate 2CLC-like transcriptional state independently of DUX. In contrast, full acquisition of the classical MERVL-high 2CLC state, characterized by strong MERVL-EGFP expression and robust activation of minor ZGA genes, still requires DUX. Thus, *Ctbp1/2* deficiency may uncouple early/intermediate 2CLC-associated transcriptional activation from terminal DUX-dependent 2CLC maturation.

Our data show that the loss of *Ctbp1/2* in ESCs upregulates major ZGA genes and post-ZGA-associated genes in a DUX-independent manner, highlighting an alternative regulatory axis operating in the mESC/2CLC system. Interestingly, recent studies have demonstrated that OBOX proteins, a family of PRD-like homeobox transcription factors, can compensate for DUX function by directly activating MERVL as well as both minor and major ZGA genes (Guo et al, 2024; Ji et al, 2023; Sakamoto et al, 2024). However, our transcriptome analysis revealed that only *Obox4* was upregulated in *Ctbp1/2* double-knockout (DKO) ESCs, and this upregulation was attenuated by *Dux* KO. These results suggest that the loss of *Ctbp1/2* regulates early developmental genes through pathways independent of both DUX and OBOX in ESCs. However, this conclusion should be limited to the mESC/2CLC system. In embryos, published RNA-seq data suggest that minor ZGA genes, including direct DUX targets, are activated with delayed kinetics in *Dux* KO embryos, whereas major ZGA genes such as *Pramel7* are still upregulated at the middle 2-cell stage (Guo et al, 2019). This difference may reflect the presence of maternally deposited activators, including OBOX family members (Ji et al, 2023), together with the progressive decline of maternal *Ctbp1/2* transcripts after fertilization (Zhang et al, 2016), which may help support DUX-independent activation of major ZGA genes in vivo.

By integrating transcriptome data from conditional *Ctbp2* KO ESCs with public CtBP2 ChIP-seq datasets, we identified direct CtBP2 target genes, including *Pramel7*, *Gata1*, and *Klf17*, and distinguished them from DUX-dependent regulatory genes. Furthermore, we found that MERVL-EGFP-weak cells in both *Ctbp1/2* DKO and *Ctbp1/2/Dux* TKO ESCs expressed GATA1, a direct CtBP2 target. Importantly, GATA1-positive cells were absent in *Dux* KO ESCs, suggesting that *Ctbp1/2* loss permits activation of a GATA1-positive MERVL-EGFP state even in the absence of DUX. Unlike previously characterized 2CLC repressors (Zhang et al, 2016), which primarily act through the repression of *Dux* or its upstream regulators, CtBP1/2 proteins directly repress major ZGA-associated genes, including *Pramel7* and *Gata1*, thereby constraining a DUX-independent regulatory arm in addition to the canonical DUX-dependent program.

The *PRAME* family is one of the most expanded gene families in mammals, and the mouse *Prame* gene family represents the third-largest multigene family in the genome (Chang et al, 2011). We showed that the disruption of *Ctbp1/2* activates the majority of *Prame* family genes in ESCs, some of which are upregulated by DUX, whereas others are induced directly by the loss of CtBP1/2. The positive regulators that drive DUX-independent activation of *Prame* family genes remain largely unknown. Notably, published RNA-seq data indicate that OBOX3/5, but not DUX, can activate *Pramel7* and *Pramef17* (*Pramel14*), both of which are upregulated by *Ctbp1/2* loss even in the absence of DUX. In contrast, *Pramel16*, *Gm12794* (*Pramel19*), and *A430089l19Rik* (*Pramel39*) require DUX for activation (Sakamoto et al, 2024). Because *Obox* family genes were silenced in *Ctbp1/2/Dux* TKO ESCs, it is possible that another positive regulator, distinct from OBOX proteins, is activated upon *Ctbp1/2* depletion or constitutively expressed in WT ESCs. Expression of *Prame* family genes has been observed in various cancer and germline cells (Kern et al, 2021), and their expression pattern resembles that of *Pou5f1* (Oct4) (Bortvin et al, 2003), suggesting that an OCT4-centered transcriptional network may function as a positive regulatory axis for *Prame* genes.

We further demonstrated that the overexpression of PRAMEL7 partially rescues the transcriptional abnormalities observed in *Dux* KO ESCs, in which *Pramel7* expression was markedly reduced. PRAMEL7 contributes to two distinct genetic programs: one involving minor ZGA genes through *Dux* upregulation, and another encompassing post-ZGA genes, including primitive endoderm, naive, and formative pluripotency-associated genes. Previous work showed that PRAMEL7 promotes global DNA demethylation by targeting UHRF1 for proteasomal degradation in ESCs (Graf et al, 2017). In addition, *Dux* expression is epigenetically repressed by DNA methylation maintained by the ZBTB24-CDCA7-HELLS axis (Guo et al, 2025). Our data support the idea that upregulation of *Pramel7* in *Ctbp1/2* DKO ESCs drives DNA demethylation-dependent transcriptional programs, including the activation of *Dux* and post-ZGA-associated genes. This interpretation is consistent with previous studies showing that global DNA demethylation induced by overexpression of the maternal factor DPPA3 promotes 2C-like transcriptional programs (Zhang et al, 2022). Notably, maternal PRAMEL15, another PRAME-family protein, promotes nuclear DNMT1 degradation and DNA demethylation in the 2C embryos (Tan et al, 2024). Although PRAMEL15 functions in two-cell embryos, *Pramel15* was not detectably expressed in published RNA-seq datasets of ESC-derived 2CLCs (Eckersley-Maslin et al, 2016) and was not upregulated in our *Ctbp1/2* DKO ESCs, suggesting that PRAMEL15 is unlikely to mediate the transcriptional effects observed in our ESC-based system. Together with these findings, our results suggest that PRAME-family proteins and maternal/early embryonic factors may converge on the maintenance DNA methylation machinery to facilitate the activation of early embryo-associated transcriptional programs. Because PRAMEL7 also promotes degradation of NuRD complex at chromatin to sustain pluripotency in ESCs (Rupasinghe et al, 2024), it will be important to explore whether NuRD degradation by PRAMEL7 contributes to the regulation of post-ZGA-associated transcriptional programs.

Although DUX is a master regulator of the transition from pluripotent ESCs to 2-cell-like cells, our results demonstrated that PRAMEL7 overexpression restores a substantial fraction of the transcriptional defects observed in *Dux* KO ESCs. A subset of *Prame* family genes is upregulated during the early phase of 2CLC emergence, traced by the *Zscan4* and MERVL reporter system based on scRNA-seq (Eckersley-Maslin et al, 2016; Rodriguez-Terrones et al, 2018), and MERVL-positive 2CLCs are eliminated by *Dux* KO (De Iaco et al, 2017). Our data further showed that *Pramel7* expression was significantly reduced upon *Dux* KO in WT ESCs but remained unaffected in *Ctbp1/2* DKO ESCs, indicating that loss of *Ctbp1/2* bypasses part of the *Dux* KO-associated transcriptional phenotype through *Pramel7* upregulation in ESCs. Taken together, we propose that DUX may orchestrate two distinct transcriptional programs in ESC-derived 2CLC states and early mouse embryos: (1) direct activation of minor ZGA genes (e.g., MERVL, *Zscan4c*, and *Obox4*), and (2) PRAMEL7-mediated transcriptional cascades. Although *Dux* KO ESCs exhibit a marked reduction in *Pramel7* expression, *Pramel7* is still upregulated in *Dux* KO embryos at the mid-2C stage, highlighting a context-dependent difference between ESC-derived 2CLC models and embryos.

Our findings highlight fundamental differences between in vitro induction of 2CLCs and in vivo regulation of ZGA, which are likely governed by maternal factors such as OBOX and CtBP2. To better characterize these DUX-independent cascades, dual-reporter ESC models (e.g., MERVL-tdTomato/*Gata1* or *Pramel7*-EGFP reporters) combined with single-cell RNA-seq would provide powerful approaches to dissect the molecular networks underlying 2CLC heterogeneity induced by *Ctbp1/2* loss. Furthermore, because our analyses were performed in the mESC/2CLC system, direct functional and transcriptomic analyses in embryos will be necessary to determine to what extent the CtBP1/2–PRAMEL7 axis identified here also operates in vivo. In addition, although *Ctbp1/2/Dux* TKO ESCs retain a MERVL-EGFP-positive population, our data do not establish bona fide totipotency or extraembryonic lineage potential. Future blastocyst injection and chimera formation assays will be required to determine the developmental potential of these cells.

## Methods

**Table:** Reagents and tools table

Reagent/resource	Reference or source	Identifier or catalog number
**Experimental models**		
E14tg2a ESCs	RIKEN Cell Bank	AES0135
E14tg2a MERVL::TurboGFP ESCs	This study/ Addgene reporter #69072	n.a.
*Dux* KO ESCs	This study	n.a.
*Ctbp1/2* DKO ESCs	This study	n.a.
*Ctbp1/2/Dux* TKO ESCs	This study	n.a.
*Pramel7* KO ESCs	This study	n.a.
PRAMEL7-overexpressing ESCs (P7OE)	This study	n.a.
P7OE/Dux KO ESCs	This study	n.a.
Ctbp1/2 DKO ESCs carrying doxycycline-inducible CtBP2	This study	n.a.
Doxycycline-inducible Dux ESCs	This study	n.a.
*Dnmt1* KO ESCs	Okano et al, 1999	n.a.
**Recombinant DNA**		
pX330-U6-Chimeric_BB-CBh-hSpCas9	Addgene (Cong et al, 2013)	Addgene plasmid #42230
MERVL::TurboGFP reporter plasmid	Addgene (Ishiuchi et al, 2015)	Addgene plasmid #69072
pGL4.23 luciferase reporter vector	Promega	E8411
Pramel7 enhancer luciferase reporter	This study	n.a.
Gata1 enhancer luciferase reporter	This study	n.a.
MERVL-LTR luciferase reporter	This study	n.a.
DUXmut-MERVL-LTR luciferase reporter	This study	n.a.
Zscan4c proximal enhancer reporter	This study	n.a.
Zscan4c MERVL-containing reporter	This study	n.a.
Naalad2 MERVL-containing reporter	This study	n.a.
FLAG-PRAMEL7 expression vector	This study	n.a.
Doxycycline-inducible CtBP2 transgene vector	This study	n.a.
Doxycycline-inducible Dux transgene vector	This study	n.a.
**Oligonucleotides and sequence-based reagents**		
**CRISPR**		
Ctbp1_up	Thermo Fisher Scientific	F: CACCGTCCGGGGTACGCGCAGGGG R: AAACCCCCTGCGCGTACCCCGGAC
Ctbp1_down	Thermo Fisher Scientific	F: CACCGTGCCCGCTCGCGACCCGCGC R: AAACGCGCGGGTCGCGAGCGGGCAC
Ctbp2_up	Thermo Fisher Scientific	F: CACCGATCCGCCCCCAGATCATGAA R: AAACTTCATGATCTGGGGGCGGATC
Ctbp2_down	Thermo Fisher Scientific	F: CACCGATCAAGGCTGCTGGCGAGCT R: AAACAGCTCGCCAGCAGCCTTGATC
Dux_up	Thermo Fisher Scientific	F: CACCGAAGGCACACAGCCGCTTGCT R: AAACAGCAAGCGGCTGTGTGCCTTC
Dux_down	Thermo Fisher Scientific	F: CACCGACTTTCCCCACTAGTGGCT R: AAACAGCCACTAGTGGGGAAAGTC
Pramel7_up	Thermo Fisher Scientific	F: CACCGTTTGCCTAAGAAGCAAATAG R: AAACCTATTTGCTTCTTAGGCAAAC
Pramel7_down	Thermo Fisher Scientific	F: CACCGCTCATGCTAGTACGCACCTT R: AAACAAGGTGCGTACTAGCATGAGC
Dnmt1_up	Thermo Fisher Scientific	F: CACCGTAATGTGAACCGGTTCACAG R: AAACCTGTGAACCGGTTCACATTAC
Dnmt1_down	Thermo Fisher Scientific	F: CACCGCGAGCAACAAGGCGCGTCA R: AAACTGACGCGCCTTGTTGCTCGC
**RT-qPCR primers**		
Gapdh	Thermo Fisher Scientific	F: ATGAATACGGCTACAGCAACAGG R: CTCTTGCTCAGTGTCCTTGCTG
Dux	Thermo Fisher Scientific	F: TGGATTGGGGTAGAAATCCTG R: GGATTCTCTGCAGAAGGTTGG
Zscan4c	Thermo Fisher Scientific	F: GAGATTCATGGAGAGTCTGACTGATGAGTG R: GCTGTTGTTTCAAAAGCTTGATGACTTC
Pramel7	Thermo Fisher Scientific	F: AATGTGCAATGGGGATATTCTG R: GATTTGGCTTGGCATACATTG
Obox4	Thermo Fisher Scientific	F: GTGTTCAGTTCTCCTCCATGCAGC R: AAGGTTGACTCCGGGTCTGAGAAC
Pramel16	Thermo Fisher Scientific	F: TTGTGCCTCTTGGGATTTTC R: AGGGATAAAAGCGCTGACCT
Gata1	Thermo Fisher Scientific	F: AGTCCTTTCTTCTCTCCCAC R: CTCCACAGTTCACACACTCTC
Klf17	Thermo Fisher Scientific	F: TAGGTCCCTGGGATTGAACTC R: CAGGCAAATACAGGAAAGCTG
Mervl	Thermo Fisher Scientific	F: CTCTACCACTTGGACCATATGAC R: GAGGCTCCAAACAGCATCTCTA
Zfp352	Thermo Fisher Scientific	F: TTCCGCCTATGTGATCTCAAC R: GCATGTTTCTTCTCCAGATGC
Gpx2	Thermo Fisher Scientific	F: TAGGTTCTGGGCCTTCACAG R: TGCCACACCTACACTTTATTGG
Zfp980	Thermo Fisher Scientific	F: GCCTACAGATATGAAGTGTGCTTTG R: GAGGAAGGAGATGTTGACAGAGC
Pou3f1	Thermo Fisher Scientific	F: TCCCTCCCCATCCTAAAGGG R: GAAGGGGGAAGCTCCAAACA
Obox3	Thermo Fisher Scientific	F: GGGTGGCTGTGGATTCATGC R: TCCTGTTGCATGTAGTGACCTC
Obox6	Thermo Fisher Scientific	F: CCCTTGACTTATCCAGGCCT R: CCACTACATAAACCAGTGACATG
Obox8	Thermo Fisher Scientific	F: CACATCAGACTCAGCTCTAGAG R: ATATGGATCTTCAAAGCTGCATC
**ChIP–qPCR primers**		
Gata1	Thermo Fisher Scientific	F: CCGACTTTCGTGAGGTAGTTTGC R: TTCTGGCCTTGGTACTAGCATCTC
Pramel7	Thermo Fisher Scientific	F: GTGTAACTGAAGGTGACCCTGAAC R: AGTCTAAAGGTTCAACAGCCACAG
**Genotyping primers**		
Dux_up	Thermo Fisher Scientific	F: GAGCGTTTTCCAAGTTCCGT R: TGCAAGAAACCTGACGAACA
Dux_down	Thermo Fisher Scientific	F: TGAACAGTCTCCATGCTCTGA R: CTGCTGCTGGGATGCTATTT
**Bisulfite sequencing primers for the Duxf3 locus**		
Dux	Thermo Fisher Scientific	F: GTTAGTTATTTGGTTAGGTTTA R: AATTCTATTTCTTTCTCTTCCA
**Antibodies**		
Anti-GFP antibody	Nacalai Tesque	04404-84
Anti-ZSCAN4 antibody	Merck Millipore	AB4340
Anti-MuERVL-Gag antibody	Epigentek	A-2801-050
Anti-OCT4 antibody	Santa Cruz Biotechnology	sc-8628
Anti-CtBP2 antibody	BD Biosciences	612044
Anti-UHRF1 antibody	MBL	D289-3
Anti-TUBULIN antibody	Sigma-Aldrich	T5168
Anti-GATA1 antibody	Proteintech	10917-2-AP
Anti-FLAG antibody	Sigma-Aldrich	F1804
Anti-CtBP1	BD Biosciences	612042
Anti-Rat IgG DyLight 488 antibody	Rockland	612-141-120
Anti-Rabbit IgG DyLight 549 antibody	KPL	042-04-15-06
Anti-Mouse IgG DyLight 550 antibody	Abcam	ab96876
Anti-Goat IgG DyLight 549 antibody	Rockland	605-742-002
HRP-conjugated anti-rabbit IgG secondary antibody	Santa Cruz	SC-2030
HRP-conjugated anti-mouse IgG secondary antibody	MBL	330
**Chemicals, peptides, and recombinant proteins**		
Glasgow minimum essential medium (GMEM)	Wako	078-05525
Fetal calf serum (FCS)	Invitrogen	n.a.
Non-essential amino acids	Wako	139-15651
Monothioglycerol solution	Wako	195-15791
Leukemia inhibitory factor (LIF)	In-house preparation	n.a.
PD0325901	Wako	162-25291
CHIR99021	Sigma-Aldrich	SML1046
Doxycycline	Wako	049-031121
Paraformaldehyde	Wako	162-16065
Triton X-100	Nacalai Tesque	12967-32
DAPI solution	DOJINDO	340-07971
HilyMax	DOJINDO	H357
2-mercaptoethanol	Wako	133-14571
Immunostar LD	Wako	290-69904
GSK-3484862	Wako	HY-135146
DMSO	Wako	047-29353
**Critical commercial assays and kits**		
Collibri Stranded RNA Library Prep Kit for Illumina Systems	Thermo Fisher Scientific	A39003096
Dual-Luciferase Reporter Assay System	Promega	E1910
Premium RRBS Kit V2	Diagenode	C02030036
**Instruments**		
Cell sorter	Sony Biotechnology	SH800
Fluorescence microscope	Leica Microsystems	SP8
Time-lapse microscope	Thermo Fisher Scientific	EVOS M7000
Real-time PCR system	Roche	LightCycler 96
Western blot imaging system	ATTO	WSE-6170 LuminoGraph I CMOS
Illumina HiSeq X Ten system	Illumina	HiSeq X Ten
Sequencer for bisulfite sequencing	Applied Biosystems	3500 Genetic Analyzer
Cell counter	Thermo Fisher Scientific	Countess II FL
**Software and algorithms**		
HISAT2	GitHub	https://anaconda.org/bioconda/hisat2
Rsubread	Liao et al, 2019	https://anaconda.org/channels/bioconda/packages/bioconductor-rsubread/overview
Trim galore	GitHub	https://github.com/FelixKrueger/TrimGalore
iDEP webserver	Ge et al, 2018	https://bioinformatics.sdstate.edu/idep/
GREAT	McLean et al, 2010	http://great.stanford.edu/public/html/splash.php
IGV	Robinson et al, 2011	https://igv.org/
BioVenn	Hulsen et al, 2008	https://www.biovenn.nl/
GSEA	Subramanian et al, 2005	https://www.gsea-msigdb.org/gsea/index.jsp
MEGA software	MEGA	https://www.megasoftware.net/
samtools	Li et al, 2009	https://anaconda.org/channels/bioconda/packages/samtools/overview
ggplot2	CRAN	https://cran.r-project.org/web/packages/ggplot2/index.html
ImageJ/Fiji	NIH/Fiji	https://imagej.net/software/fiji/
TrackMate	NIH/Fiji	https://imagej.net/plugins/trackmate/
Bismark	GitHub	https://github.com/FelixKrueger/Bismark
methylKit	Akalin et al, 2012	https://bioconductor.org/packages/release//bioc/html/methylKit.html
**Public datasets and genome resources**		
Mus musculus genome assembly mm10	UCSC Genome Browser	https://hgdownload.soe.ucsc.edu/goldenPath/mm10/bigZips/
UCSC mm10 KnownGenes GTF	UCSC Genome Browser	https://hgdownload.soe.ucsc.edu/goldenpath/mm10/bigZips/genes/
2CLC-enriched gene set/GSE33923	Macfarlan et al, 2012	GSE33923
Early mouse embryo RNA-seq dataset	Zhang et al, 2016	GSE71434
Dux KO embryo RNA-seq dataset	Guo et al, 2019	GSE134832
CtBP2 ChIP-seq dataset	Kim et al, 2015	E-MTAB-2002
DUX ChIP-seq dataset	Hendrickson et al, 2017	GSE85632
H3K27Ac ChIP-seq dataset	Zhang et al, 2020	GSE141525
RNA-seq data and RRBS data	This study	GSE253944

### Cell culture

E14tg2a ESCs were obtained from the RIKEN Cell Bank and cultured in Glasgow minimum essential medium (GMEM; Wako) supplemented with 10% fetal calf serum (FCS; Invitrogen), 1 mM L-glutamine (Wako), non-essential amino acids (Wako), 500 μM monothioglycerol (Wako), and 1000 U/ml leukemia inhibitory factor (LIF; Wako). ESCs were maintained on gelatin-coated dishes without feeder cells.

### Generation of knockout and overexpression ESC lines

Guide RNAs targeting *Ctbp1*, *Ctbp2*, *Dux*, and *Pramel7* were cloned into pX330-U6-Chimeric_BB-CBh-hSpCas9 (Addgene plasmid #42230; deposited by (Cong et al, 2013)). The guide RNA sequences are listed in the Reagents & Tools Table. For genome editing, pX330-based CRISPR-Cas9 plasmids were co-transfected with pCAG-EGFP into E14tg2a ESCs carrying the MERVL-EGFP reporter. GFP-positive cells were isolated using a cell sorter (SH800; Sony Biotechnology), plated at clonal density, and expanded. Genomic deletions were confirmed by PCR and Sanger sequencing, and representative deletion alleles are shown in Appendix Fig. S1A–C.

For overexpression experiments, *Pramel7* was introduced into WT and *Dux* KO ESCs using an expression vector carrying FLAG-tagged PRAMEL7. Stable clones were established after drug selection. PRAMEL7 expression was confirmed by western blotting using an anti-FLAG antibody. Doxycycline-inducible CtBP2 and DUX ESC lines were generated by introducing inducible expression constructs into ESCs. CtBP2 or DUX expression was induced by doxycycline treatment as indicated in each experiment.

### Flow cytometry and cell sorting

MERVL-EGFP reporter activity was analyzed by flow cytometry. Cells were dissociated into single-cell suspensions, filtered through a cell strainer, and analyzed using a flow cytometer. The MERVL-EGFP-positive population was subdivided into GFP+ and GFP + ++ fractions using fixed fluorescence intensity gates that were applied consistently across WT, *Dux* KO, *Ctbp1/2* DKO, and *Ctbp1/2/Dux* TKO ESCs. For re-culture experiments, MERVL-EGFP-positive cells were sorted from *Ctbp1/2* DKO and *Ctbp1/2/Dux* TKO ESCs and re-plated under standard ESC culture conditions. GFP expression was re-analyzed 8 days after sorting.

### Growth curve and alkaline phosphatase staining

For growth curve analysis, equal numbers of WT, *Dux* KO, *Ctbp1/2* DKO, and *Ctbp1/2/Dux* TKO ESCs were seeded under standard ESC culture conditions. Cells were counted at the indicated time points, and relative cell numbers were calculated relative to the initial seeding number. For alkaline phosphatase staining, ESCs were seeded at a clonal density and cultured under standard ESC culture conditions. AP-positive colonies were counted from independent biological replicates.

### Time-lapse imaging and lineage tracking

Time-lapse imaging of MERVL-EGFP reporter ESCs was performed using an EVOS M7000 imaging system. Cells were imaged under standard ESC culture conditions at 30-min intervals for up to 72 h. Bright-field and GFP images were acquired at each time point. GFP-positive cells were detected and tracked using TrackMate in Fiji/ImageJ. TrackMate outputs were used to determine the trajectories of individual cells and founder-derived lineages. Tracking results were manually inspected and corrected where necessary, especially for cell division events. The onset of GFP expression was defined as the first frame in which a tracked cell became detectably GFP-positive. GFP fluorescence intensity was measured for each tracked cell over time using the GFP channel. For each founder-derived lineage, we calculated the maximal GFP intensity, maximal fluorescence change per hour, and the total number of cell divisions after GFP onset. Cells that collapsed, divided, or reverted to a GFP-negative state were annotated based on the corresponding time-lapse images.

### RT-qPCR and RNA-Seq

Total RNA was purified using a PureLink™ RNA Mini Kit (Ambion). cDNA was synthesized using ReverTra Ace® qPCR RT Master Mix (Toyobo). RT-qPCR was performed using FastStart SYBR Green Master Mix (Roche) and gene-specific primers listed in the Reagents and Tools Table. *Gapdh* was used as an internal control. ΔCt values were calculated as Ct(*Gapdh*)−Ct(target gene), unless otherwise indicated. Relative expression values were calculated with WT or the indicated control condition set to 1.

RNA-seq libraries were generated using the Collibri™ Stranded RNA Library Prep Kit for Illumina™ Systems (Thermo Fisher Scientific) according to the manufacturer’s instructions and sequenced on an Illumina HiSeq X Ten platform to generate 150-bp paired-end reads.

### Data analysis of RNA-Seq and public ChIP-seq data

Raw gene counts were calculated using featureCounts from the Rsubread package with the UCSC mm10 KnownGenes GTF annotation. Normalized count data, principal component analysis, and K-means clustering analysis were exported from the interactive Expression and Pathway analysis (iDEP) webserver using count data or normalized expression data from early mouse embryos (Ge et al, 2018). Differentially expressed genes (DEGs) were selected using a cut-off at a false discovery rate (FDR) ≦ 0.1 and a ≧ twofold change in expression. Gene set enrichment analysis (GSEA) (Subramanian et al, 2005) was used to determine whether upregulated genes by *Ctbp1/2* knockouts were enriched for the upregulated genes in 2-cell embryos or 2CLCs as described previously. Venn diagrams showing overlapping genes were constructed by BioVenn, a web application (Hulsen et al, 2008). For 2CLC-transition signature analysis, gene sets associated with sequential transitions from ESCs to ZSCAN4-low cells, ZSCAN4-low to ZSCAN4-mid cells, ZSCAN4-mid to ZSCAN4-high cells, and ZSCAN4-high cells to MERVL-EGFP-positive cells were obtained from Rodriguez-Terrones et al Signature scores were calculated from the mean expression of genes included in each gene set after gene-wise normalization.

Public ChIP-seq data for CtBP2 were re-analyzed using GREAT (Genomic Regions Enrichment of Annotations tool) with the “two nearest genes” setting, which assigns each peak to the two closest transcription start sites (TSSs) within 1 Mb (McLean et al, 2010). Peak visualization of ChIP-seq peaks for CtBP2, H3K27Ac, and DUX was performed using Integrative Genomics Viewer (IGV) (Hendrickson et al, 2017; Kim et al, 2015; McLean et al, 2010; Zhang et al, 2020).

### Immunofluorescence analysis and image quantification

Cells were fixed with 4% paraformaldehyde at 4 °C for 20 min and permeabilized with PBS containing 0.5% Triton X-100 at 4 °C for 5 min. Permeabilized cells were incubated with primary antibodies against GFP, ZSCAN4, MERVL-GAG, OCT4, GATA1, or UHRF1, followed by fluorophore-conjugated secondary antibodies. Nuclei were counterstained with 1 μg/ml DAPI solution (Dojindo).

For quantification of UHRF1 intensity, nuclei were segmented using DAPI staining, and ZSCAN4-positive and ZSCAN4-negative cells were classified based on ZSCAN4 signal intensity. Nuclear UHRF1 intensity was measured for each cell and normalized to the mean intensity of the control population.

Primary antibodies used were anti-GFP (Nacalai Tesque, 04404-84, 1:500), anti-ZSCAN4 (Merck Millipore, AB4340, 1:1000), anti-MERVL-GAG (Epigentek, A-2801-050, 1:250), anti-OCT4 (Santa Cruz, sc-8628, 1:500), anti-CtBP2 (BD Biosciences, 612044, 1:500), anti-GATA1 (Proteintech, 10917-2-AP, 1:100) and anti-UHRF1 (MBL, D289-3, 1:200). Secondary antibodies included anti-rat IgG DyLight™ 488 (Rockland, 612-141-120, 1:500), anti-rabbit IgG DyLight™ 549 (KPL, 042-04-15-06, 1:500), anti-mouse IgG DyLight® 550 (Abcam, ab96876, 1:500) and anti-goat IgG DyLight™ 549 (Rockland, 605-742-002, 1:500).

### Luciferase assay

Candidate regulatory regions, including MERVL-LTR fragments, DUX-bound regions near *Zscan4c* and *Naalad2*, and distal regulatory regions of *Pramel7* and *Gata1*, were cloned into the pGL4.23 luciferase reporter vector. Mutations were introduced into the DUX-binding motif of the MERVL-LTR reporter where indicated. WT, *Dux* KO, *Ctbp1/2* DKO, and *Ctbp1/2/Dux* TKO ESCs were transfected with reporter constructs together with the indicated expression vectors using HilyMax (Dojindo). Cells were harvested 36 h after transfection, and luciferase activity was measured using the Dual-Luciferase Reporter Assay System (Promega). Relative luciferase activity was normalized to the internal control reporter.

### ChIP–qPCR

ChIP–qPCR was performed with modifications based on a previously described protocol (Naitou et al, 2022). Doxycycline-inducible FLAG-CtBP2 ESCs were crosslinked with 1% formaldehyde for 10 min, followed by quenching with 125 mM glycine. Fixed cells were sequentially treated with LB1, LB2, and LB3 buffers, and nuclei were lysed in SDS buffer. Chromatin was fragmented by sonication, and protein-DNA complexes were immunoprecipitated at 4 °C overnight using an anti-FLAG antibody (Sigma, F3165) bound to Dynabeads Protein G. Immunoprecipitates were washed, eluted, and reverse-crosslinked at 65 °C overnight. After RNase A and Proteinase K treatment, ChIP DNA and input DNA were purified and analyzed by qPCR using primers targeting the *Pramel7* and *Gata1* regulatory regions. ChIP enrichment was calculated as percent input. Primer sequences are listed in the Reagents and Tools Table.

### Western blotting analysis

Cells were lysed in SDS sample buffer and denatured by heating in the presence of 2-mercaptoethanol. Proteins were separated by SDS-PAGE and transferred to PVDF membranes. Membranes were incubated with primary antibodies against CtBP1, CtBP2, FLAG, UHRF1, or α-TUBULIN, followed by HRP-conjugated secondary antibodies. Signals were detected using Immunostar LD (Wako).

Primary antibodies used were anti-UHRF1 (MBL, D289-3, 1:1000), anti-α-TUBULIN (Sigma, T5168, 1:5000), anti-CtBP1 (BD Bioscience, 612042, 1:1000), anti-CtBP2 (BD Bioscience, 612044, 1:1000), and anti-FLAG (Sigma, F1804, 1:500).

### RRBS and bisulfite sequencing

Reduced representation bisulfite sequencing (RRBS) was performed using genomic DNA from WT and PRAMEL7-overexpressing ESCs. RRBS libraries were prepared using the Premium RRBS kit V2 (Hologic Diagenode, Cat# C02030036) according to the manufacturer’s instructions. Libraries were sequenced to generate 150-bp paired-end reads. RRBS reads were adapter- and quality-trimmed using Trim Galore with the RRBS option. Trimmed reads were aligned to the mouse genome assembly mm10 using Bismark. CpG methylation calls were extracted using Bismark, and methylation summary statistics were calculated using methylKit. CpG methylation levels were calculated for individual CpG sites and summarized across the indicated genomic regions. For promoter-proximal methylation analysis, CpG methylation levels within TSS ± 1 kb were compared between WT and P7OE ESCs.

For targeted bisulfite sequencing, genomic DNA from WT and *Ctbp1/2* DKO ESCs was bisulfite-converted, and the *Duxf3* locus was amplified by PCR using bisulfite-specific primers. PCR products were cloned and sequenced. Filled and open circles represent methylated and unmethylated CpGs, respectively.

### Phylogenetic tree inference

Multiple-sequence alignment was performed using the ClustalW algorithm in MEGA software, version 11, withdefault parameters. Molecular phylogenetic trees were inferred using the maximum-likelihood method implementedin MEGA11 with default parameters (Tamura et al, 2021).

## Supplementary information

Appendix

Peer Review File

Dataset EV1

Movie EV1

Movie EV2

Movie EV3

Movie EV4

Source data Fig. 1

Source data Fig. 2

Source data Fig. 3

Source data Fig. 4

Source data Fig. 5

Source data Fig. 6

Source data Fig. 7

Source data Fig. 8

Appendix_Figure_S1_source_data

Appendix_Figure_S2_source_data

Appendix_Figure_S3_source_data

## Data Availability

The RNA-seq and RRBS datasets generated in this study have been deposited in the Gene Expression Omnibus (GEO) under accession number GSE253944. The source data of this paper are collected in the following database record: biostudies:S-SCDT-10_1038-S44319-026-00881-7.
