# Supplementary material for: CtBP1/2 restrict DUX-dependent and independent 2C-like programs in mouse embryonic stem cells
Source: EMBO Rep. 2026 Aug 3;27(18):5317–40. doi: 10.1038/s44319-026-00881-7 (PMC13601577; doi:10.1038/s44319-026-00881-7)
Supplement: Supplementary file 1 [file 44319_2026_881_MOESM1_ESM.pdf]

Appendix for CtBP1/2 restrict DUX-dependent and independent 2C-like programs in mouse embryonic stem cells

Table of Contents

Appendix Figure S1. page 2

Appendix Figure S2. page 3

Appendix Figure S3. page 4

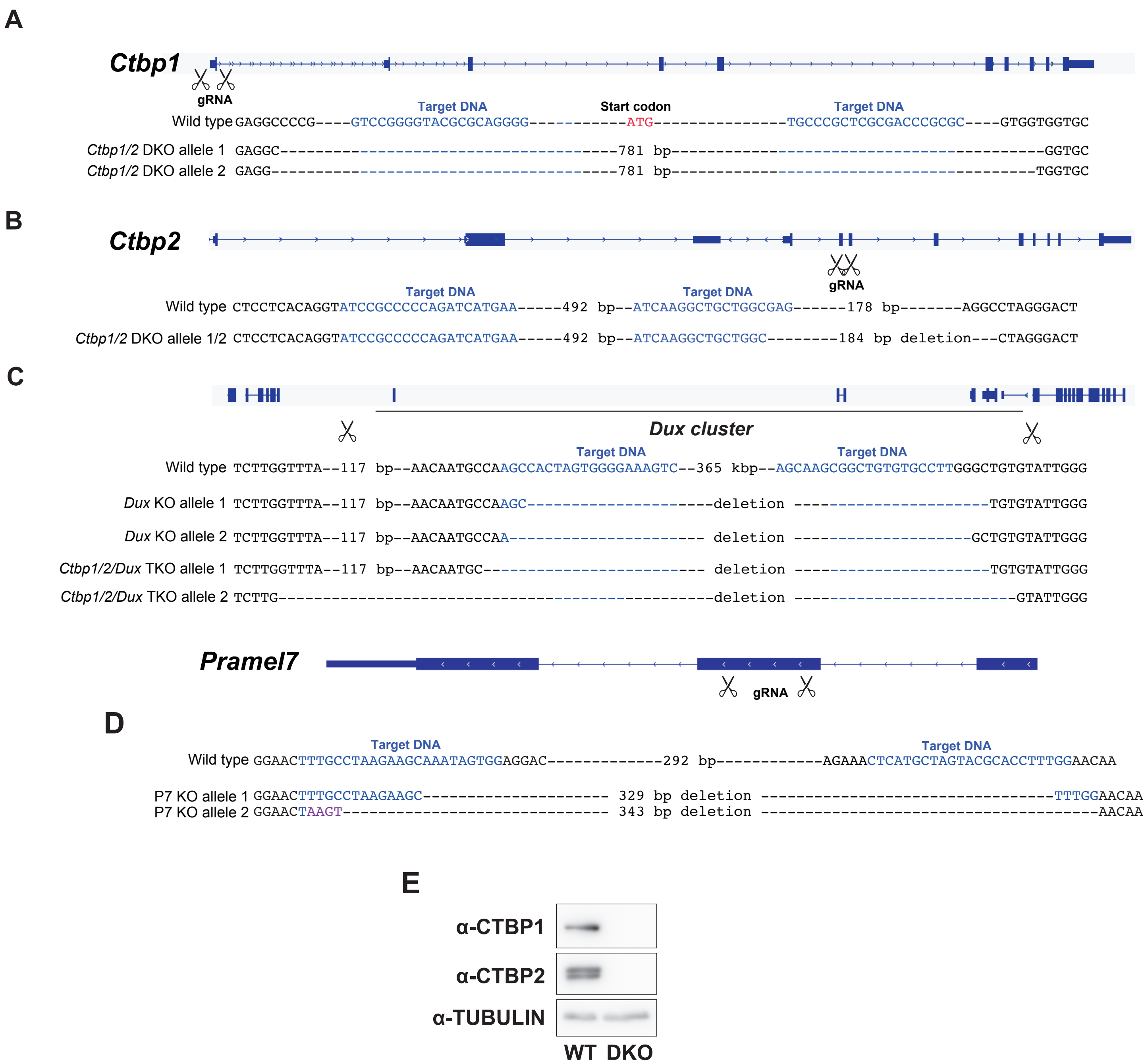

Appendix Figure S1. Generation and validation of *Ctbp1/2* DKO and *Ctbp1/2/Dux* TKO ESCs. (A–D) Representative genome-edited alleles of *Ctbp1* (A), *Ctbp2* (B), the *Dux* cluster (C) and *Pramel7* (D) in the indicated KO ESC lines. The gRNA target sites and deleted regions are shown. (E) Western blot analysis of CTBP1 and CTBP2 protein levels in WT and *Ctbp1/2* DKO ESCs. α-TUBULIN was used as a loading control.

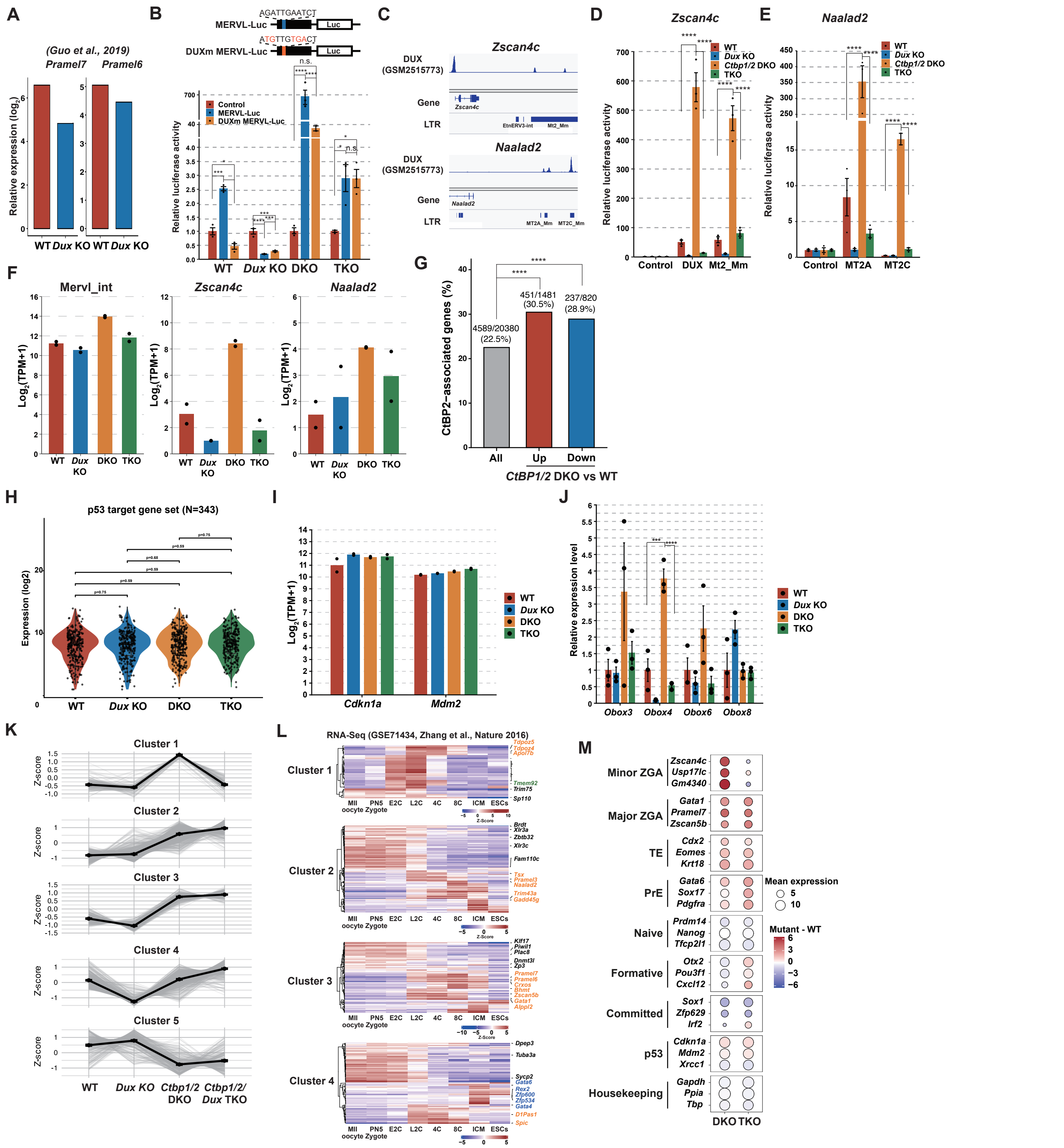

Appendix Figure S2. Additional analyses supporting DUX-dependent and DUX-independent transcriptional responses after *Ctbp1/2* loss.

(A) Expression levels of *Pramel7* and *Pramel6* in WT and *Dux* KO early embryos based on re-analysis of the published RNA-seq dataset from Guo et al. (2019). (B) Luciferase reporter assay using MERVL-Luc and DUX-binding motif-mutated MERVL-Luc reporters in WT, *Dux* KO, *Ctbp1/2* DKO and *Ctbp1/2/Dux* TKO ESCs. Bars indicate mean ± SEM from three biological replicates; individual data points are shown. Statistical significance was assessed within each genotype by one-way ANOVA followed by Tukey's HSD multiple-comparison test. (C) Genome browser views of public DUX ChIP-seq signals (GSM2515773) at the *Zscan4c* and *Naalad2* loci, with nearby genes and LTR elements indicated. (D, E) Luciferase reporter assays using candidate regulatory elements near *Zscan4c* (D) and *Naalad2* (E) in WT, *Dux* KO, *Ctbp1/2* DKO and *Ctbp1/2/Dux* TKO ESCs. Bars indicate mean ± SEM from biological triplicates; individual data points are shown. Statistical significance was assessed within each vector condition by one-way ANOVA followed by Tukey's HSD multiple-comparison test. (F) RNA-seq expression levels of Mervl_int, *Zscan4c* and *Naalad2* in WT, *Dux* KO, *Ctbp1/2* DKO and *Ctbp1/2/Dux* TKO ESCs. Values are shown as log2(TPM + 1). Dots indicate individual biological replicates; n = 2 biological replicates. (G) Publicly available CtBP2 ChIP-seq peaks were assigned to nearby genes using GREAT with the "Two nearest genes" rule. Bars show the percentage of CtBP2-associated genes among all RNA-seq background genes, *Ctbp1/2* DKO-upregulated genes and *Ctbp1/2* DKO-downregulated genes. Numbers above bars indicate CtBP2-associated genes/total genes and percentages. The numbers of genes analyzed were n = 20,380 RNA-seq background genes, n = 1,481 *Ctbp1/2* DKO-upregulated genes and n = 820 *Ctbp1/2* DKO -downregulated genes. Statistical significance was assessed by one-sided Fisher's exact test followed by Benjamini–Hochberg correction for multiple testing. (H) Expression distribution of a curated p53 target gene set in WT, *Dux* KO, *Ctbp1/2* DKO and *Ctbp1/2/Dux* TKO ESCs. The p53 target gene set contained 343 genes, of which 275 genes were detected in the RNA-seq dataset and used for visualization and statistical analysis. Each dot indicates one detected p53 target gene. Statistical significance was assessed by two-sided Wilcoxon rank-sum tests followed by Benjamini–Hochberg correction for multiple testing. (I) RNA-seq expression levels of the canonical p53 target genes *Cdkn1a* and *Mdm2* in WT, *Dux* KO, *Ctbp1/2* DKO and *Ctbp1/2/Dux* TKO ESCs. Values are shown as log2(TPM + 1). Dots indicate individual biological replicates; n = 2 biological replicates. (J) RT-qPCR analysis of selected *Obox* genes in WT, *Dux* KO, *Ctbp1/2* DKO and *Ctbp1/2/Dux* TKO ESCs. Bars indicate mean ± SEM from three biological replicates; individual data points are shown. Statistical significance was assessed separately for each gene by one-way ANOVA followed by Tukey's HSD multiple-comparison test. (K) K-means clustering of transcriptional changes across WT, *Dux* KO, *Ctbp1/2* DKO and *Ctbp1/2/Dux* TKO ESCs. Gray lines indicate individual genes, and black lines indicate the mean z-score for each cluster. (L) Heatmaps showing the expression dynamics of genes in Clusters 1–4 during early mouse embryogenesis using the published RNA-seq dataset from Zhang et al. (2016). Genes with an average FPKM ≤ 1 were excluded from the analysis. (M) Dot plot summarizing the expression of representative genes from selected gene-signature categories in *Ctbp1/2* DKO and *Ctbp1/2/Dux* TKO ESCs relative to WT ESCs. Dot size indicates mean expression, and color indicates expression changes relative to WT. For panels with statistical annotations, significance labels are defined as follows: **P* < 0.05; ***P* < 0.01; ****P* < 0.001; *****P* < 0.0001; n.s., not significant. Exact adjusted *P* values and complete statistical results are provided in the accompanying README and Source Data files.

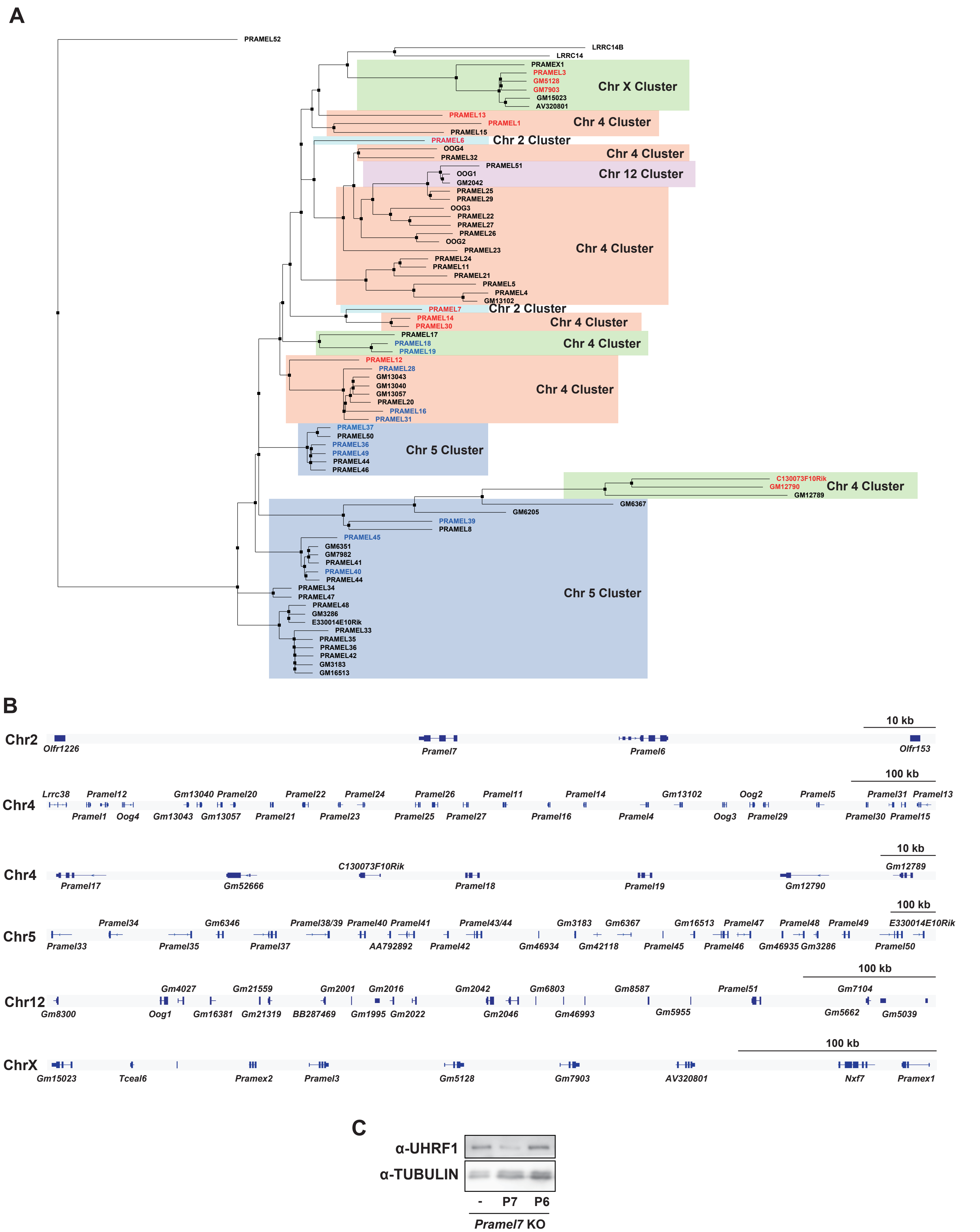

Appendix Figure S3. Molecular phylogeny and genomic organization of PRAME family genes.
(A) Maximum-likelihood phylogenetic tree constructed using amino acid sequences of mouse PRAME family proteins, with LRRC14 used as an outgroup. Red labels indicate genes upregulated by *Ctbp1/2* DKO in the absence of DUX, whereas blue labels indicate genes upregulated by *Ctbp1/2* DKO in a DUX-dependent manner. (B) Genomic organization and synteny of PRAME family genes on chromosomes 2, 4, 5, 12 and X. (C) Western blot analysis of UHRF1 protein levels in *Pramel7* KO ESCs expressing PRAMEL7 (P7) or PRAMEL6 (P6). α-TUBULIN was used as a loading control.
