# Supplementary material for: CtBP1/2 restrict DUX-dependent and independent 2C-like programs in mouse embryonic stem cells
Source: EMBO Rep. 2026 Aug 3;27(18):5317–40. doi: 10.1038/s44319-026-00881-7 (PMC13601577; doi:10.1038/s44319-026-00881-7)
Supplement: Supplementary file 2 [file 44319_2026_881_MOESM2_ESM.pdf]

EMBO reports

CtBP1/2 restrict DUX-dependent and independent 2C-like programs in mouse embryonic stem cells

Kazuma Yoshioka, Maki Ichisakino, Kota Sugiyama, Nao Hayakawa, Selma Abadi, Miyu Marutani, Ryo Masuda, Kyo Takahashi, Heekyoung Yoon, and Yoshiyuki Seki

Corresponding author(s): Yoshiyuki Seki (yseki@kwansei.ac.jp)

Review Timeline:

Submission Date:	11th Nov 25
Editorial Decision:	7th Jan 26
Revision Received:	30th Apr 26
Editorial Decision:	19th Jun 26
Revision Received:	6th Jul 26
Accepted:	9th Jul 26

Editor: Achim Breiling

Transaction Report:

(Note: With the exception of the correction of typographical or spelling errors that could be a source of ambiguity, letters and reports are not edited. Depending on transfer agreements, referee reports obtained elsewhere may or may not be included in this compilation. Referee reports are anonymous unless the Referee chooses to sign their reports.)

Dear Dr. Seki,

Thank you for the submission of your manuscript to EMBO reports. I have now received reports from the three referees that were asked to evaluate your study, which can be found at the end of this email. As you will see, the referees have several comments, concerns, and suggestions, indicating that a major revision of the manuscript is necessary to allow publication of the study in EMBO reports. As the reports are below, and all points need to be addressed, I will not detail them here.

Given the constructive referee comments, I would thus like to invite you to revise your manuscript with the understanding that the concerns of the referees must be addressed in the revised manuscript and in a detailed point-by-point response. Acceptance of your manuscript will depend on a positive outcome of a second round of review. It is EMBO reports policy to allow a single round of revision only and acceptance of the manuscript will therefore depend on the completeness of your responses included in the next, final version of the manuscript.

Revised manuscripts should be submitted within three months of a request for revision. Please contact me to discuss the revision (also by video chat) if you have questions or comments regarding the revision, or should you need additional time.

When submitting your revised manuscript, please also carefully review the instructions that follow below.

PLEASE NOTE THAT upon resubmission revised manuscripts are subjected to an initial quality control prior to exposition to re-review. Upon failure in the initial quality control, the manuscripts are sent back to the authors, which may lead to delays. Frequent reasons for such a failure are the lack of the data availability section (please see below) and the presence of statistics based on n=2 (the authors are then asked to present scatter plots or provide more data points).

When submitting your revised manuscript, we will require:

1) a .docx formatted version of the final manuscript text (including legends for main figures, EV figures and tables), but without the figures included. Figure legends should be compiled at the end of the manuscript text.

2) individual production quality figure files as .eps, .tif, .jpg (one file per figure), of main figures and EV figures. Please upload these as separate, individual files upon re-submission.

The Expanded View format, which will be displayed in the main HTML of the paper in a collapsible format, has replaced the Supplementary information. You can submit up to 6 images as Expanded View. Please follow the nomenclature Figure EV1, Figure EV2 etc. The figure legend for these should be included in the main manuscript document file in a section called Expanded View Figure Legends after the main Figure Legends section. Additional Supplementary material should be supplied as a single pdf file labeled Appendix. The Appendix should have page numbers and needs to include a table of content on the first page (with page numbers) and legends for all content. Please follow the nomenclature Appendix Figure Sx, Appendix Table Sx etc. throughout the text, and also label the figures and tables according to this nomenclature.

For more details, please refer to our guide to authors:
https://link.springer.com/journal/44319/submission-guidelines#cms-Revised-submissions

Please consult our guide for figure preparation:
https://media.springernature.com/original/springer-cms/rest/v1/content/27825798/data/v1

3) a .docx formatted letter INCLUDING the reviewers' reports and your detailed point-by-point responses to their comments. As part of the EMBO Press transparent editorial process, the point-by-point response is part of the Review Process File (RPF), which will be published alongside your paper.

4) a complete author checklist, which you can download from our author guidelines (https://link.springer.com/journal/44319/submission-guidelines#cms-Revised-submissions). Please insert page numbers in the checklist to indicate where the requested information can be found in the manuscript. The completed author checklist will also be part of the RPF.

Please also follow our guidelines for the use of living organisms, and the respective reporting guidelines:
https://link.springer.com/partners/embo-press/editorial-policies#Animal%20and%20human%20studies

5) that primary datasets produced in this study (e.g. RNA-seq, ChIP-seq, structural and array data) are deposited in an appropriate public database. If no primary datasets have been deposited, please also state this in a dedicated section (e.g. 'No primary datasets have been generated and deposited'), see below.

See also: https://link.springer.com/partners/embo-press/editorial-policies#Data%20deposition

Please remember to provide a reviewer password if the datasets are not yet public.

The accession numbers and database should be listed in a formal "Data Availability" section that follows the model below. This is now mandatory (like the COI statement). Please note that the Data Availability Section is restricted to new primary data that are part of this study. This section is mandatory. As indicated above, if no primary datasets have been deposited, please state this in this section

Data availability

The datasets produced in this study are available in the following databases:

- RNA-Seq data: Gene Expression Omnibus GSE46843 (https://www.ncbi.nlm.nih.gov/geo/query/acc.cgi?acc=GSE46843)
- [data type]: [name of the resource] [accession number/identifier/doi] ([URL or identifiers.org/DATABASE:ACCESSION])

*** Note - All links should resolve to a page where the data can be accessed. ***

See also: https://link.springer.com/partners/embo-press/editorial-policies#Data%20availability%20statement

Moreover, I have these editorial requests:

6) We now request the publication of original source data with the aim of making primary data more accessible and transparent to the reader. You will receive a separate email with instructions for providing source data with your revised manuscript, including information how to upload and organize the files.

7) Our journal encourages inclusion of *data citations in the reference list* to directly cite datasets that were re-used and obtained from public databases. Data citations in the article text are distinct from normal bibliographical citations and should directly link to the database records from which the data can be accessed. In the main text, data citations are formatted as follows: "Data ref: Smith et al, 2001" or "Data ref: NCBI Sequence Read Archive PRJNA342805, 2017". In the Reference list, data citations must be labeled with "[DATASET]". A data reference must provide the database name, accession number/identifiers and a resolvable link to the landing page from which the data can be accessed at the end of the reference. Further instructions are available at: https://link.springer.com/journal/44319/submission-guidelines#cms-Reference-guidelines

8) Regarding data quantification and statistics, please make sure that the number "n" for how many independent experiments were performed, their nature (biological versus technical replicates), the bars and error bars (e.g. SEM, SD) and the test used to calculate p-values is indicated in the respective figure legends (also for EV and Appendix figures). Please also check that all the p-values are explained in the legend, and that these fit to those shown in the figure. Please provide statistical testing where applicable. Please avoid the phrase 'independent experiment' but clearly state if these were biological or technical replicates. Please also indicate (e.g. with n.s.) if testing was performed, but the differences are not significant. In case n=2, please show the data as separate datapoints without error bars and statistics. See also:
https://link.springer.com/journal/44319/submission-guidelines#cms-Figure-and-data-presentation

If n<5, please show single datapoints for diagrams.

9) Please add scale bars of similar style and thickness to all microscopic images, using clearly visible black or white bars (depending on the background). Please place these in the lower right corner of the images themselves. Please do not write on or near the bars in the image but define the size in the respective figure legend.

10) Please also note our reference format:
https://link.springer.com/journal/44319/submission-guidelines#cms-Reference-guidelines

11) We updated our journal's competing interests policy in January 2022 and request authors to consider both actual and perceived competing interests. Please review the policy https://www.embopress.org/competing-interests and update your competing interests if necessary. Please name this section 'Disclosure and Competing Interests Statement' and put it after the Acknowledgements section.
See: https://link.springer.com/partners/embo-press/editorial-policies#Competing%20interest%20disclosures

12) We now use CRediT to specify the contributions of each author in the journal submission system. CRediT replaces the author contribution section. Please use the free text box to provide more detailed descriptions and do NOT provide your final manuscript text file with an author contributions section. See also our guide to authors (section 'Author contributions'):
https://link.springer.com/journal/44319/submission-guidelines#cms-Revised-submissions

13) All Materials and Methods need to be described in the main text using our 'Structured Methods' format, which is required for all research articles. According to this format, the Methods section should include a Reagents and Tools Table (listing key

reagents, experimental models, software, and relevant equipment and including their sources and relevant identifiers), uploaded as separate file, and a Methods section in which we encourage the authors to describe their methods using a step-by-step protocol format with bullet points, to facilitate the adoption of the methodologies across labs. More information on how to adhere to this format as well as downloadable templates (.doc) for the Reagents and Tools Table can be found in our author guidelines (section 'Structured Methods'):

https://link.springer.com/journal/44319/submission-guidelines#cms-Manuscript-organisation-and-formatting

Please add all primer information directly to the Reagents and Tools table.

14) Please order the manuscript sections like this, using only these names:
Title page - Abstract (max. 175 words) - Keywords (max. 5) - Introduction - Results - Discussion - Methods - Data availability section - Acknowledgements (please include here all the funding information) - Disclosure and Competing Interests Statement - References - Figure legends - Expanded View Figure legends

15) Please make sure that all the funding information is also entered into the online submission system and that it is complete and similar to the one in the acknowledgement section of the manuscript text file.

We would also welcome the submission of cover suggestions, or motifs to be used by our Graphics Illustrator in designing a cover.

As part of the EMBO publication's Transparent Editorial Process, EMBO reports publishes online a Review Process File (RPF) to accompany accepted manuscripts. This File will be published in conjunction with your paper and will include the referee reports, your point-by-point response and all pertinent correspondence relating to the manuscript.

You are able to opt out of this by letting the editorial office know (emboreports@embo.org). If you do opt out, the Review Process File link will point to the following statement: "No Review Process File is available with this article, as the authors have chosen not to make the review process public in this case."

I look forward to seeing a revised form of your manuscript when it is ready.

Yours sincerely,

Achim Breiling
Senior Editor
EMBO Reports

Referee #1:

2C-like cells (2CLCs) emerged from mESCs partially resemble the transcription program of mouse 2C embryos. These cells provide a valuable model for understanding the 2C embryos and totipotency program in vitro. Yoshioka and colleagues sought to understand how the transcription program is regulated in 2CLCs. Through analyzing their previous Ctbp1/2 DKO mESCs, they found Ctbp1/2 may work as repressors of the 2C program in mESCs. The authors found a DUX independent pathway was activated in Ctbp1/2 DKO which the authors propose could be partially attributed to Pramel7.

Overall, this article presented valuable insights into the in vitro modeling of the 2C program of mouse early embryos. The Ctbp1/2 DKO mESCs provided a rich resource to study the nature of 2CLC and its transcription program. The most interesting result in the paper is the persistence of MERVL-reporter positive cells in the absence of Dux. However, its nature remains elusive. The current model proposed by the authors is interesting but alternative scenarios still exist, which would undermine the major message of the paper. This needs to be clarified before the paper is considered for publication. Please see below for my detailed comments.

1. The expansion of 2CLC population in Ctbp1/2 DKO mESCs, which is independent of Dux (Dux KO even increased the 2CLC population), is one of the most surprising results in this article. While 2C genes may include a large number of genes, those that are specifically expressed during minor ZGA (MERVL, Zscan4, Usp17l, Tdpoz4) are most characteristic ones. Given most typical 2C-marker genes (Zscan4, Usp17l, Tdpoz4) were still fully dependent on DUX, this persisting cell population may mainly represent the high activity of MERVL-reporter. Therefore, one alternative possibility is that Ctbp1/2 DKO causes upregulation of an activator of MERVL, which leads to the persisting 2C population even in the absence of DUX.
2. For the cluster 2-4 genes that are activated in CtBP1/2 ("Dux-independent 2C genes"), the fact that some are expressed in oocytes while others are expressed in embryos may also raise a possibility that they do not have specificity for embryo expression, but are general targets (direct or indirect) of CtBP1/2 that happens to overlap with genes expressed in oocytes and

embryos. It is probably not surprising that a general list of genes also include those that are expressed in oocytes or early embryos. As a comparison, cluster 1 is very specific for early 2C and late 2C embryos (Fig. 2H). This is especially important given Ctbp1/2 are general repressors involved in multiple cellular processes. A deep analysis should be performed to critically distinguish whether the up-regulation of Dux-independent 2C genes in Ctbp1/2 DKO cells is specific.
3. As an important test of these cells as "2C-like" cells, would Ctbp1/2/Dux TKO MERVL positive mESCs contribute to extraembryonic tissue? A minor question is do these cells also exhibit the "cycling-in-and-out" feature of 2CLCs? If the authors sort the MERVL positive cells and culture them, would they keep MERVL expression?
4. Given there are more than 2500 differential expressed genes in CtBP1/2 KO mESC, what's the phenotype of the KO cells? Do CtBP1/2-deficient mESCs proliferate normally? If the cells are stressed or close to losing pluripotency/viability, this may also confound the interpretation of the observed 2CLC increase, given p53 is known to activate Dux.
5. PRAMEL7 OE can activate MERVL-reporter but in Dux-dependent manner, which indicates that its function on classic 2C genes (minor ZGA genes) is still Dux-dependent. Given it is not the factor that can activate MERVL without DUX, the real key factor that enable this surprising result is still unknown.
6. Figures 2A and 2B show that 24.95% of CtBP1/2/Dux TKO cells are MERVL-GFP-positive, which is even higher than in CtBP1/2 DKO cells (14.09%). However, the manuscript states: "While the strong GFP-positive population was markedly reduced in TKO compared to DKO ESCs, a weak GFP-positive population persisted in the TKO (Figure 2A, B)." This description appears inconsistent with both Figures 2A/2B and with Figure 4F, which shows very weak MERVL-GFP signals in TKO cells. Can the authors clarify this discrepancy? Additionally, what is the potential mechanism by which the TKO appears to show an increased 2CLC-like population compared to the DKO?
7. Fig. 6B, Dux expression is very low in ESCs (considering the fact that it is only expressed in 2CLC, which is a tiny fraction of total population), it is puzzling why Dux KO has so many DEGs.
8. Fig. 6D, a Venn diagram would be much clearer to show that Pramel7 OE can rescue Dux KO downregulated genes.

Referee #2:

The manuscript by Yoshioka and colleagues explores the role of Ctbp1/2 in 2CLCs. They previously showed that CtBP1/2 are essential for the transition from formative to naïve pluripotency in mESCs. In this study, the authors show that disruption of Ctbp1/2 activates two distinct sets of genes in mESCs: the first set is transiently upregulated in a DUX-dependent manner during minor ZGA, and the second contains maternally expressed genes and the upregulated genes in a DUX-independent manner during major ZGA.

Major comments:
1. It is stated in Abstract that DUX is dispensable for mouse embryos, suggesting that DUX-independent molecular pathways are not recapitulated in 2CLCs. However, the authors do not convincingly address this. They do not show results of how different (or similar) DUX-dependent and -independent pathways operate in 2C embryos and 2CLCs. The authors shall consider performing experiments with 2C embryos (early embryos) and addressing the questions of how CtBP1/2 and Pramel7 work in early embryos and then comparing the pathways between 2C embryos and 2CLCs.
2. It is not clear how CtBP1/2 function in DUX-dependent and -independent pathways. A number of 2CLC repressors have been identified. Without molecular mechanisms of how CtBP1/2 work, they should be considered to be but another 2CLC repressor.
3. Similarly, it is not clear how Pramel7 works in the pathways. Pramel7 is depicted to be involved in UHRF1 degradation in the model shown in Figure 7. However, no result to support this is present in the manuscript. In addition, this model does not explain why Dux KO embryos develop normally at all.
Other comments:
1. Figure 1D: Ctbp1/2 DKO ESCs exhibited an over 50 fold increase (7.4%) in GFP-positive cells. Why has the number of GFP-positive cells reached a plateau at 7.4%? Should the prolonged culture increase the number of the positive cells?
2. Figure 1E: Pluripotency-associated factors such as OCT4 were markedly reduced. However, a large number of cells still express OCT4. What might cause the heterogeneity in gene expression in the CtBP1/2 DKO culture?
3. Figure 2A B: While the strong GFP-positive population was markedly reduced in TKO compared to DKO ESCs, a weak GFP-positive population persisted in the TKO. How do the authors define "strong" and "weak"? Do a weak GFP-positive population really 'persist' in the TKO? Alternatively, do they increase in the TKO?
4. Figure 2C: Why does the expression level of Pramel7 increase in the TKO much more than in the DKO? Is there any Pramel7 repressor, in addition to CtBP1/2?
5. Figure 3A and Figure 4A: The authors should conduct ChIP-qPCR/CUT&RUN to demonstrate that Pramel7 is a direct target of CtBP1/2 (that is, CtBP1/2 directly bind to the Pramel7 locus).
6. Figure 5E F: PRAMEL7 indirectly promotes the expression of minor ZGA genes through Dux activation. However, the number of MERVL-EGFP positive cells is small, much smaller than those observed in the DKO shown in Figure 1. Thus, it is difficult to explain the increase of MERVL-EGFP positive cells in CtBP1/2 DKO solely by the activity of Pramel7. In addition, Figure 5B shows that Pramel7 is activated in L2C and later stages (4C to ICM) of embryos. However, Dux is activated in E2C in embryos. This indicates that what observed in mESCs (Pramel7 -> Dux -> minor ZGA genes) should not occur in embryos. This also raises the question of why Dux is not activated during L2C to ICM where Pramel7 is activated.
7. The preparation of the manuscript is sloppy. A number of typographical errors. In addition, Church et al 2009 (line 400, page 23) is missing in References. Many other citations are not correctly cited in References in a way they should.

Referee #3:

The manuscript by Kazuma Yoshioka et al. investigated the roles of CtBP1/2 in mESC and proposed a dual regulatory framework that induces a totipotent-like state. Through transcriptome analysis, the authors observed minor ZGA gene activation and an increased population of 2CLC in Ctbp1/2 double knockout mESC. By comparing Ctbp1/2 DKO and Ctbp1/2/Dux TKO mESC lines, they concluded that CtBP1/2 repress 2CLC state through both Dux-dependent and -independent ways. Furthermore, they identified Pramel7 as a key mediator linking Ctbp1/2 loss to downstream transcriptome activation. Overall, the authors reveal the involvement of CtBP1/2 and Pramel7 in totipotency and ZGA regulation. However, the current manuscript does not provide sufficient evidence to clarify the relationship between CtBP1/2, Dux, and Pramel7.

Major:

1. The characterization and analysis of Mervl-EGFP positive cells in Ctbp1/2 DKO mESC is not comprehensive enough. For example, how does the transcriptome of these cells change? It is important to assess their similarity to previously reported 2CLCs (PCA analysis with published 2CLC datasets), as the totipotency state of these cells is the basis of the entire study. This also applies to Mervl-EGFP positive cells in Ctbp1/2/Dux TKO line.

2. In Figure 2A, the ratio of Mervl-EGFP positive cells appears higher in Ctbp1/2/Dux TKO cells then in Ctbp1/2 DKO cells. Given that Dux is a known master activator for 2CLC state, why does its KO in Ctbp1/2 DKO background further expand the 2CLC population? In line 205-208, the authors refer to "strong" and "weak" GFP-positive populations, it would be clearer to define these subpopulations and show their exact proportions, helping readers judge whether the described changes are indeed "markedly reduced" (Line 206) or "persisted" (Line 207).

3. In Figure 4B, please also label the peak corresponding to Gata1-DE. Given the numerous strong Dux peaks near Pramel7 and Gata1 loci, how can the authors exclude a direct role of Dux? In Figure 4D and 4E, could be the reporter activated by Dux? Alternatively, might Dux activate Pramel7 and Gata1 by distal regulatory regions marked by Dux peaks shown in 4B?

4. The rationale for selecting Pramel7 as the downstream effector is not clearly explained in the text. Why not consider Pramel6, which shows similar expression pattern to Pramel7 (Figure 5B)? The manuscript states that expression levels of Pramel7 in Dux KO cells was "unaffected" (Line 411), yet Figure 5K shows a dramatic decrease, please clarity this discrepancy.

5. In Line 484-485, the authors state that "downregulation of Pramel7 expression is major cause of the transcriptional abnormalities in Dux KO ESCs". Then, why does Pramel7 overexpression fail to increase the proportion of Mervl-EGFP positive cells in Dux KO cells in Figure 5F?

6. The authors conclude that Pramel7 activation by the loss of Ctbp1/2 "regulates maternally, major ZGA and post-ZGA genes through modulation of DNA methylation mediated by UHRF1 degradation in 2CLCs" (Line 502-503). First, does Pramel7 KO in Ctbp1/2 DKO mESC reduce the proportion of Mervl-EGFP positive cells? What about in Ctbp1/2/Dux TKO line? Second, the DNA demethylation in 2CLCs induced by Ctbp1/2 DKO should be examined, at least at several key loci, such as Dux locus.

7. Based on presented data, the relationship between CtBP1/2, Dux, and Pramel7 remains unclear. RNA-seq overlap analysis provides correlation but not causation. Performing Dux ChIP-seq in Ctbp1/2 DKO, Pramel7 KO, and Ctbp1/2/Pramel7 TKO cells would provide necessary information to elucidate the regulatory interactions among CtBP1/2, Dux, and Pramel7 in mESC.

Minor:

1. Figure 1, a heatmap showing the expression of minor ZGA genes in WT and DKO cell lines would improve clarity.

2. Ensure consistency in terminology, use either "RT-qPCR" or "qRT-PCR".

3. Please add scale bars to the images in Figure 4F and 5E.

4. In line 209, the authors cite Guo et al., 2019, but that study did not report the expression levels of Pramel7. If the authors used their data, please include a panel showing Pramel7 expression levels in the present study.

Referee #1:

2C-like cells (2CLCs) emerged from mESCs partially resemble the transcription program of mouse 2C embryos. These cells provide a valuable model for understanding the 2C embryos and totipotency program in vitro. Yoshioka and colleagues sought to understand how the transcription program is regulated in 2CLCs. Through analyzing their previous Ctbp1/2 DKO mESCs, they found Ctbp1/2 may work as repressors of the 2C program in mESCs. The authors found a DUX independent pathway was activated in Ctbp1/2 DKO which the authors propose could be partially attributed to Pramel7.

Overall, this article presented valuable insights into the in vitro modeling of the 2C program of mouse early embryos. The Ctbp1/2 DKO mESCs provided a rich resource to study the nature of 2CLC and its transcription program. The most interesting result in the paper is the persistence of MERVL-reporter positive cells in the absence of Dux. However, its nature remains elusive. The current model proposed by the authors is interesting but alternative scenarios still exist, which would undermine the major message of the paper. This needs to be clarified before the paper is considered for publication. Please see below for my detailed comments.

Comment 1.
The expansion of 2CLC population in Ctbp1/2 DKO mESCs, which is independent of Dux (Dux KO even increased the 2CLC population), is one of the most surprising results in this article. While 2C genes may include a large number of genes, those that are specifically expressed during minor ZGA (MERVL, Zscan4, Usp17l, Tdpoz4) are most characteristic ones. Given most typical 2C-marker genes (Zscan4, Usp17l, Tdpoz4) were still fully dependent on DUX, this persisting cell population may mainly represent the high activity of MERVL-reporter. Therefore, one alternative possibility is that Ctbp1/2 DKO upregulates an activator of MERVL, leading to a persistent 2C population even in the absence of DUX.

Response 1.
We thank the reviewer for this important comment. We agree that the persistent MERVL-EGFP-positive population observed in *Ctbp1/2/Dux* TKO ESCs should not be interpreted as canonical 2CLCs. In the revised manuscript, we therefore separated the reporter-positive population into GFP+ and GFP+++ fractions using fixed fluorescence gates (Fig. 2A, B) and now describe the TKO population as a DUX-independent

MERVL-EGFP-weak-positive population, rather than as bona fide MERVL-high 2CLCs.

Consistent with the reviewer's concern, characteristic minor ZGA genes, including *Zscan4*, *Usp17l* family genes and *Tdpoz* genes, remained largely DUX-dependent. However, gene signature analysis showed that *Ctbp1/2/Dux* TKO ESCs retained signatures associated with the early/intermediate phase of 2CLC transition, whereas the canonical MERVL-high/minor-ZGA signature was impaired (Fig. 3D, E). In addition, GATA1, a CtBP2-associated major ZGA-related factor, was detected in the MERVL-EGFP-positive population (Fig. 5H).

We also examined *Obox* family genes as candidate DUX-independent activators and found that *Obox4* was induced by *Ctbp1/2* loss, but this induction was attenuated by *Dux* deletion (Appendix Fig. S2I). These results suggest that the residual GFP+ population is molecularly distinct from the canonical DUX-dependent MERVL-high/minor-ZGA state, although the precise factor responsible for DUX-independent weak MERVL-EGFP activation remains to be identified.

Comment 2.

For the cluster 2-4 genes that are activated in CtBP1/2 ("Dux-independent 2C genes"), the fact that some are expressed in oocytes while others are expressed in embryos may also raise a possibility that they do not have specificity for embryo expression, but are general targets (direct or indirect) of CtBP1/2 that happens to overlap with genes expressed in oocytes and embryos. It is probably not surprising that a general list of genes also include those that are expressed in oocytes or early embryos. As a comparison, cluster 1 is very specific for early 2C and late 2C embryos (Fig. 2H). This is especially important given Ctbp1/2 are general repressors involved in multiple cellular processes. A deep analysis should be performed to critically distinguish whether the up-regulation of Dux-independent 2C genes in Ctbp1/2 DKO cells is specific.

Response 2.

We thank the reviewer for this important point. Because CtBP1/2 are broad transcriptional corepressors, we agree that it is important to distinguish selective developmental gene activation from general derepression. To address this, we performed K-means clustering of the 2,000 most variable genes across WT, Dux KO, *Ctbp1/2* DKO and *Ctbp1/2/Dux* TKO ESCs (Fig. 3F; Appendix Fig. S2J).

Gene set enrichment analysis across individual clusters showed that the induced genes

were not uniformly derepressed, but instead segregated into developmentally biased modules (Fig. 3G; Appendix Fig. S2K, L). Cluster 1 was enriched for minor ZGA genes, Cluster 3 for major ZGA genes and trophectoderm-associated genes, Cluster 4 for primitive endoderm-associated genes, and Cluster 5 for formative/committed pluripotency-associated genes. By contrast, p53-associated genes and housekeeping genes were not preferentially enriched (Fig. 3G; Appendix Fig. S2L).
These results do not exclude the possibility that some genes are general CtBP1/2 targets, but they argue against indiscriminate derepression and support the idea that loss of *Ctbp1/2* preferentially affects several developmentally biased gene modules.

Comment 3.
As an important test of these cells as "2C-like" cells, would Ctbp1/2/Dux TKO MERVL positive mESCs contribute to extraembryonic tissue? A minor question is do these cells also exhibit the "cycling-in-and-out" feature of 2CLCs? If the authors sort the MERVL positive cells and culture them, would they keep MERVL expression?

Response 3.
We thank the reviewer for this important comment. We agree that contribution to extraembryonic tissues would be a stringent functional test of bona fide totipotent-like potential. We did not perform blastocyst injection assays in the present revision, and therefore we do not claim that the weak MERVL-EGFP-positive population in *Ctbp1/2/Dux* TKO ESCs has bona fide extraembryonic contribution potential. We attempted to derive trophoblast stem cell-like lines from WT and *Ctbp1/2* DKO ESCs under TS culture conditions. Although cells could be maintained under TS culture conditions, robust induction of extraembryonic lineage markers was not observed. However, K-means clustering and gene set enrichment analysis revealed that one of the gene clusters upregulated in *Ctbp1/2 DKO* and *Ctbp1/2/Dux* TKO ESCs was enriched for TE-associated genes (Fig. 3F,G). These results suggest that *Ctbp1/2*-deficient ESCs acquire partial extraembryonic lineage-associated molecular features**,** but do not demonstrate bona fide trophoblast potential or functional contribution to extraembryonic tissues**.**

Regarding the cycling-in-and-out behavior, we sorted MERVL-EGFP-positive cells from *Ctbp1/2* DKO and *Ctbp1/2/Dux* TKO ESCs and re-cultured them. In both genotypes, GFP-negative cells reappeared after re-culture, indicating that the MERVL-EGFP-positive state is reversible rather than fixed (Fig. 2F). Time-lapse imaging further

supported reversible transitions and revealed distinct GFP dynamics among WT, *Ctbp1/2* DKO and *Ctbp1/2/Dux* TKO ESCs (Fig. 2G–K). We have revised the manuscript to clarify that *Ctbp1/2/Dux* TKO ESCs exhibit a reversible weak MERVL-EGFP-positive state, but that this state should not be equated with functional totipotency.

Comment 4.

Given there are more than 2500 differential expressed genes in CtBP1/2 KO mESC, what's the phenotype of the KO cells? Do CtBP1/2-deficient mESCs proliferate normally? If the cells are stressed or close to losing pluripotency/viability, this may also confound the interpretation of the observed 2CLC increase, given p53 is known to activate Dux.

Response 4.

We thank the reviewer for this important comment. In response to the reviewer's concern, we examined the growth rate and AP-positive colony-forming activity of WT, *Dux* KO, *Ctbp1/2* DKO and *Ctbp1/2/Dux* TKO ESCs. *Ctbp1/2* DKO ESCs showed slower growth than WT and Dux KO ESCs, and this growth defect was largely rescued by additional Dux deletion (Fig. 2C). This result suggests that activation of the DUX-dependent minor ZGA program contributes to the reduced proliferation of *Ctbp1/2*-deficient ESCs.

We also examined AP-positive colony formation as an indicator of ESC self-renewal. *Ctbp1/2* DKO ESCs formed fewer AP-positive colonies than WT ESCs, and this reduction was not restored by additional *Dux* deletion (Fig. 2D,E). Thus, although the growth defect of *Ctbp1/2* DKO ESCs was largely rescued by *Dux* deletion, the reduced AP-positive colony-forming activity was not fully attributable to DUX-dependent 2CLC activation.

Importantly, *Ctbp1/2* DKO and *Ctbp1/2/Dux* TKO ESCs remained viable and could be continuously maintained in culture, arguing against a severe loss of viability. We further examined p53-associated gene expression and found no significant activation of a curated p53 target gene set or canonical p53 target genes, including *Cdkn1a* and *Mdm2* (Appendix Fig. S2G,H). These results support the interpretation that MERVL-EGFP activation in *Ctbp1/2*-deficient ESCs is not simply a consequence of a p53-mediated stress response.

Comment 5.

PRAMEL7 OE can activate MERVL-reporter but in Dux-dependent manner, which indicates that its function on classic 2C genes (minor ZGA genes) is still Dux-dependent. Given it is not the factor that can activate MERVL without DUX, the real key factor that enable this surprising result is still unknown.

Response 5.

We thank the reviewer for this critical comment. We agree that PRAMEL7 is not sufficient to activate the canonical MERVL reporter program in the absence of DUX. In the revised manuscript, we therefore distinguish between two PRAMEL7-associated outputs: a DUX-dependent effect on the canonical MERVL-high/minor-ZGA program and a DUX-independent effect on a subset of major ZGA- and post-ZGA-associated genes. PRAMEL7 overexpression modestly increased the MERVL-EGFP-positive population in WT ESCs, but this effect was abolished in the *Dux* KO background (Fig. 6E,F). Thus, PRAMEL7 does not explain the residual weak MERVL-EGFP-positive population observed in *Ctbp1/2/Dux* TKO ESCs.

To address potential DUX-independent activators of this weak MERVL-EGFP-positive population, we examined the expression of *Obox* family genes in WT, Dux KO, *Ctbp1/2* DKO and *Ctbp1/2/Dux* TKO ESCs (Appendix Fig. S2I). Among them, *Obox4* was upregulated upon loss of *Ctbp1/2*, but this upregulation was attenuated, although not completely abolished, by *Dux* deletion. These results suggest that OBOX4 may contribute to this phenotype, but its induction alone is unlikely to fully explain the weak MERVL-EGFP-positive population observed in *Ctbp1/2/Dux* TKO ESCs. We have revised the manuscript to explicitly state that the factor responsible for DUX-independent weak MERVL-EGFP activation remains to be identified.

Comment 6.

Figures 2A and 2B show that 24.95% of CtBP1/2/Dux TKO cells are MERVL-GFP-positive, which is even higher than in CtBP1/2 DKO cells (14.09%). However, the manuscript states: "While the strong GFP-positive population was markedly reduced in TKO compared to DKO ESCs, a weak GFP-positive population persisted in the TKO (Figure 2A, B)." This description appears inconsistent with both Figures 2A/2B and with Figure 4F, which shows very weak MERVL-GFP signals in TKO cells. Can the authors clarify this discrepancy? Additionally, what is the potential mechanism by

which the TKO appears to show an increased 2CLC-like population compared to the DKO?

Response 6.

We thank the reviewer for pointing out this important issue. We agree that our original wording was not sufficiently clear because it did not explicitly distinguish between total MERVL-EGFP-positive frequency and reporter intensity. To clarify this point, we separated the MERVL-EGFP-positive population into GFP+ and GFP+++ fractions using fixed gates in the revised figure (Fig. 2A,B). This analysis showed that *Ctbp1/2* DKO ESCs contained both GFP+ and GFP+++ populations, whereas the GFP+++ population was eliminated in *Ctbp1/2/Dux* TKO ESCs (Fig. 2A,B). In contrast, the lower-intensity GFP+ fraction remained detectable and was expanded in TKO ESCs (Fig. 2A,B). Thus, the apparent discrepancy arises from the difference between total GFP-positive frequency and GFP intensity.

Our interpretation is that DUX is required for progression from the weak GFP+ state to the robust GFP+++ state. In *Ctbp1/2* DKO ESCs, a subset of GFP+ cells progresses to the DUX-dependent GFP+++ state, which is associated with reduced proliferative capacity and frequent cellular collapse. In contrast, in *Ctbp1/2/Dux* TKO ESCs, cells fail to progress to the GFP+++ state and instead remain in the weak GFP+ state. Because these GFP+ cells retain a greater proliferative capacity than GFP+++ cells, they can accumulate over time, resulting in a higher total percentage of GFP-positive cells despite lower GFP intensity.

This interpretation is supported by our time-lapse analysis, which showed that WT cells frequently reached high GFP intensity and often failed to continue dividing, whereas DKO and TKO cells more often remained at lower GFP intensity and continued to divide (Fig. 2G–K). Consistently, gene signature analysis showed that *Ctbp1/2/Dux* TKO ESCs retained early/intermediate 2CLC-transition signatures, whereas the canonical minor ZGA/MERVL-high program was impaired (Fig. 3D,E). We have revised the text throughout the manuscript to distinguish GFP+ from GFP+++ cells and to avoid conflating weak MERVL-EGFP positivity with the canonical MERVL-high 2CLC state.

Comment 7.

Fig. 6B, Dux expression is very low in ESCs (considering the fact that it is only expressed in 2CLC, which is a tiny fraction of total population), it is puzzling why Dux KO has so many DEGs.

Response 7.

We thank the reviewer for this important point. We agree that the low bulk expression level of *Dux* in ESC cultures makes the large number of DEGs in *Dux* KO ESCs appear puzzling if all DEGs are assumed to be direct DUX targets. However, this is not our interpretation. Because DUX is expressed transiently in a rare subpopulation of ESCs, *Dux* deletion is expected to eliminate the canonical MERVL-high 2CLC state and thereby affect not only direct DUX target genes but also downstream transcriptional and epigenetic states associated with the 2CLC program.

To address this issue, we compared genes downregulated in *Dux* KO ESCs with those downregulated in *Pramel7* KO ESCs and found a partial overlap (Fig. 8A). We further examined whether these genes were related to DNA methylation-dependent repression by comparing them with genes upregulated in *Dnmt1* KO ESCs (Fig. 8B,C). This analysis showed that a subset of genes downregulated in *Dux* KO ESCs was restored by PRAMEL7 overexpression in the Dux KO background and overlapped with genes upregulated in *Dnmt1* KO ESCs (Fig. 8C). In addition, UHRF1 downregulation in ZSCAN4-positive cells was impaired by *Pramel7* KO (Fig. 8E,F).

These results suggest that the large number of DEGs in Dux KO ESCs reflects a combination of direct DUX-dependent transcriptional effects and indirect consequences of impaired downstream PRAMEL7/UHRF1-associated epigenetic regulation. We have revised the manuscript to clarify that we do not interpret all *Dux* KO-associated DEGs as direct DUX target genes.

Comment 8.

Fig. 6D, a Venn diagram would be much clearer to show that Pramel7 OE can rescue Dux KO downregulated genes.

Response 8.

We thank the reviewer for this helpful suggestion. In response, we added a Venn diagram comparing three gene sets: genes downregulated in *Dux* KO ESCs, genes

upregulated by PRAMEL7 overexpression in the *Dux* KO background, and genes upregulated in *Dnmt1* KO ESCs (Fig. 8C). This analysis showed that 663 of the 1,135 genes downregulated in *Dux* KO ESCs were upregulated by PRAMEL7 overexpression in the *Dux* KO background (Fig. 8C), indicating that PRAMEL7 overexpression partially restores the transcriptional defects caused by *Dux* deletion. Among these rescued genes, 345 were also upregulated in *Dnmt1* KO ESCs (Fig. 8C), suggesting that a substantial fraction of PRAMEL7-mediated rescue is associated with DNA methylation-dependent repression.

Referee #2:

The manuscript by Yoshioka and colleagues explores the role of Ctbp1/2 in 2CLCs. They previously showed that CtBP1/2 are essential for the transition from formative to naïve pluripotency in mESCs. In this study, the authors show that disruption of Ctbp1/2 activates two distinct sets of genes in mESCs: the first set is transiently upregulated in a DUX-dependent manner during minor ZGA, and the second contains maternally expressed genes and the upregulated genes in a DUX-independent manner during major ZGA.

Comment 1.

It is stated in Abstract that DUX is dispensable for mouse embryos, suggesting that DUX-independent molecular pathways are not recapitulated in 2CLCs. However, the authors do not convincingly address this. They do not show results of how different (or similar) DUX-dependent and -independent pathways operate in 2C embryos and 2CLCs. The authors shall consider performing experiments with 2C embryos (early embryos) and addressing the questions of how CtBP1/2 and Pramel7 work in early embryos and then comparing the pathways between 2C embryos and 2CLCs.

Response 1.

We thank the reviewer for this important comment. We agree that direct functional and transcriptomic analyses in early embryos would strengthen the study and help determine whether the CtBP1/2–PRAMEL7 axis identified here also operates *in vivo*. However, the main scope of the present study is to define DUX-dependent and DUX-independent transcriptional programs in the mESC/2CLC system. We therefore do not claim that the pathway identified here fully explains ZGA regulation in embryos.

To address the reviewer's concern, we revised the Abstract, Introduction, and Discussion to clarify the scope of our conclusions. In addition, we re-analyzed published embryo RNA-seq data and showed that *Pramel7* is upregulated at the 2-cell stage in both WT and *Dux* KO embryos (Appendix Fig. S2A). This result supports the idea that at least some major ZGA-associated genes can be activated in embryos independently of DUX. However, because we did not perform direct functional perturbation of CtBP1/2 or PRAMEL7 in embryos, we now explicitly state that direct embryo analyses will be required to determine whether the CtBP1/2–PRAMEL7 axis also functions *in vivo*.

Comment 2.

It is not clear how CtBP1/2 function in DUX-dependent and -independent pathways. A number of 2CLC repressors have been identified. Without molecular mechanisms of how CtBP1/2 work, they should be considered to be but another 2CLC repressor.

Response 2.

We thank the reviewer for this important comment. We agree that a number of 2CLC repressors have been identified and that the full CtBP1/2-centered regulatory network remains to be completely resolved. However, our revised data provide mechanistic evidence that CtBP1/2 are not simply another upstream repressor of *Dux*. Rather, CtBP1/2 also directly constrain a DUX-independent regulatory arm involving major ZGA-associated genes and post-ZGA-associated genes.

A key point of our study is that CtBP1/2 do not appear to function primarily as direct repressors of *Dux*, unlike many previously characterized 2CLC repressors. Instead, CtBP2 was preferentially associated with rapid-response genes rather than delayed-response genes (Fig. 5A). Genome browser analysis showed CtBP2 occupancy at regulatory regions of major ZGA-associated genes such as *Pramel7* and *Gata1*, whereas DUX was preferentially associated with delayed-response loci such as *Duxf3* and *Zscan4c* (Fig. 5B,C). Furthermore, enhancer reporter assays showed that the CtBP2-associated distal regulatory regions of *Pramel7* and *Gata1* were activated in *Ctbp1/2* DKO ESCs and repressed again by CtBP2 re-expression (Fig. 5D,E). We also confirmed CtBP2 binding at these regulatory regions by ChIP-qPCR (Fig. 5G). Thus, for these loci, the revised manuscript provides direct molecular evidence that CtBP2 represses DUX-independent major ZGA-associated genes.

In addition, our transcriptome analysis showed that many PRAME family genes were upregulated upon *Ctbp1/2* loss (Fig. 6A). Importantly, this group included both DUX-dependent and DUX-independent PRAME family members, suggesting that CtBP1/2 broadly restrict PRAME-associated transcriptional programs rather than acting only through a single downstream effector. However, we have revised the text to avoid implying that all PRAME family genes are direct CtBP2 targets.

Therefore, the main mechanistic advance of this study is not simply that CtBP1/2 repress 2CLC emergence, but that they constrain both the canonical DUX-dependent minor ZGA program and a DUX-independent major ZGA/PRAME-associated regulatory arm. We have revised the Discussion to make this point clearer as follows:

"Unlike many previously characterized 2CLC repressors, which primarily act through the repression of *Dux* or its upstream regulators, CtBP1/2 directly repress major ZGA-associated genes, including *Pramel7* and *Gata1*, thereby constraining a DUX-independent regulatory arm in addition to the canonical DUX-dependent program."

Comment 3.

Similarly, it is not clear how Pramel7 works in the pathways. Pramel7 is depicted to be involved in UHRF1 degradation in the model shown in Figure 7. However, no result to support this is present in the manuscript. In addition, this model does not explain why Dux KO embryos develop normally at all.

Response 3.
We thank the reviewer for this critical comment. To clarify how PRAMEL7 functions in this pathway, we added new data examining UHRF1 protein levels and DNA methylation status. Western blot analysis showed that PRAMEL7 overexpression reduced UHRF1 protein levels in ESCs (Fig. 7A), consistent with the previously reported role of PRAMEL7 in promoting UHRF1 degradation. Reduced representation bisulfite sequencing further showed reduced DNA methylation at the *Duxf3* locus in PRAMEL7-overexpressing ESCs (Fig. 7B). In addition, UHRF1 protein levels were reduced in *Ctbp1/2* DKO ESCs and more prominently in *Ctbp1/2/Dux* TKO ESCs, in which *Pramel7* expression remained elevated (Fig. 7F; Fig. 3A). Bisulfite sequencing also showed reduced DNA methylation at the *Duxf3* locus in Ctbp1/2 DKO ESCs compared with WT ESCs (Fig. 7G).

To examine whether PRAMEL7-induced genes are associated with DNA methylation-dependent regulation, we compared genes upregulated by PRAMEL7 overexpression with genes upregulated in *Dnmt1* KO ESCs (Fig. 7C). A substantial subset of PRAMEL7-induced genes overlapped with *Dnmt1* KO-upregulated genes, and these overlapping genes showed promoter-proximal DNA hypomethylation (Fig. 7D,E). Furthermore, genes commonly upregulated in *Ctbp1/2/Dux* TKO and *Dnmt1* KO ESCs were enriched for developmental gene sets associated with major ZGA and post-ZGA programs (Fig. 7H,I). Together, these results support the idea that PRAMEL7 contributes to the activation of a subset of DUX-independent genes through UHRF1 downregulation and DNA demethylation-linked regulation.

We also revised the Discussion to clarify why *Dux* KO embryos can still develop. Re-analysis of published embryo RNA-seq data showed that *Pramel7* is upregulated at the 2-cell stage in both WT and Dux KO embryos (Appendix Fig. S2A). Thus, although *Pramel7* expression is reduced in *Dux* KO ESCs, *Pramel7* can still be activated in *Dux* KO embryos. We propose that this difference may reflect embryo-specific regulatory inputs, including maternally deposited activators such as OBOX family members, together with developmental clearance of maternal CtBP1/2 activity after fertilization. We have revised the Discussion as follows:

"In embryos, published RNA-seq data suggest that minor ZGA genes, including direct DUX targets, are activated with delayed kinetics in *Dux* KO embryos, whereas major ZGA genes such as *Pramel7* are still upregulated at the middle 2-cell stage (Guo *et al*, 2019). This difference may reflect the presence of maternally deposited activators, including OBOX family members (Ji *et al*, 2023), together with the progressive decline of maternal *Ctbp1/2* transcripts after fertilization (Zhang *et al*, 2016), which may help support DUX-independent activation of major ZGA genes *in vivo*."

Comment 4.

Figure 1D: Ctbp1/2 DKO ESCs exhibited an over 50 fold increase (7.4%) in GFP-positive cells. Why has the number of GFP-positive cells reached a plateau at 7.4%? Should the prolonged culture increase the number of the positive cells?

Response 4.

We thank the reviewer for this important point. We agree that prolonged culture would be expected to increase the MERVL-EGFP-positive fraction if GFP-positive cells were irreversibly generated and continuously accumulated. To test whether the MERVL-EGFP-positive fraction is instead maintained by dynamic transitions between GFP-positive and GFP-negative states, we sorted MERVL-EGFP-positive cells from *Ctbp1/2* DKO and *Ctbp1/2/Dux* TKO ESCs and re-cultured them.

After 8 days of re-culture, GFP-negative cells reappeared in both genotypes (Fig. 2F), indicating that the MERVL-EGFP-positive state is reversible rather than fixed. These results suggest that the percentage of MERVL-EGFP-positive cells observed in bulk culture (Fig. 1D and Fig. 2A,B) reflects a dynamic equilibrium between entry into and exit from the GFP-positive state, rather than continuous accumulation of GFP-positive cells during prolonged culture.

Comment 5.

Figure 1E: Pluripotency-associated factors such as OCT4 were markedly reduced. However, a large number of cells still express OCT4. What might cause the heterogeneity in gene expression in the CtBP1/2 DKO culture?

Response 5.

We thank the reviewer for this important comment regarding heterogeneous OCT4 expression in *Ctbp1/2* DKO ESCs. In Fig. 1E, OCT4 expression was reduced in MERVL-EGFP-positive cells, but many cells in the culture still retained OCT4 expression. We interpret this heterogeneity as reflecting the coexistence of MERVL-EGFP-positive cells, MERVL-EGFP-negative cells, and transitional cells within the *Ctbp1/2* DKO culture.

Consistent with this interpretation, when MERVL-EGFP-positive cells were sorted and re-cultured, GFP-negative cells reappeared after 8 days (Fig. 2F), indicating that the MERVL-EGFP-positive state is reversible rather than fixed. Thus, OCT4 repression likely occurs transiently during entry into the MERVL-EGFP-positive state, and heterogeneous OCT4 expression reflects dynamic transitions between pluripotent-like and MERVL-EGFP-positive states.

Comment 6.

Figure 2A B: While the strong GFP-positive population was markedly reduced in TKO compared to DKO ESCs, a weak GFP-positive population persisted in the TKO. How do the authors define "strong" and "weak"? Do a weak GFP-positive population really 'persist' in the TKO? Alternatively, do they increase in the TKO?

Response 6.

We thank the reviewer for this important comment. We agree that the terms “strong” and “weak” were not sufficiently defined in the original manuscript. In the revised manuscript, we now define these populations using fixed MERVL-EGFP fluorescence intensity gates and refer to them as GFP+ and GFP+++ fractions (Fig. 2A,B).

Based on this revised gating strategy, *Ctbp1/2* DKO ESCs contained both GFP+ and GFP+++ populations, whereas the GFP+++ fraction was largely lost in *Ctbp1/2/Dux* TKO ESCs (Fig. 2A,B). In contrast, the lower-intensity GFP+ fraction not only persisted but was expanded in TKO ESCs relative to DKO ESCs (Fig. 2A,B).

These results suggest that Dux deletion impairs progression from the weak GFP+ state to the robust GFP+++ state, leading to accumulation of cells in the GFP+ fraction. We have revised the Results section to describe this distinction explicitly.

Comment 7.

Figure 2C: Why does the expression level of *Pramel7* increase in the TKO much more than in the DKO? Is there any Pramel7 repressor, in addition to CtBP1/2?

Response 7.

We thank the reviewer for this important comment. We agree that *Pramel7* expression is higher in *Ctbp1/2/Dux* TKO ESCs than in *Ctbp1/2* DKO ESCs.

One possible explanation is that DUX-dependent and DUX-independent transcriptional trajectories represent distinct or competing cell-state transitions. In *Ctbp1/2* DKO ESCs, a subset of GFP+ cells progresses to the DUX-dependent GFP+++ state, which is associated with reduced proliferative capacity and frequent cellular collapse. In contrast, in *Ctbp1/2/Dux* TKO ESCs, cells fail to progress to the GFP+++ state and instead accumulate in the lower-intensity GFP+ state (Fig. 2A,B). Because *Pramel7* remains highly expressed in this DUX-independent GFP+ state, the increased proportion of these cells may contribute to the higher bulk *Pramel7* expression observed in TKO ESCs (Fig. 3A).

Consistent with this interpretation, gene signature analysis showed that *Ctbp1/2/Dux* TKO ESCs retained early/intermediate 2CLC-transition signatures, whereas the canonical minor ZGA/MERVL-high program was impaired (Fig. 3D,E). We also examined *Obox* family genes as potential upstream regulators, but *Obox* expression did not fully explain the higher *Pramel7* expression in the TKO background (Appendix Fig. S2I). We have revised the manuscript to clarify that *Pramel7* is further increased in *Ctbp1/2/Dux* TKO ESCs, while the precise mechanism underlying this increase remains unresolved.

Comment 8.

Figure 3A and Figure 4A: The authors should conduct ChIP-qPCR/CUT&RUN to demonstrate that Pramel7 is a direct target of CtBP1/2 (that is, CtBP1/2 directly bind to the Pramel7 locus).

Response 8.

We thank the reviewer for this important suggestion. To directly test whether CtBP2 binds to the Pramel7 locus, we performed ChIP-qPCR using doxycycline-inducible FLAG-CtBP2 ESCs. This analysis confirmed CtBP2 enrichment at the *Pramel7* distal enhancer region, as well as at the *Gata1* regulatory region (Fig. 5G).

This result is consistent with public CtBP2 ChIP-seq data showing CtBP2 occupancy near the *Pramel7* and *Gata1* loci (Fig. 5B). In addition, luciferase reporter assays showed that the CtBP2-associated distal regulatory regions of *Pramel7* and *Gata1* were activated in Ctbp1/2 DKO ESCs and repressed again by CtBP2 re-expression (Fig. 5D,E).

Together, these results support the conclusion that *Pramel7* and *Gata1* are direct CtBP2-repressed targets in ESCs. We have added the ChIP-qPCR data to Fig. 5G and revised the Results section accordingly.

Referee #3:

The manuscript by Kazuma Yoshioka et al. investigated the roles of CtBP1/2 in mESC and proposed a dual regulatory framework that induces a totipotent-like state. Through transcriptome analysis, the authors observed minor ZGA gene activation and an increased population of 2CLC in Ctbp1/2 double knockout mESC. By comparing Ctbp1/2 DKO and Ctbp1/2/Dux TKO mESC lines, they concluded that CtBP1/2 repress 2CLC state through both Dux-dependent and -independent ways. Furthermore, they identified Pramel7 as a key mediator linking Ctbp1/2 loss to downstream transcriptome activation. Overall, the authors reveal the involvement of CtBP1/2 and Pramel7 in totipotency and ZGA regulation. However, the current manuscript does not provide sufficient evidence to clarify the relationship between CtBP1/2, Dux, and Pramel7.

Comment 1.

The characterization and analysis of Mervl-EGFP positive cells in Ctbp1/2 DKO mESC is not comprehensive enough. For example, how does the transcriptome of these cells change? It is important to assess their similarity to previously reported 2CLCs (PCA analysis with published 2CLC datasets), as the totipotency state of these cells is the basis of the entire study. This also applies to Mervl-EGFP positive cells in Ctbp1/2/Dux TKO line.

Response 1.

We thank the reviewer for this important comment. We agree that the characterization of MERVL-EGFP-positive cells in *Ctbp1/2* DKO and *Ctbp1/2/Dux* TKO ESCs needed to be clarified. In the revised manuscript, we separated the reporter-positive population into GFP+ and GFP+++ fractions using fixed fluorescence gates and now show their proportions separately (Fig. 2A,B).

To further characterize the identity of the residual GFP-positive population in *Ctbp1/2/Dux* TKO ESCs, we performed gene signature analysis using gene sets associated with sequential stages of 2CLC transition, based on the trajectory reported by Rodriguez-Terrones et al. (2018). Specifically, we examined signatures corresponding to the transitions from ESCs to ZSCAN4-low cells, from ZSCAN4-low to ZSCAN4-mid cells, from ZSCAN4-mid to ZSCAN4-high cells, and from ZSCAN4-high cells to MERVL-EGFP-positive cells. These analyses were incorporated into the revised manuscript (Fig. 3D,E). We also examined minor and major ZGA gene signatures (Fig. 3D,E).

These analyses showed that genes associated with early entry into the 2CLC program, particularly the ESC-to-ZSCAN4-low transition, remained elevated in *Ctbp1/2/Dux* TKO ESCs. In contrast, induction of the canonical minor ZGA/MERVL-high program by *Ctbp1/2* loss was abolished by *Dux* deletion (Fig. 3D,E). Thus, *Dux* deletion in the *Ctbp1/2* DKO background abolishes the GFP+++ population but does not eliminate all 2CLC-related transcriptional features. Rather, it prevents progression to the canonical MERVL-high/minor-ZGA state while allowing persistence of a lower-intensity GFP+ population associated with an early/intermediate stage of 2CLC transition.

We also considered direct PCA comparison with published 2CLC datasets. However, because the available datasets differ in experimental platform, library preparation and sample composition, cross-dataset PCA was strongly affected by batch effects and did not provide reliable biological separation. We therefore used curated 2CLC-transition gene signatures from a published trajectory as a more robust approach to assess the relationship between our mutant ESCs and previously described 2CLC states. We have revised the Results section to clarify that the *Ctbp1/2/Dux* TKO GFP+ population should not be equated with canonical MERVL-high 2CLCs, but instead represents a DUX-independent weak MERVL-EGFP-positive state with early/intermediate 2CLC-like transcriptional features.

Comment 2.

In Figure 2A, the ratio of Mervl-EGFP positive cells appears higher in Ctbp1/2/Dux TKO cells then in Ctbp1/2 DKO cells. Given that Dux is a known master activator for 2CLC state, why does its KO in Ctbp1/2 DKO background further expand the 2CLC population? In line 205-208, the authors refer to "strong" and "weak" GFP-positive populations, it would be clearer to define these subpopulations and show their exact proportions, helping readers judge whether the described changes are indeed "markedly reduced" (Line 206) or "persisted" (Line 207).

Response 2.

We thank the reviewer for raising this important point. We agree that our original description did not clearly define the “strong” and “weak” MERVL-EGFP-positive populations. In the revised manuscript, we subdivided MERVL-EGFP-positive cells into GFP+ and GFP+++ fractions using fixed fluorescence intensity gates and now show their exact proportions separately (Fig. 2A,B).

This revised analysis showed that *Ctbp1/2* DKO ESCs contained both GFP+ and GFP+++ populations, whereas the GFP+++ fraction was essentially eliminated in *Ctbp1/2/Dux* TKO ESCs (Fig. 2A,B). In contrast, the lower-intensity GFP+ fraction was expanded in *Ctbp1/2/Dux* TKO ESCs relative to *Ctbp1/2* DKO ESCs (Fig. 2A,B). Thus, the apparently higher total percentage of MERVL-EGFP-positive cells in TKO ESCs does not indicate expansion of canonical MERVL-high 2CLCs, but rather reflects accumulation of a weak MERVL-EGFP-positive population.

Our interpretation is that DUX is required for progression from the weak GFP+ state to the robust GFP+++ state. In *Ctbp1/2* DKO ESCs, a subset of GFP+ cells progresses to the DUX-dependent GFP+++ state, which is associated with reduced proliferative capacity and frequent cellular collapse. In contrast, in *Ctbp1/2/Dux* TKO ESCs, cells fail to progress to the GFP+++ state and remain in the weak GFP+ state. Because these GFP+ cells retain greater proliferative capacity than GFP+++ cells, they can accumulate over time, resulting in a higher total percentage of GFP-positive cells despite lower GFP intensity.

Consistent with this interpretation, time-lapse imaging and quantitative fluorescence analyses showed that TKO cells predominantly remained in a low-intensity GFP-positive state and rarely reached high GFP intensity (Fig. 2G–J). In addition, lineage tracking showed that DKO and TKO GFP-positive founder lineages underwent more divisions than WT GFP-positive cells (Fig. 2K). We have revised the Results section to define GFP+ and GFP+++ populations explicitly and to avoid conflating weak MERVL-EGFP positivity with the canonical MERVL-high 2CLC state.

Comment 3.

In Figure 4B, please also label the peak corresponding to Gata1-DE. Given the numerous strong Dux peaks near Pramel7 and Gata1 loci, how can the authors exclude a direct role of Dux? In Figure 4D and 4E, could be the reporter activated by Dux? Alternatively, might Dux activate Pramel7 and Gata1 by distal regulatory regions marked by Dux peaks shown in 4B?

Response 3.

We thank the reviewer for this important comment. We have added red horizontal bars indicating the distal enhancer regions selected for luciferase assays, including the *Gata1*-DE region, in the genome browser view (Fig. 5B).

We agree that DUX ChIP-seq peaks near the *Pramel7* and *Gata1* loci suggest potential DUX-dependent regulation (Fig. 5B). Indeed, the DUX-bound region within the *Pramel7* gene body increased reporter activity in luciferase assays, indicating that this sequence has transcriptional activation potential outside its endogenous chromatin context. However, short-term *Dux* induction did not activate endogenous Pramel7 expression (Fig. 3H). Thus, DUX binding to this region is not sufficient to acutely induce endogenous *Pramel7* expression in ESCs.

Importantly, *Pramel7* remained upregulated in *Ctbp1/2/Dux* TKO ESCs (Fig. 3A), and rapid-response genes including *Pramel7* and *Gata1* were derepressed by *Ctbp1/2* loss even in the *Dux* KO background (Fig. 4H). In addition, CtBP2 occupancy at the *Pramel7* and *Gata1* regulatory regions was supported by public ChIP-seq and confirmed by ChIP-qPCR (Fig. 5B,G), and these regulatory regions were derepressed in Ctbp1/2 DKO ESCs and repressed again by CtBP2 re-expression in luciferase assays (Fig. 5D,E).

These results suggest that DUX-bound sequences near *Pramel7* can show reporter activity, but endogenous *Pramel7* derepression upon *Ctbp1/2* loss is largely DUX-independent and directly constrained by CtBP2 in ESCs.

Comment 4.
The rationale for selecting Pramel7 as the downstream effector is not clearly explained in the text. Why not consider Pramel6, which shows similar expression pattern to Pramel7 (Figure 5B)? The manuscript states that expression levels of Pramel7 in Dux KO cells was "unaffected" (Line 411), yet Figure 5K shows a dramatic decrease, please clarity this discrepancy.

Response 4.

We thank the reviewer for this important comment. We agree that the rationale for focusing on PRAMEL7 should be explained more clearly. We focused on PRAMEL7 because it has an established role in early mouse development and a defined

mechanistic link to epigenetic regulation through UHRF1 degradation. This made PRAMEL7 a strong candidate mediator of the UHRF1-associated transcriptional program downstream of *Ctbp1/2* loss.

Although *Pramel6* showed a partially similar embryonic expression pattern to *Pramel7* (Fig. 6B), PRAMEL6 did not reduce UHRF1 protein levels under our experimental conditions, whereas PRAMEL7 did (Appendix Fig. S3C; Fig. 7A). This difference further supported our decision to focus on PRAMEL7.

We also agree that our original wording regarding the DUX dependency of *Pramel7* expression was unclear. Our intention was not to state that *Pramel7* is unaffected by *Dux* deletion in general. Indeed, *Pramel7* expression was markedly reduced in *Dux* KO ESCs in the WT background (Fig. 6K), indicating that basal *Pramel7* expression is at least partly DUX-dependent. In contrast, when *Ctbp1/2* are deleted, *Pramel7* remains strongly upregulated even in the absence of DUX (Fig. 3A). Thus, the term "DUX-independent" refers specifically to the persistence of *Pramel7* derepression caused by *Ctbp1/2* loss in the *Ctbp1/2/Dux* TKO background, rather than to basal *Pramel7* regulation in WT ESCs. We have revised the text to make this distinction explicit.

Comment 5.

In Line 484-485, the authors state that "downregulation of Pramel7 expression is major cause of the transcriptional abnormalities in Dux KO ESCs". Then, why does Pramel7 overexpression fail to increase the proportion of Mervl-EGFP positive cells in Dux KO cells in Figure 5F?

Response 5.

We thank the reviewer for this important comment. We agree that our original statement that reduced *Pramel7* expression is a "major" cause of the transcriptional abnormalities in *Dux* KO ESCs was too strong and potentially misleading. We have revised the manuscript to state that reduced *Pramel7* expression contributes to a subset of the transcriptional abnormalities observed in *Dux* KO ESCs.

Importantly, we do not interpret the partial transcriptional rescue by PRAMEL7 overexpression as restoration of the canonical MERVL-high 2CLC state. PRAMEL7 overexpression modestly increased the MERVL-EGFP-positive population in WT

ESCs, but failed to increase either the GFP+ or GFP+++ population in the *Dux* KO background (Fig. 6E,F). Thus, PRAMEL7 is not sufficient to restore the canonical MERVL-EGFP-positive 2CLC state in the absence of DUX.

Instead, our data suggest that PRAMEL7 influences at least two separable transcriptional outputs. One output is linked to the canonical DUX-dependent minor ZGA program: PRAMEL7 overexpression induced a cluster containing minor ZGA genes, but this induction was abolished by *Dux* deletion (Fig. 6H,I). The second output is DUX-independent and includes subsets of post-ZGA-associated and DNA methylation-sensitive genes. Consistent with this, PRAMEL7 overexpression reduced UHRF1 protein levels (Fig. 7A), was associated with DNA hypomethylation at representative loci (Fig. 7B,D,E), and partially restored a subset of genes downregulated in *Dux* KO ESCs, many of which overlapped with genes upregulated in *Dnmt1* KO ESCs (Fig. 8C,D).

Therefore, PRAMEL7 overexpression can partially rescue transcriptional defects in *Dux* KO ESCs without restoring the canonical MERVL-high 2CLC state. We have revised the text to clarify that PRAMEL7 contributes to a subset of DUX-dependent and DUX-independent transcriptional programs, but is not sufficient to account for the loss of the canonical MERVL-EGFP-positive 2CLC population in *Dux* KO ESCs.

Comment 6.

The authors conclude that Pramel7 activation by the loss of Ctbp1/2 "regulates maternally, major ZGA and post-ZGA genes through modulation of DNA methylation mediated by UHRF1 degradation in 2CLCs" (Line 502-503). First, does Pramel7 KO in Ctbp1/2 DKO mESC reduce the proportion of Mervl-EGFP positive cells? What about in Ctbp1/2/Dux TKO line? Second, the DNA demethylation in 2CLCs induced by Ctbp1/2 DKO should be examined, at least at several key loci, such as Dux locus.

Response 6.

We thank the reviewer for this important comment. We agree that our original statement was too definitive, and we have revised the manuscript to state that PRAMEL7 contributes to DNA demethylation-linked transcriptional regulation, rather than claiming that PRAMEL7 alone regulates maternal, major ZGA and post-ZGA genes in 2CLCs.

With respect to the requirement for *Pramel7* in the MERVL-EGFP-positive population, we generated *Ctbp1/2/Dux/Pramel7* quadruple-knockout ESCs and examined the residual MERVL-EGFP-positive population. Loss of *Pramel7* in the *Ctbp1/2/Dux*-deficient background did not abolish the residual weak MERVL-EGFP-positive population. This observation suggests that PRAMEL7 is not the sole determinant of the DUX-independent MERVL-EGFP-positive state. Consistent with this interpretation, PRAMEL7 overexpression failed to generate either GFP+ or GFP+++ cells in the Dux KO background (Fig. 6E,F). Thus, PRAMEL7 alone is neither sufficient nor solely required for DUX-independent MERVL-EGFP activation.

One possible explanation is functional redundancy among PRAME family members. Indeed, multiple Prame family genes are upregulated upon *Ctbp1/2* loss (Fig. 6A), including DUX-independent members such as Pramel7, Pramel14 and Pramel30. In our preliminary analysis, we found that PRAMEL14 and PRAMEL30, like PRAMEL7, can reduce UHRF1 protein levels, suggesting that these PRAME family members may have partially redundant activities. Therefore, complete evaluation of PRAME family function in the residual MERVL-EGFP-positive population will require combined perturbation of *Pramel7, Pramel14* and *Pramel30*, which we are planning for future studies and which is beyond the scope of the present revision. We have revised the manuscript to avoid implying that PRAMEL7 alone accounts for the MERVL-EGFP-positive phenotype induced by *Ctbp1/2* loss.

Regarding DNA methylation, we performed bisulfite sequencing at the *Duxf3* locus in WT and *Ctbp1/2* DKO ESCs. *Ctbp1/2* DKO ESCs showed reduced DNA methylation at this locus compared with WT ESCs (Fig. 7G). In addition, UHRF1 protein levels were reduced in *Ctbp1/2* DKO and *Ctbp1/2/Dux* TKO ESCs (Fig. 7F), and *Pramel7* KO impaired UHRF1 downregulation in ZSCAN4-positive cells without completely abolishing it (Fig. 8E,F). Together, these data support the idea that PRAMEL7 contributes to UHRF1-associated DNA demethylation-linked regulation, while also suggesting that additional PRAME family members, particularly PRAMEL14 and PRAMEL30, may act redundantly.

Comment 7.

Based on presented data, the relationship between CtBP1/2, Dux, and Pramel7 remains unclear. RNA-seq overlap analysis provides correlation but not causation. Performing Dux ChIP-seq in Ctbp1/2 DKO, Pramel7 KO, and Ctbp1/2/Pramel7 TKO cells would

provide necessary information to elucidate the regulatory interactions among CtBP1/2, Dux, and Pramel7 in mESC.

Response 7.

We thank the reviewer for this thoughtful suggestion. We agree that DUX ChIP-seq in additional mutant backgrounds, including *Ctbp1/2* DKO, *Pramel7* KO and *Ctbp1/2/Pramel7* mutant ESCs, would provide useful information regarding DUX occupancy and could further clarify direct versus indirect DUX-dependent regulation.

However, the central question addressed in the present study is whether transcriptional programs induced by *Ctbp1/2* loss and PRAMEL7 activation are DUX-dependent or DUX-independent. To address this, we performed genetic comparisons among WT, *Dux* KO, *Ctbp1/2* DKO and *Ctbp1/2/Dux* TKO ESCs. These analyses showed that the canonical minor ZGA/MERVL-high program is DUX-dependent, whereas a subset of major ZGA- and post-ZGA-associated genes remains induced in the absence of DUX (Fig. 3A–H). In addition, CtBP2 occupancy and ChIP-qPCR analyses support direct repression of *Pramel7* and *Gata1* by CtBP2 (Fig. 5B,G), and enhancer reporter assays showed that these regulatory regions are derepressed in *Ctbp1/2* DKO ESCs and repressed again by CtBP2 re-expression (Fig. 5D,E).

Regarding PRAMEL7, PRAMEL7 overexpression failed to restore the MERVL-EGFP-positive population in *Dux* KO ESCs (Fig. 6E,F), indicating that PRAMEL7 is not sufficient to activate the canonical MERVL reporter program without DUX. In contrast, PRAMEL7 overexpression restored a subset of genes downregulated in *Dux* KO ESCs, many of which overlapped with genes upregulated in *Dnmt1* KO ESCs (Fig. 8C,D). These results suggest that PRAMEL7 contributes to a DUX-independent, DNA methylation-linked transcriptional program, while the canonical MERVL-high/minor-ZGA program remains DUX-dependent.

Thus, although DUX ChIP-seq in additional mutant backgrounds would be informative and may be important in future studies, we believe that the genetic, CtBP2 ChIP-qPCR, enhancer reporter and PRAMEL7 rescue analyses presented here directly address the main mechanistic question of this revision: how CtBP1/2 constrains both DUX-dependent and DUX-independent transcriptional programs in ESCs. We have revised the manuscript to clarify this point and to avoid overinterpreting RNA-seq overlap analysis as direct evidence of causality.

Comment 8.

Figure 1, a heatmap showing the expression of minor ZGA genes in WT and DKO cell lines would improve clarity.

Response 8.

We thank the reviewer for this helpful suggestion. In the revised manuscript, we added heatmap and violin plot analyses to visualize the expression of representative minor ZGA genes and minor ZGA-associated signatures across WT, *Dux* KO, *Ctbp1/2* DKO and *Ctbp1/2/Dux* TKO ESCs (Fig. 3D,E). These analyses show that minor ZGA-associated genes are induced upon loss of *Ctbp1/2*, but that this induction is largely abolished by *Dux* deletion (Fig. 3D,E). Thus, the revised presentation clarifies both the upregulation of minor ZGA genes in *Ctbp1/2* DKO ESCs and their dependency on DUX.

Comment 9.

Ensure consistency in terminology, use either "RT-qPCR" or "qRT-PCR".

Response 9.

We thank the reviewer for pointing this out. We have revised the manuscript to use RT-qPCR consistently throughout the text, Methods and figure legends.

Comment 10.

Please add scale bars to the images in Figure 4F and 5E.

Response 10.

We thank the reviewer for pointing this out. We have added scale bars to the relevant fluorescence and immunofluorescence images in the revised figures, including the MERVL-EGFP images and immunofluorescence panels, and updated the corresponding figure legends.

Comment 11.

In line 209, the authors cite Guo et al., 2019, but that study did not report the expression levels of Pramel7. If the authors used their data, please include a panel showing Pramel7 expression levels in the present study.

Response 11.

We thank the reviewer for this important comment. We agree that Guo et al. (2019) did not explicitly report *Pramel7* expression. To address this point, we re-analyzed the published embryo RNA-seq dataset and added a new panel showing *Pramel7* expression levels in WT and *Dux* KO embryos (Appendix Fig. S2A). This analysis showed that *Pramel7* is upregulated at the 2-cell stage in both WT and *Dux* KO embryos (Appendix Fig. S2A). We also revised the corresponding sentence in the Results section to clarify that this conclusion is based on our re-analysis of the published dataset, rather than on direct reporting in Guo et al. (2019).

1st Revision - Editorial Decision 19th Jun 2026

Dear Dr. Seki,

Thank you for the submission of your revised manuscript to our editorial offices. I have now received the reports from the three referees that were asked to re-evaluate the study, you will find below. As you will see, the referees now support publication of your study in EMBO reports. Referees #1 and #2 have remaining concerns or suggestions to improve the manuscript, I ask you to address in a final revised manuscript.

Moreover, I have the editorial requests below. Please also provide a final p-b-p-response to the remaining referee concerns and the editorial requests.

Editorial requests:

- Please provide the abstract written in present tense.

- Please add up to 5 keywords to the manuscript, placed below the abstract.

- Please check again that the number "n" for how many independent experiments were performed, their nature (biological versus technical replicates), the bars and error bars (e.g. SEM, SD) and the test used to calculate p-values is indicated in the respective figure legends (main and EV figures). Please also check that all the p-values are explained in the legend, and that these fit to those shown in the figure. Please provide statistical testing where applicable. Please avoid the phrase 'independent experiment' but clearly state if these were biological or technical replicates. Please also indicate (e.g. with n.s.) if testing was performed, but the differences are not significant. In case n=2, please show the data as separate datapoints without error bars and statistics. See also:

https://link.springer.com/journal/44319/submission-guidelines#cms-Figure-and-data-presentation

If n<5, please show single datapoints for diagrams. Presently, several diagrams (e.g. 2E, 3A, 3H, 5D-G, 8G and S2 D,E,I) miss statistics or show only partial statistics. It seems also "n.s." is mostly not indicated. Moreover:
Please define the annotated p values ****/***/**/* as well as provide the exact p-values for the same in the legend of figures 4H; 5D,E,F; Appendix Figure S2-I; as appropriate.
- Please note that the exact p values are not provided in the legends of 2B,I,J; 3H; 4A; 6J,K; 8F.
- Please indicate the statistical test used for data analysis in the legends of figures 1A,B; 8F.
- Please note that for the figure 3A, p-values and statistical tests are indicated in the legends. However, comparison for the same, ""****/***/**/*"" has not been represented in the figures. Please rectify this in the figures or legends as applicable.
- Please note that the box plots need to be defined in terms of minima, maxima, centre, bounds of box and whiskers, and percentile in the legends of figures 3D; 4H; 7D; 8F.
- Please note that information related to n is missing in the legends of Appendix Figure S2-B,D,E,F,G,H,I,J.
- Please note that the measure of center for the error bars needs to be defined in the legends of figures Appendix Figure S2-B,D,E,I,J.
- Please note that the scale bar needs to be defined for figure 2G.

- Table EV1 is a dataset. Please name this file Dataset EV1 and upload it as dataset file. Please adjust all the callouts (Dataset EV1).

- Please move the primer and oligo information presently part of Dataset EV1 to the reagents and tools table and remove the TAB from the dataset. Please update the callouts and also add further callouts to the Reagents & Tools Table where appropriate.

- Please add scale bars of similar style and thickness to microscopic images, using clearly visible black or white bars (depending on the background). Please place these in the lower right corner of the images themselves. Please do not write on or near the bars in the image but define the size in the respective figure legend. Presently, most scale bars are too thin. Please provide thicker scale bars.

- Please make sure each figure panel is called out sequentially and separately. Presently, separate callouts for panels 7C,D,E seem missing. Please check.

- Please remove the legends for the movies from the manuscript text file. Please provide separate legends for each movie as text file ZIPped up with its respective movie file so that we have 4 zip folders (Movie EV1 - Movie EV4).

- Please add a title page to the Appendix stating "Appendix for [Ms Title]" (do not add authors names or affiliations) and a table of contents (ToC) with each figure listed with its page number.

- Thanks for providing the source data. Please check again that this is complete. It seems numerical data for 5I,J,K are missing. In case, please add the respective file to the folder for Fig. 5.

In addition, I would need from you uploaded separately:
- a short, two-sentence summary of the manuscript (not more than 35 words).
- two to four short (!) bullet points highlighting the key findings of your study (two lines each).
- a schematic summary figure as separate file that provides a sketch of the major findings (not a data image) in jpeg or tiff format (with the exact width of 550 pixels and the exact height of 300 pixels) that can be used as a visual synopsis on our website. Please upload this separately and remove the graphical abstract from the manuscript text file.

I look forward to seeing the further revised version of your manuscript when it is ready. Please let me know if you have questions regarding the revision.

Best,

Achim Breiling
Senior Editor
EMBO Reports

Referee #1:

In the revised manuscripts, the authors have addressed most of my previous concerns. The statements in the paper are much more accurate and better reflect the actual data. I support the publication of the paper, provided that the following questions are appropriately addressed.

1. Since there are specific genes with defined functions that were up-regulated genes in CtBP1/2 DKO cells, can the authors show global analysis to examine whether the derepressed genes were preferentially CtBP1/2 binding targets, hence the specificity?

2. Overexpression of Dppa3, which drives global DNA demethylation, was shown to activate 2-cell like programs in mESCs (Zhang et al., Front. Cell Dev. Biol. 2022). PRAMEL15 was also shown to promote degradation of DNMT1 (Tan et al., Nat. Comm. 2024). The authors are advised to discuss the previous studies when describing mechanisms proposed by the authors, the PRAME protein-DNA methylation-2C-like activation axis.

Referee #2:

The authors have made considerable efforts to improve the manuscript and incorporate the suggestions raised in the previous submission. The story is cleaner and less fragmented, and they have carefully addressed each concern that was raised. Some further technical concerns about the content and presentation of the manuscript are indicated below.

It is peculiar for the authors not to cite the paper of Sakashita et al (Nature Genetics 2023) for the potential 2C gene regulation by MERVL in Introduction (and its involvement in the transition from totipotent to pluripotent state in mouse early embryos).

As Referee #1 raised in Comment 3, the authors should perform chimera formation assays (blastocyst injection assays) to examine if Ctbp1/2/Dux TKO MERVL positive mECSs could contribute to both embryonic and extraembryonic cell lineages in my opinion (though I myself did not requested the authors to do so in the 1st review).

Referee #3:

My previous concerns have been adequately addressed. I've no more criticisms.

Referee #1

Comment 1

Since there are specific genes with defined functions that were up-regulated genes in CtBP1/2 DKO cells, can the authors show global analysis to examine whether the derepressed genes were preferentially CtBP1/2 binding targets, hence the specificity?

Response 1

We thank the reviewer for this important suggestion. We performed a global analysis using publicly available CtBP2 ChIP-seq data and assigned CtBP2-binding peaks to nearby genes using GREAT with the “two nearest genes” rule. This analysis showed that genes upregulated in *Ctbp1/2* DKO ESCs were significantly enriched for CtBP2-associated genes compared with the RNA-seq background. We have added this analysis as Appendix Figure S2G and described the result in the revised manuscript. These data support the specificity of the transcriptional derepression induced by *Ctbp1/2* loss.

Comment 2

Overexpression of Dppa3, which drives global DNA demethylation, was shown to activate 2-cell like programs in mESCs (Zhang et al., Front. Cell Dev. Biol. 2022). PRAMEL15 was also shown to promote degradation of DNMT1 (Tan et al., Nat. Comm. 2024). The authors are advised to discuss the previous studies when describing mechanisms proposed by the authors, the PRAME protein-DNA methylation-2C-like activation axis.

Response 2

We thank the reviewer for pointing out these relevant studies. We have revised the Discussion to include previous reports showing that DPPA3 overexpression induces DNA hypomethylation and promotes 2C-like transcriptional programs in mESCs, and that maternal PRAMEL15 promotes nuclear DNMT1 degradation and DNA demethylation in two-cell embryos. We have also clarified that, although PRAMEL15 functions in early embryos, *Pramel15* was not detectably expressed in published RNA-seq datasets of ESC-derived 2CLCs and was not upregulated in our *Ctbp1/2* DKO ESCs, suggesting that PRAMEL15 is unlikely to mediate the transcriptional effects observed in our ESC-based system. These additions place our findings in the broader context of DNA methylation-dependent regulation of early embryo-associated transcriptional programs, while clarifying that our data identify PRAMEL7–UHRF1 regulation as the relevant axis downstream of *Ctbp1/2* loss in ESCs.

Referee #2

Comment 1:

It is peculiar for the authors not to cite the paper of Sakashita et al. (Nature Genetics 2023) for the potential 2C gene regulation by MERVL in Introduction and its involvement in the transition from totipotent to pluripotent state in mouse early embryos.

Response 1:

We thank the reviewer for this important suggestion. We have revised the Introduction to cite Sakashita et al. and to describe their finding that MERVL transcription is required for preimplantation embryo development and contributes to the regulation of two-cell-stage gene expression and the totipotent-to-pluripotent transition in early mouse embryos. This addition clarifies the biological relevance of MERVL beyond its use as a marker of 2C-like cells.

Comment 2:

As Referee #1 raised in Comment 3, the authors should perform chimera formation assays, blastocyst injection assays, to examine if *Ctbp1/2/Dux* TKO MERVL-positive mESCs could contribute to both embryonic and extraembryonic cell lineages in my opinion, though I myself did not request the authors to do so in the first review.

Response 2: We appreciate the reviewer's insightful suggestion. We agree that blastocyst injection and chimera formation assays would be important future experiments to directly evaluate the developmental potential of Ctbp1/2/Dux TKO MERVL-EGFP-positive ESCs. However, the central aim of the present study is to define transcriptional programs derepressed by *Ctbp1/2* loss and to distinguish DUX-dependent and DUX-independent regulatory pathways in ESCs. We do not claim that *Ctbp1/2/Dux* TKO MERVL-EGFP-positive cells acquire bona fide totipotency or extraembryonic lineage potential. To clarify this limitation, we have revised the Discussion to state that although *Ctbp1/2/Dux* TKO ESCs retain a MERVL-EGFP-positive population, our data do not establish bona fide totipotency or extraembryonic lineage potential, and that future blastocyst injection and chimera formation assays will be required to determine the developmental potential of these cells.

Referee #3

Comment: My previous concerns have been adequately addressed. I have no more criticisms.

Response: We thank the reviewer for the positive evaluation of our revised manuscript.

Dr. Yoshiyuki Seki
Kwansei Gakuin University
School of Science and Technology
2-1 Gakuen
Sanda, Hyogo 669-1337
Japan

Dear Dr. Seki,

Thank you for the submission of your further revised manuscript to our editorial offices. I now went through this and your final p-b-p-response and consider the remaining points of the referees and the editorial requests as adequately addressed.

I am thus very pleased to accept your manuscript for publication in the next available issue of EMBO reports. Thank you for your contribution to our journal.

Your manuscript will be processed for publication by EMBO Press. It will be copy edited and you will receive page proofs prior to publication. Please note that you will be contacted by Springer Nature Author Services to complete licensing and payment information.

You may qualify for financial assistance for your publication charges - either via a Springer Nature fully open access agreement or an EMBO initiative. Check your eligibility: https://link.springer.com/journal/44319/how-to-publish-with-us

Should you be planning a Press Release on your article, please notify the production team via this form in order to coordinate publication and release dates: https://forms.cloud.microsoft/e/eH4A8JVP6g

If you have any questions, please do not hesitate to contact the Editorial Office. Thank you for your contribution to EMBO Reports.

Yours sincerely,

Achim Breiling
Senior Editor
EMBO Reports

>>> Please note that it is EMBO Reports policy for the transcript of the editorial process (containing referee reports and your response letter) to be published as an online supplement to each paper. If you do NOT want this, you will need to inform the Editorial Office via email immediately. More information is available here: https://link.springer.com/partners/embo-press/editorial-policies#Peer%20review

EMBO Press Author Checklist

Corresponding Author Name: Yoshiyuki Seki	
Journal Submitted to: EMBO Reports	
Manuscript Number: EMBOR-2025-63169V1	

USEFUL LINKS FOR COMPLETING THIS FORM
The EMBO Journal - Author Guidelines
EMBO Reports - Author Guidelines
Molecular Systems Biology - Author Guidelines
EMBO Molecular Medicine - Author Guidelines

Reporting Checklist for Life Science Articles (updated January
This checklist is adapted from Materials Design Analysis Reporting (MDAR) Checklist for Authors. MDAR establishes a minimum set of requirements in transparent reporting in the life sciences (see Statement of Task: 10.31222/osf.io/9sm4x). Please follow the journal's guidelines in preparing your
Please note that a copy of this checklist will be published alongside your article.

Abridged guidelines for figures
1. Data
The data shown in figures should satisfy the following conditions:
- the data were obtained and processed according to the field's best practice and are presented to reflect the results of the experiments in an accurate and unbiased manner.
- ideally, figure panels should include only measurements that are directly comparable to each other and obtained with the same assay.
- plots include clearly labeled error bars for independent experiments and sample sizes. Unless justified, error bars should not be shown for technical
- if n<5, the individual data points from each experiment should be plotted. Any statistical test employed should be justified.
- Source Data should be included to report the data underlying figures according to the guidelines set out in the authorship guidelines on Data

2. Captions
Each figure caption should contain the following information, for each panel where they are relevant:
- a specification of the experimental system investigated (eg cell line, species name).
- the assay(s) and method(s) used to carry out the reported observations and measurements.
- an explicit mention of the biological and chemical entity(ies) that are being measured.
- an explicit mention of the biological and chemical entity(ies) that are altered/varied/perturbed in a controlled manner.
- the exact sample size (n) for each experimental group/condition, given as a number, not a range;
- a description of the sample collection allowing the reader to understand whether the samples represent technical or biological replicates (including how many animals, litters, cultures, etc.).
- a statement of how many times the experiment shown was independently replicated in the laboratory.
- definitions of statistical methods and measures:
  - common tests, such as t-test (please specify whether paired vs. unpaired), simple χ2 tests, Wilcoxon and Mann-Whitney tests, can be unambiguously identified by name only, but more complex techniques should be described in the methods section;
  - are tests one-sided or two-sided?
  - are there adjustments for multiple comparisons?
  - exact statistical test results, e.g., P values = x but not P values < x;
  - definition of 'center values' as median or average;
  - definition of error bars as s.d. or s.e.m.

Please complete ALL of the questions below.
Select "Not Applicable" only when the requested information is not relevant for your study.

Materials

Newly Created Materials	Information included in the manuscript?	In which section is the information available? (Reagents and Tools Table, Materials and Methods, Figures, Data Availability Section)
New materials and reagents need to be available; do any restrictions apply?	Yes	Reagent and Tools Table

Antibodies	Information included in the manuscript?	In which section is the information available? (Reagents and Tools Table, Materials and Methods, Figures, Data Availability Section)
For **antibodies** provide the following information:<br>- Commercial antibodies: RRID (if possible) or supplier name, catalogue number and or/clone number<br>- Non-commercial: RRID or citation	Yes	Reagent and Tools Table, Materials and Methods

DNA and RNA sequences	Information included in the manuscript?	In which section is the information available? (Reagents and Tools Table, Materials and Methods, Figures, Data Availability Section)
**Short novel DNA or RNA including primers, probes**: provide the sequences.	Yes	Supplementary Table

Cell materials	Information included in the manuscript?	In which section is the information available? (Reagents and Tools Table, Materials and Methods, Figures, Data Availability Section)
**Cell lines:** Provide species information, strain. Provide accession number in repository **OR** supplier name, catalog number, clone number, and/**OR** RRID.	Yes	Reagent and Tools Table
**Primary cultures:** Provide species, strain, sex of origin, genetic modification status.	Not Applicable	
Report if the cell lines were recently **authenticated** (e.g., by STR profiling) and tested for mycoplasma contamination.	Yes	Cell lines were not recently authenticated by STR profiling or tested for mycoplasma contamination. Genetically modified ESC lines were validated by genotyping PCR and/or reporter or transgene expression where

Experimental animals	Information included in the manuscript?	In which section is the information available? (Reagents and Tools Table, Materials and Methods, Figures, Data Availability Section)
**Laboratory animals or Model organisms:** Provide species, strain, sex, age, genetic modification status. Provide accession number in repository **OR** supplier name, catalog number, clone number, **OR** RRID.	Not Applicable	
**Animal observed in or captured from the field:** Provide species, sex, and age where possible.	Not Applicable	
Please detail **housing and husbandry conditions**.	Not Applicable	

Plants and microbes	Information included in the manuscript?	In which section is the information available? (Reagents and Tools Table, Materials and Methods, Figures, Data Availability Section)
**Plants:** provide species and strain, ecotype and cultivar where relevant, unique accession number if available, and source (including location for collected wild specimens).	Not Applicable	
**Microbes:** provide species and strain, unique accession number if available, and source.	Not Applicable	

Human research participants	Information included in the manuscript?	In which section is the information available? (Reagents and Tools Table, Materials and Methods, Figures, Data Availability Section)
If collected and within the bounds of privacy constraints report on age, sex and gender or ethnicity for all study participants.	Not Applicable	

Core facilities	Information included in the manuscript?	In which section is the information available? (Reagents and Tools Table, Materials and Methods, Figures, Data Availability Section)
If your work benefited from core facilities, was their service mentioned in the acknowledgments section?	Not Applicable	

Design

Study protocol	Information included in the manuscript?	In which section is the information available? (Reagents and Tools Table, Materials and Methods, Figures, Data Availability Section)
If study protocol has been **pre-registered, provide DOI in the manuscript**. For clinical trials, provide the trial registration number **OR** cite DOI.	Not Applicable	
Report the **clinical trial registration number** (at ClinicalTrials.gov or equivalent), where applicable.	Not Applicable	

Laboratory protocol	Information included in the manuscript?	In which section is the information available? (Reagents and Tools Table, Materials and Methods, Figures, Data Availability Section)
Provide DOI OR other citation details if **external detailed step-by-step protocols** are available.	Yes	

Experimental study design and statistics	Information included in the manuscript?	In which section is the information available? (Reagents and Tools Table, Materials and Methods, Figures, Data Availability Section)
Include a statement about **sample size** estimate even if no statistical methods were used.	Yes	Figure legends
Were any steps taken to minimize the effects of subjective bias when allocating animals/samples to treatment (e.g. **randomization procedure**)? If yes, have they been described?	Not Applicable	
Include a statement about **blinding** even if no blinding was done.	Yes	Blinding was not performed during data acquisition or analysis.
Describe **inclusion/exclusion criteria** if samples or animals were excluded from the analysis. Were the criteria pre-established?<br><br>If sample or data points were omitted from analysis, report if this was due to attrition or intentional exclusion and provide justification.	Not Applicable	
For every figure, are **statistical tests** justified as appropriate? Do the data meet the assumptions of the tests (e.g., normal distribution)? Describe any methods used to assess it. Is there an estimate of variation within each group of data? Is the variance similar between the groups that are being statistically compared?	Yes	Figure legends

Sample definition and in-laboratory replication	Information included in the manuscript?	In which section is the information available? (Reagents and Tools Table, Materials and Methods, Figures, Data Availability Section)
In the figure legends: state number of times the experiment was **replicated** in laboratory.	Yes	Figure legends
In the figure legends: define whether data describe **technical or biological replicates**.	Yes	Figure legends

Ethics

Ethics	Information included in the manuscript?	In which section is the information available? (Reagents and Tools Table, Materials and Methods, Figures, Data Availability Section)
Studies involving **human participants**: State details of **authority granting ethics approval** (IRB or equivalent committee(s), provide reference number for approval.	Not Applicable	
Studies involving **human participants**: Include a statement confirming that **informed consent** was obtained from all subjects and that the experiments conformed to the principles set out in the WMA Declaration of Helsinki and the Department of Health and Human Services Belmont Report.	Not Applicable	
Studies involving **human participants:** For publication of **patient photos**, include a statement confirming that consent to publish was obtained.	Not Applicable	
Studies involving experimental **animals**: State details of **authority granting ethics approval** (IRB or equivalent committee(s), provide reference number for approval. Include a statement of compliance with ethical regulations.	Not Applicable	
Studies involving **specimen and field samples:** State if relevant **permits** obtained, provide details of authority approving study; if none were required, explain why.	Not Applicable	

Dual Use Research of Concern (DURC)	Information included in the manuscript?	In which section is the information available? (Reagents and Tools Table, Materials and Methods, Figures, Data Availability Section)
Could your study fall under dual use research restrictions? Please check biosecurity documents and list of **select agents and toxins** (CDC): https://www.selectagents.gov/sat/list.htm	Not Applicable	
If you used a select agent, is the security level of the lab appropriate and reported in the manuscript?	Not Applicable	
If a study is subject to dual use research of concern regulations, is the name of the **authority granting approval and reference number** for the regulatory approval provided in the manuscript?	Not Applicable	

Reporting

The MDAR framework recommends adoption of discipline-specific guidelines, established and endorsed through community initiatives. Journals have their own policy about requiring specific guidelines and recommendations to complement MDAR.

Adherence to community standards	Information included in the manuscript?	In which section is the information available? (Reagents and Tools Table, Materials and Methods, Figures, Data Availability Section)
State if relevant guidelines or checklists (e.g., **ICMJE, MIBBI, ARRIVE, PRISMA**) have been followed or provided.	Not Applicable	
For **tumor marker prognostic studies,** we recommend that you follow the **REMARK** reporting guidelines (see link list at top right). See author guidelines, under 'Reporting Guidelines'. Please confirm you have followed these guidelines.	Not Applicable	
For **phase II and III randomized controlled trials**, please refer to the **CONSORT** flow diagram (see link list at top right) and submit the CONSORT checklist (see link list at top right) with your submission. See author guidelines, under 'Reporting Guidelines'. Please confirm you have submitted this list.	Not Applicable	

Data Availability

Data availability	Information included in the manuscript?	In which section is the information available? (Reagents and Tools Table, Materials and Methods, Figures, Data Availability Section)
Have **primary datasets** been deposited according to the journal's guidelines (see 'Data Deposition' section) and the respective accession numbers provided in the Data Availability Section?	Yes	Data Availability Section
Were **human clinical and genomic datasets** deposited in a public access-controlled repository in accordance to ethical obligations to the patients and to the applicable consent agreement?	Not Applicable	
Are **computational models** that are central and integral to a study available without restrictions in a machine-readable form? Were the relevant accession numbers or links provided?	Not Applicable	
If publicly available data were reused, provide the respective **data citations in the reference list**.	Yes	Data Availability Section
