# Supplementary material for: CtBP1/2 restrict DUX-dependent and independent 2C-like programs in mouse embryonic stem cells
Source: EMBO Rep. 2026 Aug 3;27(18):5317–40. doi: 10.1038/s44319-026-00881-7 (PMC13601577; doi:10.1038/s44319-026-00881-7)
Supplement: Supplementary file 3 [file 44319_2026_881_MOESM10_ESM.zip › Figure_3_source_data/Panel_B/README.docx]

**README**

Source data for Figure 3B

File included

DKO_WTvsTKO_DuxKO.xlsx

Description

This file contains the gene-level log2 fold-change values used to generate the scatter plot shown in Figure 3B. Figure 3B compares transcriptional changes induced by Ctbp1/2 loss in the WT background with those induced by Ctbp1/2 loss in the Dux KO background.

DKO_WTvsTKO_DuxKO.xlsx

Each row represents one gene.

Columns

- symbol: Gene symbol or gene identifier.

- DKO_WT: log2 fold change calculated as Ctbp1/2 DKO ESCs minus WT ESCs.

- TKO_DuxKO: log2 fold change calculated as Ctbp1/2/Dux TKO ESCs minus Dux KO ESCs.

Plotting

Each point in Figure 3B represents one gene. The x-axis shows TKO_DuxKO log2 fold changes, and the y-axis shows DKO_WT log2 fold changes. Selected genes and developmental gene categories are labeled in the figure.

Correlation

The Pearson correlation coefficient between DKO_WT and TKO_DuxKO log2 fold changes is shown in the figure.

Statistical analysis

No separate statistical test was performed for Figure 3B because this panel shows a gene-level scatter plot comparing RNA-seq log2 fold changes between two genotype contrasts. The differential-expression statistics for the underlying RNA-seq comparisons are provided in the corresponding RNA-seq source data files.

Notes

Genes upregulated in both Ctbp1/2 DKO versus WT and Ctbp1/2/Dux TKO versus Dux KO represent DUX-independent genes induced by Ctbp1/2 loss.
