# Supplementary material for: CtBP1/2 restrict DUX-dependent and independent 2C-like programs in mouse embryonic stem cells
Source: EMBO Rep. 2026 Aug 3;27(18):5317–40. doi: 10.1038/s44319-026-00881-7 (PMC13601577; doi:10.1038/s44319-026-00881-7)
Supplement: Supplementary file 3 [file 44319_2026_881_MOESM10_ESM.zip › Figure_3_source_data/Panel_D_E/README.docx]

**README**

Source data for Figure 3D and 3E

This folder contains the source data used to generate Figure 3D and 3E. Panels D and E were generated from the same normalized expression dataset and related signature gene sets.

Included files:

1. Geneset_Figure_3C.xlsx

Gene sets used for the signature analyses shown in Figure 3D and for the selected genes displayed in Figure 3E.

2. WT_DuxKO_DKO_TKO_Log2CPM.xlsx

Normalized expression matrix (log2CPM) for WT, Dux KO, Ctbp1/2 DKO and Ctbp1/2/Dux TKO samples.

3. ZGA_2CLC_groupmean_geneZ_long.csv

Long-format table containing gene-wise Z-scores used for the violin plots in Figure 3D. This file includes gene name, genotype group, Z-score and signature annotation.

4. Selected_heatmap_geneZ_matrix_specified.csv

Z-score matrix used to generate the heatmaps in Figure 3E for the selected representative genes.

Data processing:

Gene-wise Z-scores were calculated from the normalized log2CPM expression matrix using the corresponding gene sets. Figure 3D shows the distribution of gene-wise Z-scores for each signature across genotypes. Figure 3E shows heatmaps of selected representative genes using the same Z-score framework.

Sample groups:

WT, Dux KO, Ctbp1/2 DKO and Ctbp1/2/Dux TKO.

Statistical analysis:

No statistical test was performed for these panels because they show gene-wise Z-score distributions and representative heatmaps derived from RNA-seq expression data, rather than replicate-based quantitative measurements. Differential-expression statistics for the underlying RNA-seq data are provided in the corresponding RNA-seq source data files.
