# Supplementary material for: CtBP1/2 restrict DUX-dependent and independent 2C-like programs in mouse embryonic stem cells
Source: EMBO Rep. 2026 Aug 3;27(18):5317–40. doi: 10.1038/s44319-026-00881-7 (PMC13601577; doi:10.1038/s44319-026-00881-7)
Supplement: Supplementary file 3 [file 44319_2026_881_MOESM10_ESM.zip › Figure_3_source_data/Panel_G/README.docx]

**README**

Source data and enrichment statistics for Figure 3G

Files included

1. cluster1_5_geneSet_enrichment_stats.csv

2. Geneset.csv

3. WT_DuxKO_DKO_TKO_Clustering.csv

Description

These files contain the source data and enrichment statistics used to generate the bubble plot shown in Figure 3G. Figure 3G shows enrichment analysis of selected developmental and control gene sets across clusters 1–5 identified by K-means clustering in Figure 3F.

cluster1_5_geneSet_enrichment_stats.csv

This file contains the gene set enrichment statistics used to generate the bubble plot in Figure 3G.

Columns

- cluster: K-means cluster number.

- gene_set: Gene set or signature tested for enrichment.

- overlap: Number of genes overlapping between the cluster and the gene set.

- expected_overlap: Expected number of overlapping genes.

- observed_expected_ratio: Observed/expected overlap ratio used for dot size.

- p_value: Nominal P value from the enrichment test.

- FDR: Multiple-testing-adjusted P value.

- minus_log10_FDR: -log10(FDR) value used for dot color.

Geneset.csv

This file contains the gene sets used for enrichment analysis. Gene sets include:

- Maternal

- Minor ZGA

- Major ZGA

- Naïve

- Formative

- Committed

- TE

- PrE

- p53

- Housekeeping

WT_DuxKO_DKO_TKO_Clustering.csv

This file contains the clustered gene expression matrix used as the basis for the enrichment analysis. The file includes gene symbols, normalized expression values for WT, Dux KO, Ctbp1/2 DKO and Ctbp1/2/Dux TKO ESC samples, and the assigned K-means cluster for each gene.

Cluster sizes

- Cluster 1: N = 184 genes

- Cluster 2: N = 632 genes

- Cluster 3: N = 334 genes

- Cluster 4: N = 577 genes

- Cluster 5: N = 273 genes

Data processing

Genes were assigned to clusters 1–5 based on the K-means clustering analysis shown in Figure 3F. Enrichment of each gene set in each cluster was then calculated by comparing the observed overlap with the expected overlap. The observed/expected ratio was used for dot size, and -log10(FDR) was used for dot color in Figure 3G.

Statistical analysis

Gene set enrichment was assessed for each cluster and gene set using an enrichment test, followed by multiple-testing correction to calculate FDR values. Exact nominal P values and FDR values are provided in cluster1_5_geneSet_enrichment_stats.csv.

Notes

No additional statistical test was performed on the plotted bubble values because Figure 3G directly visualizes the enrichment statistics provided in cluster1_5_geneSet_enrichment_stats.csv.
