# Supplementary material for: CtBP1/2 restrict DUX-dependent and independent 2C-like programs in mouse embryonic stem cells
Source: EMBO Rep. 2026 Aug 3;27(18):5317–40. doi: 10.1038/s44319-026-00881-7 (PMC13601577; doi:10.1038/s44319-026-00881-7)
Supplement: Supplementary file 3 [file 44319_2026_881_MOESM10_ESM.zip › Figure_3_source_data/Panel_A/README.docx]

**README**

Source data and statistical analysis for Figure 3A

Files included

1. WT_DuxKO_DKO_TKO_qRT-PCR.csv

2. Figure3A_qPCR_TukeyHSD_all_comparisons.csv

Description

These files contain the source data and statistical analysis used to generate the RT-qPCR plots shown in Figure 3A. Figure 3A shows the expression of Dux, Zscan4c and Pramel7 in WT, Dux KO, Ctbp1/2 DKO and Ctbp1/2/Dux TKO ESCs.

WT_DuxKO_DKO_TKO_qRT-PCR.csv

This file contains the qRT-PCR values used for plotting. Only the Dux, Zscan4c and Pramel7 entries are used for Figure 3A.

Columns

- Cell: Cell type or genotype analyzed.

- Gene: Gene analyzed by qRT-PCR.

- Ct: qRT-PCR value used for analysis and plotting. Blank values indicate conditions in which the corresponding target was not detected or not applicable because of gene deletion.

Replicates

The RT-qPCR analysis was performed using three biological replicates.

Plotting

Values are shown as relative expression with WT set to 1 for each gene. Bars indicate mean relative expression, error bars indicate SEM, and individual data points represent biological replicates.

Figure3A_qPCR_TukeyHSD_all_comparisons.csv

This file contains the statistical results for all pairwise comparisons among genotypes for each gene shown in Figure 3A.

Statistical analysis

Statistical significance was assessed separately for each gene by one-way ANOVA followed by Tukey's HSD multiple-comparison test.

Significance labels

- *: P < 0.05

- **: P < 0.01

- ***: P < 0.001

- ****: P < 0.0001

- n.s.: not significant

Exact adjusted P values for significant comparisons

Dux:

- WT versus Ctbp1/2 DKO: adjusted P = 0.03777 (*)

- Dux KO versus Ctbp1/2 DKO: adjusted P = 0.006884 (**)

- Ctbp1/2 DKO versus Ctbp1/2/Dux TKO: adjusted P = 0.006782 (**)

Zscan4c:

- WT versus Ctbp1/2 DKO: adjusted P = 0.005695 (**)

- Dux KO versus Ctbp1/2 DKO: adjusted P = 0.002261 (**)

- Ctbp1/2 DKO versus Ctbp1/2/Dux TKO: adjusted P = 0.002271 (**)

Pramel7:

- WT versus Ctbp1/2/Dux TKO: adjusted P = 0.002418 (**)

- Dux KO versus Ctbp1/2/Dux TKO: adjusted P = 0.002241 (**)

Complete adjusted P values

Dux:

- WT versus Dux KO: adjusted P = 0.6008 (n.s.)

- WT versus Ctbp1/2 DKO: adjusted P = 0.03777 (*)

- WT versus Ctbp1/2/Dux TKO: adjusted P = 0.5941 (n.s.)

- Dux KO versus Ctbp1/2 DKO: adjusted P = 0.006884 (**)

- Dux KO versus Ctbp1/2/Dux TKO: adjusted P = 1 (n.s.)

- Ctbp1/2 DKO versus Ctbp1/2/Dux TKO: adjusted P = 0.006782 (**)

Zscan4c:

- WT versus Dux KO: adjusted P = 0.8635 (n.s.)

- WT versus Ctbp1/2 DKO: adjusted P = 0.005695 (**)

- WT versus Ctbp1/2/Dux TKO: adjusted P = 0.8652 (n.s.)

- Dux KO versus Ctbp1/2 DKO: adjusted P = 0.002261 (**)

- Dux KO versus Ctbp1/2/Dux TKO: adjusted P = 1 (n.s.)

- Ctbp1/2 DKO versus Ctbp1/2/Dux TKO: adjusted P = 0.002271 (**)

Pramel7:

- WT versus Dux KO: adjusted P = 0.9999 (n.s.)

- WT versus Ctbp1/2 DKO: adjusted P = 0.1441 (n.s.)

- WT versus Ctbp1/2/Dux TKO: adjusted P = 0.002418 (**)

- Dux KO versus Ctbp1/2 DKO: adjusted P = 0.1314 (n.s.)

- Dux KO versus Ctbp1/2/Dux TKO: adjusted P = 0.002241 (**)

- Ctbp1/2 DKO versus Ctbp1/2/Dux TKO: adjusted P = 0.05795 (n.s.)
