# Supplementary material for: CtBP1/2 restrict DUX-dependent and independent 2C-like programs in mouse embryonic stem cells
Source: EMBO Rep. 2026 Aug 3;27(18):5317–40. doi: 10.1038/s44319-026-00881-7 (PMC13601577; doi:10.1038/s44319-026-00881-7)
Supplement: Supplementary file 3 [file 44319_2026_881_MOESM10_ESM.zip › Figure_3_source_data/Panel_F/README.docx]

**README**

Source data for Figure 3F

File included

heatmap_data.xlsx

Description

This file contains the source data used to generate the K-means clustering heatmap shown in Figure 3F. Figure 3F shows K-means clustering of the top 2,000 most variable genes across WT, Dux KO, Ctbp1/2 DKO and Ctbp1/2/Dux TKO ESCs.

heatmap_data.xlsx

Each row represents one gene included in the heatmap.

Columns

- User_ID: Internal gene identifier used during data processing.

- ensembl_ID: Ensembl gene identifier.

- symbol: Gene symbol.

- WT_1, WT_2: Expression values for two biological replicates of WT ESCs.

- Dux_KO_1, Dux_KO_2: Expression values for two biological replicates of Dux KO ESCs.

- DKO_1, DKO_2: Expression values for two biological replicates of Ctbp1/2 DKO ESCs.

- DKO_Dux_KO_1, DKO_Dux_KO_2: Expression values for two biological replicates of Ctbp1/2/Dux TKO ESCs.

- cluster: K-means cluster assignment.

Data processing

The top 2,000 most variable genes were selected from the normalized RNA-seq expression matrix. Gene-wise standardized expression values were used for heatmap visualization. K-means clustering was performed with K = 5.

Cluster sizes

- Cluster 1: N = 184 genes

- Cluster 2: N = 632 genes

- Cluster 3: N = 334 genes

- Cluster 4: N = 577 genes

- Cluster 5: N = 273 genes

Replicates

The RNA-seq dataset includes two biological replicates per genotype.

Statistical analysis

No separate statistical test was performed for Figure 3F because this panel shows an unsupervised K-means clustering heatmap of normalized RNA-seq expression values. Differential-expression statistics for the underlying RNA-seq comparisons are provided in the corresponding RNA-seq source data files.

Notes

Representative genes are labeled in the figure for visualization. Complete gene-level expression values and cluster assignments are provided in heatmap_data.xlsx.
