# Supplementary material for: CtBP1/2 restrict DUX-dependent and independent 2C-like programs in mouse embryonic stem cells
Source: EMBO Rep. 2026 Aug 3;27(18):5317–40. doi: 10.1038/s44319-026-00881-7 (PMC13601577; doi:10.1038/s44319-026-00881-7)
Supplement: Supplementary file 3 [file 44319_2026_881_MOESM10_ESM.zip › Figure_3_source_data/Panel_H/README.docx]

**README**

Source data and statistical analysis for Figure 3H

Files included

1. iDux_RT-qPCR.csv

2. iDux_qPCR_TukeyHSD_by_gene.csv

Description

These files contain the source data and statistical analysis used to generate the RT-qPCR plot shown in Figure 3H. Figure 3H shows the expression of selected Cluster 1 and Cluster 3 genes after inducible Dux expression for 0 h and 24 h.

Genes analyzed

Zscan4c, Obox4, Pramel16, Gata1, Klf17, Pramel7

iDux_RT-qPCR.csv

This file contains the source RT-qPCR values plotted in Figure 3H.

Columns

- Gene: Gene analyzed by RT-qPCR.

- Time or condition: Dux induction condition, corresponding to 0 h or 24 h.

- Value: RT-qPCR value used for plotting and statistical analysis.

Replicates

The RT-qPCR analysis was performed using three biological replicates.

Plotting

ΔCt values were calculated as Ct(Gapdh) − Ct(target gene). Bars indicate mean ΔCt values, error bars indicate SEM, and individual data points represent biological replicates.

iDux_qPCR_TukeyHSD_by_gene.csv

This file contains the statistical results for comparisons between 0 h and 24 h for each gene.

Statistical analysis

Statistical significance was assessed separately for each gene by one-way ANOVA followed by Tukey's HSD multiple-comparison test.

Significance labels

- *: P < 0.05

- **: P < 0.01

- ***: P < 0.001

- ****: P < 0.0001

- n.s.: not significant

Exact adjusted P values

Zscan4c:

- 0 h versus 24 h: adjusted P = 3.16 × 10^-5 (****)

Obox4:

- 0 h versus 24 h: adjusted P = 8.18 × 10^-6 (****)

Pramel16:

- 0 h versus 24 h: adjusted P = 1.59 × 10^-5 (****)

Gata1:

- 0 h versus 24 h: adjusted P = 0.5101 (n.s.)

Klf17:

- 0 h versus 24 h: adjusted P = 0.03829 (*)

Pramel7:

- 0 h versus 24 h: adjusted P = 0.621 (n.s.)

Notes

Complete adjusted P values, including n.s. comparisons, are provided in iDux_qPCR_TukeyHSD_by_gene.csv.
