# Supplementary material for: CtBP1/2 restrict DUX-dependent and independent 2C-like programs in mouse embryonic stem cells
Source: EMBO Rep. 2026 Aug 3;27(18):5317–40. doi: 10.1038/s44319-026-00881-7 (PMC13601577; doi:10.1038/s44319-026-00881-7)
Supplement: Supplementary file 4 [file 44319_2026_881_MOESM11_ESM.zip › Figure_4_source_data/Panel_E_F/README.docx]

**README**

Source data for Figure 4E and Figure 4F

File included

response_gene_zscore.csv

Description

This file contains the source data used to generate the line plots shown in Figure 4E and Figure 4F. Figure 4E shows temporal expression dynamics of rapid-response genes, and Figure 4F shows temporal expression dynamics of delayed-response genes during the CtBP2 induction/depletion time course.

response_gene_zscore.csv

Each row corresponds to one gene at one time point.

Columns

- group: Response gene class. Rapid response genes were used for Figure 4E, and delayed response genes were used for Figure 4F.

- gene: Gene symbol.

- time: Sampling time point in the iCtBP2 time course.

- log2CPM_mean: Mean normalized expression value used for gene-wise Z-score calculation.

- zscore: Gene-wise Z-score used for plotting.

Gene sets and time points

- Delayed response genes: N = 44 genes; time points = d0, d12, d18, d3, d9

- Rapid response genes: N = 86 genes; time points = d0, d12, d18, d3, d9

Data processing

For each gene, Z-scores were calculated across the five time points using the mean normalized expression values. Individual gene trajectories and mean trajectories shown in Figure 4E and Figure 4F were calculated from the Z-score values in this file.

Replicates

The underlying RNA-seq dataset contains two biological replicates per time point. The plotted values are gene-wise Z-scores calculated from mean normalized expression values.
