# Supplementary material for: CtBP1/2 restrict DUX-dependent and independent 2C-like programs in mouse embryonic stem cells
Source: EMBO Rep. 2026 Aug 3;27(18):5317–40. doi: 10.1038/s44319-026-00881-7 (PMC13601577; doi:10.1038/s44319-026-00881-7)
Supplement: Supplementary file 4 [file 44319_2026_881_MOESM11_ESM.zip › Figure_4_source_data/Panel_B/README.docx]

**README**

**Source data for Figure 4B**

**File included**

**PCA_and_MDS.xlsx**

**Description**

**This file contains the source data used to generate the PCA plot shown in Figure 4B. Each row corresponds to one RNA-seq sample used in the PCA plot.**

**PCA_and_MDS.xlsx**

**Columns**

**- Sample: Sample name.**

**- PC1: Coordinate plotted on the x-axis.**

**- PC2: Coordinate plotted on the y-axis.**

**Samples included**

**The file contains two biological replicates for each time point: d0, d3, d9, d12 and d18.**

**Plotting**

**PC1 and PC2 values were plotted to visualize the relationship among samples during doxycycline-dependent CtBP2 induction and subsequent depletion.**
