# Supplementary material for: CtBP1/2 restrict DUX-dependent and independent 2C-like programs in mouse embryonic stem cells
Source: EMBO Rep. 2026 Aug 3;27(18):5317–40. doi: 10.1038/s44319-026-00881-7 (PMC13601577; doi:10.1038/s44319-026-00881-7)
Supplement: Supplementary file 4 [file 44319_2026_881_MOESM11_ESM.zip › Figure_4_source_data/Panel_G/README.docx]

**README**

Source data for Figure 4G

File included

heatmap_source_data_zscore.csv

Description

This file contains the source data used to generate the heatmap shown in Figure 4G. Figure 4G shows gene-wise Z-scores for selected rapid- and delayed-response genes across the iCtBP2 RNA-seq time course.

heatmap_source_data_zscore.csv

Each row corresponds to one gene. The iCt2 sample columns contain the gene-wise Z-score values used for heatmap visualization.

Columns

- Display_gene: Gene name displayed in the heatmap.

- Matched_symbol: Gene symbol matched in the RNA-seq expression matrix.

- Ensembl_gene_id: Ensembl gene identifier.

- User_ID_sorted_by_SD: Internal gene identifier used during data processing and ordering.

- iCt2_d0_1, iCt2_d0_2: Two biological replicates at day 0.

- iCt2_d3_1, iCt2_d3_2: Two biological replicates at day 3.

- iCt2_d9_1, iCt2_d9_2: Two biological replicates at day 9.

- iCt2_d12_1, iCt2_d12_2: Two biological replicates at day 12.

- iCt2_d18_1, iCt2_d18_2: Two biological replicates at day 18.

Genes included

- Klf17

- Pou2f3

- Pramel13

- Pramel7

- Pramef17

- Gata1

- Dux

- Zscan4c

- Zfp352

Data processing

Z-scores were calculated independently for each gene across the 10 iCtBP2 RNA-seq samples and were used directly for heatmap visualization.

Notes

The genes are shown in the same order as in the heatmap. Complete gene-wise Z-score values used for Figure 4G are provided in heatmap_source_data_zscore.csv.
