# Supplementary material for: CtBP1/2 restrict DUX-dependent and independent 2C-like programs in mouse embryonic stem cells
Source: EMBO Rep. 2026 Aug 3;27(18):5317–40. doi: 10.1038/s44319-026-00881-7 (PMC13601577; doi:10.1038/s44319-026-00881-7)
Supplement: Supplementary file 4 [file 44319_2026_881_MOESM11_ESM.zip › Figure_4_source_data/Panel_A/README.docx]

**README**

**Source data and statistical analysis for Figure 4A**

**Files included**

**1. DeltaCt.xlsx**

**2. DeltaCt_TukeyHSD_all_comparisons.csv**

**Description**

**These files contain the source data and statistical analysis used to generate the RT-qPCR time-course plots shown in Figure 4A. Figure 4A shows ΔCt values for Dux, MERVL, Zscan4c, Zfp352 and Pramel7 during doxycycline-dependent CtBP2 induction and subsequent CtBP2 depletion in Ctbp1/2 DKO ESCs.**

**DeltaCt.xlsx**

**This file contains the individual ΔCt values plotted in Figure 4A.**

**Columns**

**- Day: Sampling time point.**

**- Gene: Gene analyzed by RT-qPCR.**

**- DeltaCt: Measured ΔCt value for each biological replicate.**

**Replicates**

**The RT-qPCR analysis was performed using three biological replicates per time point.**

**Plotting**

**Dots represent individual biological replicate values. Connected lines represent the mean ΔCt value at each time point. Error bars indicate SEM.**

**DeltaCt_TukeyHSD_all_comparisons.csv**

**This file contains the statistical results for all pairwise comparisons among time points for each gene.**

**Statistical analysis**

**Statistical significance was assessed separately for each gene by one-way ANOVA followed by Tukey's HSD multiple-comparison test.**

**Significance labels**

**- *: P < 0.05**

**- **: P < 0.01**

**- ***: P < 0.001**

**- ****: P < 0.0001**

**- n.s.: not significant**

**Exact adjusted P values for statistically significant comparisons**

**Dux:**

**- d0 versus d3: adjusted P = 0.9879 (ns)**

**- d0 versus d9: adjusted P = 0.06215 (ns)**

**- d0 versus d12: adjusted P = 0.003758 (**)**

**- d0 versus d18: adjusted P = 0.6731 (ns)**

**- d9 versus d3: adjusted P = 0.1286 (ns)**

**- d12 versus d3: adjusted P = 0.007513 (**)**

**- d18 versus d3: adjusted P = 0.9026 (ns)**

**- d12 versus d9: adjusted P = 0.3965 (ns)**

**- d18 versus d9: adjusted P = 0.4202 (ns)**

**- d18 versus d12: adjusted P = 0.02805 (*)**

**Zscan4c:**

**- d0 versus d3: adjusted P = 0.937 (ns)**

**- d0 versus d9: adjusted P = 3.48 × 10^-4 (***)**

**- d0 versus d12: adjusted P = 2.19 × 10^-4 (***)**

**- d0 versus d18: adjusted P = 0.4698 (ns)**

**- d9 versus d3: adjusted P = 9.00 × 10^-4 (***)**

**- d12 versus d3: adjusted P = 5.48 × 10^-4 (***)**

**- d18 versus d3: adjusted P = 0.8701 (ns)**

**- d12 versus d9: adjusted P = 0.9944 (ns)**

**- d18 versus d9: adjusted P = 0.003276 (**)**

**- d18 versus d12: adjusted P = 0.001909 (**)**

**Zfp352:**

**- d0 versus d3: adjusted P = 0.1284 (ns)**

**- d0 versus d9: adjusted P = 5.87 × 10^-6 (***)**

**- d0 versus d12: adjusted P = 1.59 × 10^-5 (***)**

**- d0 versus d18: adjusted P = 0.07333 (ns)**

**- d9 versus d3: adjusted P = 7.52 × 10^-5 (***)**

**- d12 versus d3: adjusted P = 2.63 × 10^-4 (***)**

**- d18 versus d3: adjusted P = 0.9954 (ns)**

**- d12 versus d9: adjusted P = 0.7957 (ns)**

**- d18 versus d9: adjusted P = 1.12 × 10^-4 (***)**

**- d18 versus d12: adjusted P = 4.10 × 10^-4 (***)**

**Pramel7:**

**- d0 versus d3: adjusted P = 1.00 × 10^-6 (***)**

**- d0 versus d9: adjusted P = 7.82 × 10^-7 (***)**

**- d0 versus d12: adjusted P = 0.2486 (ns)**

**- d0 versus d18: adjusted P = 0.3736 (ns)**

**- d9 versus d3: adjusted P = 0.9964 (ns)**

**- d12 versus d3: adjusted P = 5.71 × 10^-6 (***)**

**- d18 versus d3: adjusted P = 2.80 × 10^-7 (***)**

**- d12 versus d9: adjusted P = 4.27 × 10^-6 (***)**

**- d18 versus d9: adjusted P = 2.27 × 10^-7 (***)**

**- d18 versus d12: adjusted P = 0.01388 (*)**

**Notes**

**Only selected comparisons are annotated in Figure 4A to avoid overloading the time-course plots. Complete Tukey HSD-adjusted P values, including n.s. comparisons, are provided in DeltaCt_TukeyHSD_all_comparisons.csv.**
