# Supplementary material for: CtBP1/2 restrict DUX-dependent and independent 2C-like programs in mouse embryonic stem cells
Source: EMBO Rep. 2026 Aug 3;27(18):5317–40. doi: 10.1038/s44319-026-00881-7 (PMC13601577; doi:10.1038/s44319-026-00881-7)
Supplement: Supplementary file 4 [file 44319_2026_881_MOESM11_ESM.zip › Figure_4_source_data/Panel_H/README.docx]

**README**

Source data and statistical analysis for Figure 4H

Files included

1. rapid_delayed_boxplot.xlsx

2. Rapid_Delayed_pairwise_Wilcoxon_BH.csv

Description

These files contain the source data and statistical analysis used to generate the box plot shown in Figure 4H. Figure 4H compares expression changes of rapid-response genes and delayed-response genes in two RNA-seq contrasts: Ctbp1/2 DKO versus WT and Ctbp1/2/Dux TKO versus Dux KO.

rapid_delayed_boxplot.xlsx

This file contains the gene-level values plotted in Figure 4H.

Columns

- Cell: Gene set and comparison group.

- Value: Expression change value used for plotting.

Groups included

- Rapid response, Ctbp1/2 DKO - WT: N = 90 genes

- Delayed response, Ctbp1/2 DKO - WT: N = 44 genes

- Rapid response, Ctbp1/2/Dux TKO - Dux KO: N = 90 genes

- Delayed response, Ctbp1/2/Dux TKO - Dux KO: N = 44 genes

Rapid_Delayed_pairwise_Wilcoxon_BH.csv

This file contains the statistical results for pairwise comparisons among the four plotted groups.

Statistical analysis

Statistical significance was assessed using two-sided Wilcoxon rank-sum tests followed by Benjamini-Hochberg correction for multiple comparisons.

Significance labels

- *: P < 0.05

- **: P < 0.01

- ***: P < 0.001

- ****: P < 0.0001

- n.s.: not significant

Exact adjusted P values

- Rapid response, Ctbp1/2 DKO - WT versus Delayed response, Ctbp1/2 DKO - WT: adjusted P = 1.35 × 10^-5 (***)

- Rapid response, Ctbp1/2 DKO - WT versus Rapid response, Ctbp1/2/Dux TKO - Dux KO: adjusted P = 5.27 × 10^-4 (***)

- Rapid response, Ctbp1/2 DKO - WT versus Delayed response, Ctbp1/2/Dux TKO - Dux KO: adjusted P = 0.003 (**)

- Delayed response, Ctbp1/2 DKO - WT versus Rapid response, Ctbp1/2/Dux TKO - Dux KO: adjusted P = 0.047 (*)

- Delayed response, Ctbp1/2 DKO - WT versus Delayed response, Ctbp1/2/Dux TKO - Dux KO: adjusted P = 1.35 × 10^-5 (***)

- Rapid response, Ctbp1/2/Dux TKO - Dux KO versus Delayed response, Ctbp1/2/Dux TKO - Dux KO: adjusted P = 6.30 × 10^-6 (***)

Notes

Box plots were generated from gene-level expression-change values. Complete nominal P values and Benjamini-Hochberg-adjusted P values are provided in Rapid_Delayed_pairwise_Wilcoxon_BH.csv.
