# Supplementary material for: CtBP1/2 restrict DUX-dependent and independent 2C-like programs in mouse embryonic stem cells
Source: EMBO Rep. 2026 Aug 3;27(18):5317–40. doi: 10.1038/s44319-026-00881-7 (PMC13601577; doi:10.1038/s44319-026-00881-7)
Supplement: Supplementary file 5 [file 44319_2026_881_MOESM12_ESM.zip › Figure_5_source_data/Panel_E/README.docx]

**README**

Source data and statistical analysis for Figure 5E

Files included

1. Gata1_Luc.xlsx

2. Gata1_Luc_TukeyHSD_all_comparisons.csv

Description

These files contain the source data and statistical analysis used to generate the luciferase reporter assay plot shown in Figure 5E. Figure 5E shows relative luciferase activity from the Control reporter and the Gata1_DE reporter in WT and Ctbp1/2 DKO ESCs with either empty vector or CtBP2 expression vector.

Gata1_Luc.xlsx

This file contains the relative luciferase activity values used for plotting Figure 5E.

Columns

- Reporter: Reporter construct used in the assay, either Control or Gata1_DE.

- Vec: Expression vector condition, either empty vector or CtBP2.

- Cell: Cell type, either WT or Ctbp1/2 DKO.

- Luc: Relative luciferase activity.

Replicates

The luciferase reporter assay was performed using three biological replicates per condition.

Plotting

Bars indicate the mean relative luciferase activity. Error bars indicate SEM. Individual data points represent biological replicates.

Gata1_Luc_TukeyHSD_all_comparisons.csv

This file contains the statistical results for all pairwise comparisons among plotted groups.

Statistical analysis

Statistical significance was assessed by one-way ANOVA followed by Tukey's HSD multiple-comparison test.

Significance labels

- *: P < 0.05

- **: P < 0.01

- ***: P < 0.001

- ****: P < 0.0001

- n.s.: not significant

Exact adjusted P values for annotated comparisons

- Control / empty / WT versus Gata1_DE / empty / WT: adjusted P = 2.17 × 10^-4 (***)

- Gata1_DE / empty / WT versus Gata1_DE / empty / Ctbp1/2 DKO: adjusted P = 0.001072 (**)

- Gata1_DE / empty / WT versus Gata1_DE / CtBP2 / WT: adjusted P = 0.09474 (n.s.)

- Gata1_DE / empty / Ctbp1/2 DKO versus Gata1_DE / CtBP2 / Ctbp1/2 DKO: adjusted P = 2.51 × 10^-4 (***)

Notes

Only selected comparisons are annotated in Figure 5E.

Complete Tukey HSD-adjusted P values, including n.s. comparisons, are provided in Gata1_Luc_TukeyHSD_all_comparisons.csv.
