# Supplementary material for: CtBP1/2 restrict DUX-dependent and independent 2C-like programs in mouse embryonic stem cells
Source: EMBO Rep. 2026 Aug 3;27(18):5317–40. doi: 10.1038/s44319-026-00881-7 (PMC13601577; doi:10.1038/s44319-026-00881-7)
Supplement: Supplementary file 5 [file 44319_2026_881_MOESM12_ESM.zip › Figure_5_source_data/Panel_G/README.docx]

**README**

The file CtBP2_ChIP_Percent_Input.xlsx contains the source data for the CtBP2 ChIP-qPCR analysis shown in Panel G.

Panel G shows CtBP2 ChIP-qPCR signal at the Pramel7 and Gata1 loci with or without doxycycline treatment. Dox + indicates doxycycline-induced CtBP2 expression, whereas Dox - indicates the non-induced condition.

The CtBP2_ChIP_Percent_Input.xlsx file contains the raw percent input values for each replicate.

The columns are as follows:

Dox: doxycycline treatment condition, either minus or plus.

Gene: genomic locus analyzed by ChIP-qPCR, either Pramel7 or Gata1.

Percent_Input: CtBP2 ChIP-qPCR signal expressed as percent input.

For visualization in the figure, data were grouped by gene and doxycycline treatment condition.

Bars indicate the mean percent input value.

Error bars indicate SEM.

The plotted genes are shown in the following order:

Pramel7

Gata1

Within each gene, Dox minus and Dox plus values are shown side by side.

The y-axis shows input (%).
