# Supplementary material for: CtBP1/2 restrict DUX-dependent and independent 2C-like programs in mouse embryonic stem cells
Source: EMBO Rep. 2026 Aug 3;27(18):5317–40. doi: 10.1038/s44319-026-00881-7 (PMC13601577; doi:10.1038/s44319-026-00881-7)
Supplement: Supplementary file 5 [file 44319_2026_881_MOESM12_ESM.zip › Figure_5_source_data/Panel_A/README.docx]

**README**

Source data and statistical analysis for Figure 5A

Files included

1. CtBP2_binding_regions.xlsx

2. Figure5A_CtBP2_binding_counts.csv

3. Figure5A_Fisher_exact_test.csv

Analysis script

Figure5A_CtBP2_binding_plot_statistics.R

Description

These files contain the source data and statistical analysis used to generate the stacked bar plot shown in Figure 5A. Figure 5A compares the numbers of CtBP2-binding and non-binding genes among rapid-response genes and delayed-response genes.

CtBP2_binding_regions.xlsx

This file contains CtBP2 ChIP-seq peak-to-gene association information used to define CtBP2-binding genes. CtBP2-binding genes were defined as genes associated with CtBP2 ChIP-seq peaks using GREAT version 4.0.4 with the mouse mm10/GRCm38 genome assembly. The association rule was “Two nearest genes: 1,000,000 bp max extension, curated regulatory domains included”.

Figure5A_CtBP2_binding_counts.csv

This file contains the gene numbers used for plotting Figure 5A.

Gene numbers used for Figure 5A

- Rapid-response genes: total = 89, CtBP2-binding = 40, non-binding = 49.

- Delayed-response genes: total = 48, CtBP2-binding = 2, non-binding = 46.

Figure5A_Fisher_exact_test.csv

This file contains the statistical test result for Figure 5A.

Statistical analysis

The proportion of CtBP2-binding genes was compared between rapid-response genes and delayed-response genes using a two-sided Fisher's exact test based on a 2 × 2 contingency table.

Contingency table

- Rapid-response genes: CtBP2-binding = 40, non-binding = 49.

- Delayed-response genes: CtBP2-binding = 2, non-binding = 46.

Exact P value

Rapid-response genes versus delayed-response genes: two-sided Fisher's exact test, P = 1.668e-07.

Significance labels

- *: P < 0.05

- **: P < 0.01

- ***: P < 0.001

- ****: P < 0.0001

- n.s.: not significant

Plotting

The stacked bar plot shows gene numbers, not percentages. CtBP2-binding genes are shown in red, and non-binding genes are shown in blue.

Notes

Rapid- and delayed-response genes were treated as mutually exclusive gene categories for Figure 5A. Slfn9 was present in both the rapid- and delayed-response gene lists and was therefore excluded from the mutually exclusive counting used for Figure 5A.
