# Supplementary material for: CtBP1/2 restrict DUX-dependent and independent 2C-like programs in mouse embryonic stem cells
Source: EMBO Rep. 2026 Aug 3;27(18):5317–40. doi: 10.1038/s44319-026-00881-7 (PMC13601577; doi:10.1038/s44319-026-00881-7)
Supplement: Supplementary file 5 [file 44319_2026_881_MOESM12_ESM.zip › Figure_5_source_data/Panel_F/README.docx]

**README**

Source data and statistical analysis for Figure 5G

Files included

1. WT_Suz12KO_qPCR_source_data.csv

2. Figure5G_WT_Suz12KO_qPCR_ttest_nominal_results.csv

Description

These files contain the source data and statistical analysis used to generate the RT-qPCR plot shown in Figure 5G. Figure 5G shows ΔCt values for representative genes in WT and Suz12 KO ESCs.

WT_Suz12KO_qPCR_source_data.csv

This file contains the ΔCt values used for plotting Figure 5G.

Columns

- Cell: Cell type, either WT or Suz12 KO.

- Gene: Gene analyzed by RT-qPCR.

- Ct: ΔCt value for each biological replicate.

- Cell2: Duplicate cell-type column used for plotting.

- Gene2: Duplicate gene-name column used for plotting.

Genes included

- Zscan4c

- Obox4

- Pramel16

- Gata1

- Klf17

- Pramel7

Replicates

The RT-qPCR analysis was performed using three biological replicates per cell type for each gene.

Plotting

Bars indicate the mean ΔCt value. Error bars indicate SEM. Individual data points represent biological replicates. Within each gene, WT and Suz12 KO values are shown side by side.

Figure5G_WT_Suz12KO_qPCR_ttest_nominal_results.csv

This file contains the statistical results for comparisons between WT and Suz12 KO for each gene.

Statistical analysis

Statistical significance was assessed for each gene by two-sided Welch's t-test comparing WT and Suz12 KO. Because each gene was tested as a single prespecified WT-versus-Suz12 KO comparison, no multiple-comparison correction was applied.

Significance labels

- *: P < 0.05

- **: P < 0.01

- ***: P < 0.001

- ****: P < 0.0001

- n.s.: not significant

Exact P values

- Zscan4c: P = 0.5704 (n.s.)

- Obox4: P = 0.4896 (n.s.)

- Pramel16: P = 0.9917 (n.s.)

- Gata1: P = 0.06206 (n.s.)

- Klf17: P = 0.6145 (n.s.)

- Pramel7: P = 0.01867 (*)

Notes

Complete t-test statistics, including t statistic, degrees of freedom and exact nominal P values, are provided in Figure5G_WT_Suz12KO_qPCR_ttest_nominal_results.csv.
