# Supplementary material for: CtBP1/2 restrict DUX-dependent and independent 2C-like programs in mouse embryonic stem cells
Source: EMBO Rep. 2026 Aug 3;27(18):5317–40. doi: 10.1038/s44319-026-00881-7 (PMC13601577; doi:10.1038/s44319-026-00881-7)
Supplement: Supplementary file 5 [file 44319_2026_881_MOESM12_ESM.zip › Figure_5_source_data/Panel_H/README.docx]

**README**

Source data for Figure 5H

Files included

- WT_DAPI.tif

- WT_GFP.tif

- WT_GATA1.tif

- DuxKO_DAPI.tif

- DuxKO_GFP.tif

- DuxKO_GATA1.tif

- DKO_DAPI.tif

- DKO_GFP.tif

- DKO_GATA1.tif

- TKO_DAPI.tif

- TKO_GFP.tif

- TKO_GATA1.tif

Description

These TIFF files contain the source image data used to generate the representative immunofluorescence images shown in Figure 5H. Figure 5H shows MERVL-EGFP and GATA1 expression in WT, Dux KO, Ctbp1/2 DKO, and Ctbp1/2/Dux TKO ESCs.

File organization

The source images are provided as separate single-channel TIFF files for each genotype and fluorescence channel. File names indicate the genotype and channel.

Genotypes

- WT: WT ESCs.

- DuxKO: Dux KO ESCs.

- DKO: Ctbp1/2 DKO ESCs.

- TKO: Ctbp1/2/Dux TKO ESCs.

Channels

- DAPI: Nuclear counterstain.

- GFP: MERVL-EGFP reporter signal.

- GATA1: GATA1 immunofluorescence signal.

Visualization

For Figure 5H, the GFP channel is shown in green, the GATA1 channel is shown in red, and the DAPI channel is shown in blue. Merge panels were generated by overlaying the GFP, GATA1, and DAPI channels. White arrowheads indicate representative cells highlighted in the figure. The scale bar is shown in the merged image panel.

Image processing

Brightness and contrast were adjusted uniformly for visualization.

Notes

Complete single-channel TIFF images used to generate the representative panels are provided as source data.
