# Supplementary material for: CtBP1/2 restrict DUX-dependent and independent 2C-like programs in mouse embryonic stem cells
Source: EMBO Rep. 2026 Aug 3;27(18):5317–40. doi: 10.1038/s44319-026-00881-7 (PMC13601577; doi:10.1038/s44319-026-00881-7)
Supplement: Supplementary file 5 [file 44319_2026_881_MOESM12_ESM.zip › Figure_5_source_data/Panel_B_C/README.docx]

**README**

The files H3K27Ac.bw and CTBP2.mm10.bw contain the source data for Panels B and C.

They provide genome-wide ChIP-seq signal tracks for H3K27Ac, and CtBP2, respectively, visualized at representative rapid response gene loci and delayed response gene loci.

The full DUX ChIP-seq bigWig file (Dux.bw) was not included in the EMBO Source Data package because of its large file size. The corresponding genome browser track will be deposited in GEO as processed data.

Panel B shows genome browser snapshots of the rapid response gene loci Pramel7 and Gata1.

Panel C shows genome browser snapshots of the delayed response gene loci Duxf3 and Zscan4c.

The H3K27Ac.bw file contains the H3K27Ac ChIP-seq signal based on the published dataset GSM4205678.

The CTBP2.mm10.bw file contains the CtBP2 ChIP-seq signal based on the published dataset ERR532426.

All genome browser tracks were visualized using the mouse mm10 / GRCm38 genome assembly.

The displayed genomic regions are as follows:

Pramel7: chr2:87,478,099-87,493,278

Gata1: chrX:7,945,694-8,023,359

Duxf3: chr10:58,220,525-58,234,153

Zscan4c: chr7:10,989,124-11,010,783

For visualization in the figure, the tracks are shown in the order H3K27Ac and CtBP2, and gene annotation.

Red horizontal bars indicate the genomic regions selected for downstream validation, including ChIP-qPCR and/or luciferase reporter assays.
