# Supplementary material for: CtBP1/2 restrict DUX-dependent and independent 2C-like programs in mouse embryonic stem cells
Source: EMBO Rep. 2026 Aug 3;27(18):5317–40. doi: 10.1038/s44319-026-00881-7 (PMC13601577; doi:10.1038/s44319-026-00881-7)
Supplement: Supplementary file 6 [file 44319_2026_881_MOESM13_ESM.zip › Figure_6_source_data/Panel_J/README.docx]

**README**

**Source data and statistical analysis for Figure 6J**

**Files included**

**1. Relative_values.xlsx**

**2. Relative_values_ttest_results.csv**

**Description**

**These files contain the source data and statistical analysis used to generate the RT-qPCR plot shown in Figure 6J. Figure 6J shows relative expression of Duxf3 in WT and Pramel7-OE ESCs.**

**Relative_values.xlsx**

**This file contains the individual biological replicate values used for plotting Figure 6J.**

**Columns**

**- Cell: Sample group, either WT or Pramel7-OE.**

**- DeltaCt: ΔCt value for each biological replicate.**

**- Relative values: Relative expression value used for plotting.**

**Replicates**

**The RT-qPCR analysis was performed using three biological replicates per sample group.**

**Plotting**

**Bars indicate the mean relative expression value. Error bars indicate SEM. Individual data points represent biological replicates.**

**Relative_values_ttest_results.csv**

**This file contains the statistical result for the WT versus Pramel7-OE comparison.**

**Statistical analysis**

**Statistical significance was assessed by two-sided Welch's t-test comparing WT and Pramel7-OE.**

**Exact P value**

**WT versus Pramel7-OE: P = 0.03454 (*).**

**Significance labels**

**- *: P < 0.05**

**- **: P < 0.01**

**- ***: P < 0.001**

**- ****: P < 0.0001**

**- n.s.: not significant**

**Notes**

**Complete t-test statistics, including t statistic, degrees of freedom and exact P value, are provided in Relative_values_ttest_results.csv.**
