# Supplementary material for: CtBP1/2 restrict DUX-dependent and independent 2C-like programs in mouse embryonic stem cells
Source: EMBO Rep. 2026 Aug 3;27(18):5317–40. doi: 10.1038/s44319-026-00881-7 (PMC13601577; doi:10.1038/s44319-026-00881-7)
Supplement: Supplementary file 6 [file 44319_2026_881_MOESM13_ESM.zip › Figure_6_source_data/Panel_C/README.docx]

**README**

**Source data for Figure 6C**

**Files included**

**- FLAG.jpg**

**- TUBULIN.tif**

**Description**

**These files contain the source immunoblot images used to generate Figure 6C. Figure 6C shows PRAMEL7 protein expression detected by α-FLAG immunoblotting and α-TUBULIN as the corresponding loading control.**

**File descriptions**

**- FLAG.jpg: α-FLAG immunoblot image showing FLAG-tagged PRAMEL7.**

**- TUBULIN.tif: α-TUBULIN immunoblot image used as the loading control.**

**Samples**

**The lanes correspond to the samples shown in Figure 6C: WT, Pramel7-OE, WT/Dux KO, and Pramel7-OE/Dux KO.**

**Image processing**

**The cropped blot images displayed in Figure 6C were prepared from these source images.**
