# Supplementary material for: CtBP1/2 restrict DUX-dependent and independent 2C-like programs in mouse embryonic stem cells
Source: EMBO Rep. 2026 Aug 3;27(18):5317–40. doi: 10.1038/s44319-026-00881-7 (PMC13601577; doi:10.1038/s44319-026-00881-7)
Supplement: Supplementary file 6 [file 44319_2026_881_MOESM13_ESM.zip › Figure_6_source_data/Panel_D/README.docx]

**README**

**Source data for Figure 6D**

**File included**

**Dux_Genotype.jpg**

**Description**

**This file contains the source gel image used to generate the PCR-based genotyping panel shown in Figure 6D. Figure 6D shows the Out-Out and In-Out PCR products used to confirm the targeted Dux locus.**

**Dux_Genotype.jpg**

**This file contains the original gel image. The boxed regions indicate the bands cropped and displayed in Figure 6D.**

**Samples**

**The lanes correspond to the samples shown in Figure 6D: WT, Pramel7-OE, WT/Dux KO, and Pramel7-OE/Dux KO.**

**Image processing**

**The gel images displayed in Figure 6D were prepared by cropping the relevant boxed regions from the source image.**
