# Supplementary material for: CtBP1/2 restrict DUX-dependent and independent 2C-like programs in mouse embryonic stem cells
Source: EMBO Rep. 2026 Aug 3;27(18):5317–40. doi: 10.1038/s44319-026-00881-7 (PMC13601577; doi:10.1038/s44319-026-00881-7)
Supplement: Supplementary file 6 [file 44319_2026_881_MOESM13_ESM.zip › Figure_6_source_data/Panel_E/README.docx]

**README**

**Source data for Figure 6E**

**Files included**

**- WT.jpg**

**- P7OE.jpg**

**- DuxKO.jpg**

**- P7OE_DuxKO.jpg**

**Description**

**These files contain the source fluorescence images used to generate the representative images shown in Figure 6E. Figure 6E shows MERVL-EGFP reporter signal in WT, Pramel7-OE, Dux KO, and Pramel7-OE/Dux KO ESCs.**

**File descriptions**

**- WT.jpg: MERVL-EGFP fluorescence image of WT ESCs.**

**- P7OE.jpg: MERVL-EGFP fluorescence image of Pramel7-OE ESCs.**

**- DuxKO.jpg: MERVL-EGFP fluorescence image of Dux KO ESCs.**

**- P7OE_DuxKO.jpg: MERVL-EGFP fluorescence image of Pramel7-OE/Dux KO ESCs.**

**Image processing**

**The images displayed in Figure 6E were prepared from these source images. Brightness and contrast were adjusted uniformly for visualization.**

**Notes**

**The scale bar shown in Figure 6E applies to all images.**
