# Supplementary material for: CtBP1/2 restrict DUX-dependent and independent 2C-like programs in mouse embryonic stem cells
Source: EMBO Rep. 2026 Aug 3;27(18):5317–40. doi: 10.1038/s44319-026-00881-7 (PMC13601577; doi:10.1038/s44319-026-00881-7)
Supplement: Supplementary file 6 [file 44319_2026_881_MOESM13_ESM.zip › Figure_6_source_data/Panel_B/README.docx]

**README**
The file Prame_preimplantarion_FPKM.xls contains the source data for **Panel B**.
It provides the **FPKM** expression values of Prame family genes during mouse preimplantation development and in embryonic stem cells, based on the published dataset **GSE71434**.
Each row corresponds to one gene, and each column corresponds to one developmental stage or cell type (**MII oocyte, PN5 zygote, early 2-cell, late 2-cell, 4-cell, 8-cell, ICM, and ESCs**).
The first column indicates the gene symbol.
For visualization in the figure, the heatmap colors represent **row-wise Z-scores** calculated from these FPKM values across all stages.
Genes are listed in the same order as shown in the heatmap.
