# Supplementary material for: CtBP1/2 restrict DUX-dependent and independent 2C-like programs in mouse embryonic stem cells
Source: EMBO Rep. 2026 Aug 3;27(18):5317–40. doi: 10.1038/s44319-026-00881-7 (PMC13601577; doi:10.1038/s44319-026-00881-7)
Supplement: Supplementary file 6 [file 44319_2026_881_MOESM13_ESM.zip › Figure_6_source_data/Panel_K/README.docx]

**README**

**Source data and statistical analysis for Figure 6K**

**Files included**

**1. DeltacCt_Relative_expression.xlsx**

**2. DuxKO_qPCR_ttest_results.csv**

**Description**

**These files contain the source data and statistical analysis used to generate the RT-qPCR plot shown in Figure 6K. Figure 6K shows Pramel7 expression in WT and Dux KO ESCs.**

**DeltacCt_Relative_expression.xlsx**

**This file contains the individual biological replicate values used for plotting Figure 6K.**

**Columns**

**- Cell: Biological replicate identity for WT or Dux KO ESCs.**

**- DeltaCt: ΔCt value for each biological replicate.**

**- Relative expression: Relative expression value used for plotting.**

**Replicates**

**The RT-qPCR analysis was performed using three biological replicates per genotype.**

**Plotting**

**Bars indicate the mean relative expression value. Error bars indicate SEM. Individual data points represent biological replicates.**

**DuxKO_qPCR_ttest_results.csv**

**This file contains the statistical result for the WT versus Dux KO comparison.**

**Statistical analysis**

**Statistical significance was assessed by two-sided Welch's t-test comparing WT and Dux KO.**

**Exact P value**

**WT versus Dux KO: P = 0.004388 (**).**

**Significance labels**

**- *: P < 0.05**

**- **: P < 0.01**

**- ***: P < 0.001**

**- ****: P < 0.0001**

**- n.s.: not significant**

**Notes**

**Complete t-test statistics, including t statistic, degrees of freedom and exact P value, are provided in DuxKO_qPCR_ttest_results.csv.**
