# Supplementary material for: CtBP1/2 restrict DUX-dependent and independent 2C-like programs in mouse embryonic stem cells
Source: EMBO Rep. 2026 Aug 3;27(18):5317–40. doi: 10.1038/s44319-026-00881-7 (PMC13601577; doi:10.1038/s44319-026-00881-7)
Supplement: Supplementary file 6 [file 44319_2026_881_MOESM13_ESM.zip › Figure_6_source_data/Panel_I/README.docx]

**README**

**Source data and enrichment analysis for Figure 6I**

**Files included**

**1. Enrichment.xlsx**

**2. Genesets.xls**

**3. Log2CPM.xlsx**

**Description**

**These files contain the source data used to generate the gene set enrichment dot plot shown in Figure 6I. Figure 6I shows enrichment of developmental and control gene sets across the K-means clusters shown in Figure 6H.**

**Enrichment.xlsx**

**This file contains the enrichment analysis results used to generate the dot plot. It includes the overlap, observed/expected ratio, P value, FDR, and -log10(FDR) for each gene set in each cluster.**

**Genesets.xls**

**This file contains the gene sets used for the enrichment analysis.**

**Log2CPM.xlsx**

**This file contains the expression matrix used for the upstream clustering analysis.**

**Plotting**

**In the dot plot, dot size represents the observed/expected ratio, and dot color represents -log10(FDR).**

**Notes**

**The K-means cluster assignments used for the enrichment analysis correspond to the clusters shown in Figure 6H.**
