# Supplementary material for: CtBP1/2 restrict DUX-dependent and independent 2C-like programs in mouse embryonic stem cells
Source: EMBO Rep. 2026 Aug 3;27(18):5317–40. doi: 10.1038/s44319-026-00881-7 (PMC13601577; doi:10.1038/s44319-026-00881-7)
Supplement: Supplementary file 6 [file 44319_2026_881_MOESM13_ESM.zip › Figure_6_source_data/Panel_A/README.docx]

**README**
The file Prame_Log2CPM.xlsx contains the source data for **Panel A.**
It provides the **log2CPM** expression values for Prame family genes across **WT, *Dux* KO, *Ctbp1/2* DKO, and *Ctbp1/2/Dux* TKO** samples, with two biological replicates for each genotype.
Each row corresponds to one gene, and each column corresponds to one sample.
Gene_symbol indicates the gene name.
The heatmap shown in Panel A was generated from these values after **row-wise Z-score transformation** across all samples.
Genes are displayed in the same order as in the plotted heatmap.
