# Supplementary material for: CtBP1/2 restrict DUX-dependent and independent 2C-like programs in mouse embryonic stem cells
Source: EMBO Rep. 2026 Aug 3;27(18):5317–40. doi: 10.1038/s44319-026-00881-7 (PMC13601577; doi:10.1038/s44319-026-00881-7)
Supplement: Supplementary file 6 [file 44319_2026_881_MOESM13_ESM.zip › Figure_6_source_data/Panel_F/README.docx]

**README**

**Source data for Figure 6F**

**File included**

**P7_FACS.pptx**

**Description**

**This PowerPoint file contains the source flow cytometry plots used to generate Figure 6F. Figure 6F shows representative flow cytometry analysis of MERVL-EGFP reporter activity in WT, Pramel7-OE, Dux KO, and Pramel7-OE/Dux KO ESCs.**

**Samples**

**- WT**

**- Pramel7-OE**

**- Dux KO**

**- Pramel7-OE/Dux KO**

**Flow cytometry plots**

**The plots show MERVL-EGFP reporter signal together with the corresponding flow cytometry channel used for gating. The percentages displayed in each plot indicate the gated MERVL-EGFP-positive population shown in the representative plot.**

**Image processing**

**The plots displayed in Figure 6F were prepared from the source images included in P7_FACS.pptx.**

**Notes**

**P7_FACS.pptx provides the representative plots used for Figure 6F.**
