# Supplementary material for: CtBP1/2 restrict DUX-dependent and independent 2C-like programs in mouse embryonic stem cells
Source: EMBO Rep. 2026 Aug 3;27(18):5317–40. doi: 10.1038/s44319-026-00881-7 (PMC13601577; doi:10.1038/s44319-026-00881-7)
Supplement: Supplementary file 6 [file 44319_2026_881_MOESM13_ESM.zip › Figure_6_source_data/Panel_H/README.docx]

**README**

**Source data for Figure 6H**

**File included**

**K-means_data.xlsx**

**Description**

**This file contains the source data used to generate the K-means clustering heatmap shown in Figure 6H. Figure 6H shows row-wise scaled expression values for 1,000 genes across WT, Dux KO, Pramel7-OE, and Pramel7-OE/Dux KO ESC samples.**

**K-means_data.xlsx**

**Each row corresponds to one gene.**

**Columns**

**- symbol: Gene symbol.**

**- WT_1, WT_2: Two biological replicates of WT ESCs.**

**- DuxKO_1, DuxKO_2: Two biological replicates of Dux KO ESCs.**

**- P7OE_1, P7OE_2: Two biological replicates of Pramel7-OE ESCs.**

**- P7OE_DuxKO_1, P7OE_DuxKO_2: Two biological replicates of Pramel7-OE/Dux KO ESCs.**

**- cluster: K-means cluster assignment for each gene.**

**Data processing**

**The values represent row-wise scaled expression values used for heatmap visualization. K-means clustering was performed with K = 5.**

**Notes**

**The file contains the 1,000 genes used for the heatmap.**
