# Supplementary material for: CtBP1/2 restrict DUX-dependent and independent 2C-like programs in mouse embryonic stem cells
Source: EMBO Rep. 2026 Aug 3;27(18):5317–40. doi: 10.1038/s44319-026-00881-7 (PMC13601577; doi:10.1038/s44319-026-00881-7)
Supplement: Supplementary file 7 [file 44319_2026_881_MOESM14_ESM.zip › Figure_7_source_data/Panel_D/README.docx]

**README**

Source data and statistical analysis for Figure 7D

Files included

1. table_control3000_with_highlight.tsv

2. table_overlap350_merged_with_highlight.tsv

3. overlap350_top20_absDelta.tsv

4. CpG_methylation_comparison_test_results.csv

Description

These files contain the source data and statistical analysis used to generate the CpG methylation comparison plot shown in Figure 7D. Figure 7D compares gene-level relative CpG methylation changes between randomly selected background genes and genes upregulated in both Pramel7-OE and Dnmt1 KO.

table_control3000_with_highlight.tsv

This file contains the gene-level values for randomly selected background genes (N = 3,000) shown on the left side of the plot.

table_overlap350_merged_with_highlight.tsv

This file contains the gene-level values for genes upregulated in both Pramel7-OE and Dnmt1 KO (N = 232) shown on the right side of the plot.

overlap350_top20_absDelta.tsv

This file contains the top 20 labeled genes highlighted in Figure 7D.

Columns in the gene-level source files

- symbol: Gene symbol.

- delta_mCG: Relative CpG methylation change, calculated as Pramel7-OE minus WT at TSS ±1 kb CpG island regions.

- mCG_WT: CpG methylation level in WT.

- mCG_P7OE: CpG methylation level in Pramel7-OE.

- n_regions: Number of assigned regions used for gene-level averaging.

- WT_mean: Mean expression value in WT.

- P7_mean: Mean expression value in Pramel7-OE.

- log2FC: Expression change in Pramel7-OE relative to WT.

- highlight: Label indicating whether the gene was highlighted in the figure.

Data processing

The plotted values represent gene-level ΔmCG (Pramel7-OE − WT) at TSS ±1 kb CpG island regions. Gene-level values were obtained by mapping RefSeq IDs to gene symbols and averaging values for regions assigned to the same gene.

CpG_methylation_comparison_test_results.csv

This file contains the statistical result for the comparison shown in Figure 7D.

Statistical analysis

Statistical significance was assessed by two-sided Wilcoxon rank-sum test comparing randomly selected background genes with genes upregulated in both Pramel7-OE and Dnmt1 KO.

Exact P value

Randomly selected genes versus genes upregulated in both Pramel7-OE and Dnmt1 KO: P = 4.73e-22.

Notes

The labeled genes in the plot correspond to those listed in overlap350_top20_absDelta.tsv.
