# Supplementary material for: CtBP1/2 restrict DUX-dependent and independent 2C-like programs in mouse embryonic stem cells
Source: EMBO Rep. 2026 Aug 3;27(18):5317–40. doi: 10.1038/s44319-026-00881-7 (PMC13601577; doi:10.1038/s44319-026-00881-7)
Supplement: Supplementary file 7 [file 44319_2026_881_MOESM14_ESM.zip › Figure_7_source_data/Panel_B/README.docx]

**README**
The files WT_sorted.bedGraph.gz and P7OE_sorted.bedGraph.gz contain the source data for **Panel B**.
These files provide the processed RRBS methylation track data used to generate the genome browser view shown for the ***Duxf3*** locus.
The displayed region corresponds to **chr10:58,229,742–58,233,060**.
WT_sorted.bedGraph.gz contains the track for **WT**, and P7OE_sorted.bedGraph.gz contains the track for **P7-OE**.
These processed track files were used to prepare the browser tracks shown in the figure.
Raw and processed RRBS sequencing data are deposited in **GEO** under the accession listed in the manuscript.
