# Supplementary material for: CtBP1/2 restrict DUX-dependent and independent 2C-like programs in mouse embryonic stem cells
Source: EMBO Rep. 2026 Aug 3;27(18):5317–40. doi: 10.1038/s44319-026-00881-7 (PMC13601577; doi:10.1038/s44319-026-00881-7)
Supplement: Supplementary file 7 [file 44319_2026_881_MOESM14_ESM.zip › Figure_7_source_data/Panel_I/README.docx]

**README**

**Source data and enrichment analysis for Figure 7I**

**Files included**

**1. Enrichment.xlsx**

**2. Geneset.xlsx**

**3. TKO up.xlsx**

**4. Dnmt1KO up.xls**

**5. Common.xlsx**

**Description**

**These files contain the source data used to generate the gene set enrichment dot plot shown in Figure 7I. Figure 7I shows enrichment of developmental and control gene sets among genes upregulated in Ctbp1/2/Dux TKO, Dnmt1 KO, or both.**

**Enrichment.xlsx**

**This file contains the enrichment analysis results used to generate the dot plot.**

**Columns**

**- query_set: Gene list tested for enrichment.**

**- ref_set: Reference gene set.**

**- overlap_gene_count: Number of overlapping genes between the query set and the reference gene set.**

**- overlap_gene_list: Overlapping genes.**

**- overlap: Observed overlap count.**

**- expected: Expected overlap count.**

**- observed_expected: Observed/expected ratio.**

**- odds_ratio: Odds ratio for enrichment.**

**- p_value: Nominal enrichment P value.**

**- FDR: FDR-adjusted P value.**

**- significant: Significance call based on FDR.**

**Geneset.xlsx**

**This file contains the reference gene sets used for the enrichment analysis.**

**TKO up.xlsx**

**This file contains the gene list used for the Ctbp1/2/Dux TKO group.**

**Dnmt1KO up.xls**

**This file contains the gene list used for the Dnmt1 KO group.**

**Common.xlsx**

**This file contains the gene list used for the Both group.**

**Plotting**

**In the dot plot, dot size represents the observed/expected ratio, and dot color represents -log10(FDR).**

**Notes**

**Complete overlap gene lists, nominal P values, FDR-adjusted P values and observed/expected ratios are provided in Enrichment.xlsx.**
