# Supplementary material for: CtBP1/2 restrict DUX-dependent and independent 2C-like programs in mouse embryonic stem cells
Source: EMBO Rep. 2026 Aug 3;27(18):5317–40. doi: 10.1038/s44319-026-00881-7 (PMC13601577; doi:10.1038/s44319-026-00881-7)
Supplement: Supplementary file 7 [file 44319_2026_881_MOESM14_ESM.zip › Figure_7_source_data/Panel_G/README.docx]

**README**

**Source data for Figure 7G**

**File included**

**Dux_locus_bisulfite_plot_and_stats.xlsx**

**Description**

**This file contains the source data used to generate the bisulfite sequencing methylation map shown in Figure 7G. Figure 7G shows CpG methylation at the Duxf3 locus in WT and Ctbp1/2 DKO ESCs.**

**Workbook sheets**

**- WT_matrix: Clone-by-CpG methylation matrix for WT ESCs.**

**- DKO_matrix: Clone-by-CpG methylation matrix for Ctbp1/2 DKO ESCs.**

**- Clone_summary: Clone-level summary of methylated calls, unmethylated calls, missing calls, non-missing calls and methylation percentage.**

**Methylation matrix**

**In the WT_matrix and DKO_matrix sheets, each row corresponds to one clone and each CpG column corresponds to one CpG site analyzed in the amplicon.**

**Methylation calls**

**- 1: methylated CpG call.**

**- 0: unmethylated CpG call.**

**- NA: missing or unassigned call.**

**Amplicon summary**

**- Number of CpG sites analyzed: 18.**

**- WT: 15 clones.**

**- Ctbp1/2 DKO: 13 clones.**

**Methylation percentages**

**The methylation percentages shown in Figure 7G were calculated from non-missing CpG calls.**

**- WT: 80.8% methylated CpG calls.**

**- Ctbp1/2 DKO: 66.4% methylated CpG calls.**

**Data processing**

**Methylation status was assigned based on the aligned bisulfite sequence relative to the genomic reference. CG calls were assigned as methylated, TG calls were assigned as unmethylated, and other calls were treated as missing values.**
