# Supplementary material for: CtBP1/2 restrict DUX-dependent and independent 2C-like programs in mouse embryonic stem cells
Source: EMBO Rep. 2026 Aug 3;27(18):5317–40. doi: 10.1038/s44319-026-00881-7 (PMC13601577; doi:10.1038/s44319-026-00881-7)
Supplement: Supplementary file 7 [file 44319_2026_881_MOESM14_ESM.zip › Figure_7_source_data/Panel_A/README.docx]

**README**

Source data for Figure 7A

Files included

- UHRF1.jpg

- TUBULIN.jpg

Description

These files contain the source immunoblot images used to generate Figure 7A. Figure 7A shows UHRF1 protein expression in WT and Pramel7-OE ESCs, with α-TUBULIN as the corresponding loading control.

File descriptions

- UHRF1.jpg: α-UHRF1 immunoblot image.

- TUBULIN.jpg: α-TUBULIN immunoblot image used as the loading control.

Samples

The lanes correspond to the samples shown in Figure 7A: WT and Pramel7-OE.

Image processing

The blot images displayed in Figure 7A were prepared by cropping the relevant boxed regions from these source images.
