# Supplementary material for: CtBP1/2 restrict DUX-dependent and independent 2C-like programs in mouse embryonic stem cells
Source: EMBO Rep. 2026 Aug 3;27(18):5317–40. doi: 10.1038/s44319-026-00881-7 (PMC13601577; doi:10.1038/s44319-026-00881-7)
Supplement: Supplementary file 8 [file 44319_2026_881_MOESM15_ESM.zip › Figure_8_source_data/Panel_D/README.docx]

**README**

**Source data and enrichment analysis for Figure 8D**

**File included**

**All_enrichment_stats.xlsx**

**Description**

**This file contains the source data used to generate the gene set enrichment dot plot shown in Figure 8D. Figure 8D shows enrichment of the triple-overlap genes against the indicated reference gene sets.**

**All_enrichment_stats.xlsx**

**Each row corresponds to one reference gene set tested in the enrichment analysis.**

**Columns**

**- query_set: Analyzed query gene set.**

**- ref_set: Reference gene set.**

**- overlap: Number of overlapping genes between the query set and the reference gene set.**

**- query_size: Number of genes in the query set used for enrichment analysis.**

**- ref_size: Number of genes in the reference gene set.**

**- universe_size: Number of genes in the background universe.**

**- expected: Expected overlap count.**

**- observed_expected: Observed/expected ratio used for dot size.**

**- odds_ratio: Odds ratio for enrichment.**

**- p_value: Nominal enrichment P value.**

**- FDR: FDR-adjusted P value used to calculate dot color.**

**- significant: Significance call based on FDR.**

**Plotting**

**In the dot plot, dot size represents the observed/expected ratio, and dot color represents -log10(FDR).**

**Notes**

**Complete overlap counts, nominal P values, FDR-adjusted P values, odds ratios, expected overlap counts and observed/expected ratios are provided in All_enrichment_stats.xlsx.**
