# Supplementary material for: CtBP1/2 restrict DUX-dependent and independent 2C-like programs in mouse embryonic stem cells
Source: EMBO Rep. 2026 Aug 3;27(18):5317–40. doi: 10.1038/s44319-026-00881-7 (PMC13601577; doi:10.1038/s44319-026-00881-7)
Supplement: Supplementary file 8 [file 44319_2026_881_MOESM15_ESM.zip › Figure_8_source_data/Panel_E/README.docx]

**README**

**Source data for Figure 8E**

**Files included**

**- WT_GFP.tif**

**- WT_UHRF1.tif**

**- WT_DAPI.tif**

**- WT_Merge.tif**

**- P7KO_GFP.tif**

**- P7KO_UHRF1.tif**

**- P7KO_DAPI.tif**

**- P7KO_Merge.tif**

**Description**

**These files contain the source fluorescence images used to generate the representative immunofluorescence images shown in Figure 8E. Figure 8E shows ZSCAN4 and UHRF1 immunostaining in WT and Pramel7 KO ESCs.**

**File organization**

**The source images are provided as single-channel and merged TIFF files for each genotype.**

**Genotypes**

**- WT: WT ESCs.**

**- P7KO: Pramel7 KO ESCs.**

**Channels**

**- GFP: ZSCAN4 immunostaining signal.**

**- UHRF1: UHRF1 immunostaining signal.**

**- DAPI: Nuclear counterstain.**

**- Merge: Merged image generated from the ZSCAN4, UHRF1, and DAPI channels.**

**Visualization**

**The images displayed in Figure 8E were prepared from these source images. White arrowheads indicate representative ZSCAN4-positive cells highlighted in the figure.**

**Image processing**

**Brightness and contrast were adjusted uniformly for visualization.**

**Notes**

**WT_Merge.tif and P7KO_Merge.tif contain the merged images generated from the corresponding single-channel images.**
