# Supplementary material for: CtBP1/2 restrict DUX-dependent and independent 2C-like programs in mouse embryonic stem cells
Source: EMBO Rep. 2026 Aug 3;27(18):5317–40. doi: 10.1038/s44319-026-00881-7 (PMC13601577; doi:10.1038/s44319-026-00881-7)
Supplement: Supplementary file 8 [file 44319_2026_881_MOESM15_ESM.zip › Figure_8_source_data/Panel_B/README.docx]

**README**

**Source data and GSEA results for Figure 8B**

**Files included**

**1. GSEA_fgsea_results_3sets.xlsx**

**2. GSEA_overlap_genes_in_rank_3sets.xlsx**

**Description**

**These files contain the source data and gene set enrichment analysis results used to generate the GSEA plots shown in Figure 8B. Figure 8B shows enrichment of three gene sets in the ranked gene list for the Dnmt1 KO versus WT comparison.**

**GSEA_fgsea_results_3sets.xlsx**

**This file contains the GSEA summary results for the three displayed gene sets.**

**Columns**

**- pathway: Gene set used for GSEA.**

**- pval: Nominal P value.**

**- padj: Adjusted P value.**

**- ES: Enrichment score.**

**- NES: Normalized enrichment score.**

**- nMoreExtreme: Number of more extreme permutations.**

**- size: Gene set size used in the analysis.**

**- leadingEdge_size: Number of leading-edge genes.**

**- leadingEdge_genes: Leading-edge genes.**

**GSEA_overlap_genes_in_rank_3sets.xlsx**

**This file contains the genes from each gene set that were present in the ranked gene list.**

**Columns**

**- pathway: Gene set name.**

**- symbol: Gene symbol.**

**- rank_position: Position of the gene in the ranked list.**

**- rank_stat: Ranking statistic used for GSEA.**

**- leading_edge: Whether the gene was included in the leading edge.**

**GSEA results**

**- Dux KO down: ES = 0.5549, NES = 1.924, P = 1.00e-04, adjusted P = 1.04e-04, gene set size = 803, leading-edge genes = 302.**

**- Dux KO & Pramel7 KO down: ES = 0.8319, NES = 2.723, P = 1.04e-04, adjusted P = 1.04e-04, gene set size = 138, leading-edge genes = 97.**

**- Pramel7 KO down: ES = 0.6675, NES = 2.301, P = 1.00e-04, adjusted P = 1.04e-04, gene set size = 539, leading-edge genes = 290.**

**Notes**

**The gene lists used for the Venn diagram in Figure 8A are provided separately in Dataset EV1.**
