# Supplementary material for: CtBP1/2 restrict DUX-dependent and independent 2C-like programs in mouse embryonic stem cells
Source: EMBO Rep. 2026 Aug 3;27(18):5317–40. doi: 10.1038/s44319-026-00881-7 (PMC13601577; doi:10.1038/s44319-026-00881-7)
Supplement: Supplementary file 8 [file 44319_2026_881_MOESM15_ESM.zip › Figure_8_source_data/Panel_G/README.docx]

**README**

Source data and statistical analysis for Figure 8G

Files included

1. DeltaCt.xlsx

2. DeltaCt_planned_comparisons.csv

Description

These files contain the source data and statistical analysis used to generate the RT-qPCR plots shown in Figure 8G. Figure 8G shows ΔCt values for Gpx2, Zfp980, and Pou3f1 in WT, Dux KO, Pramel7 KO, and Dux KO/Pramel7-OE ESCs treated with DMSO or GSK-3484862.

DeltaCt.xlsx

This file contains the source ΔCt values used for plotting and statistical analysis.

Columns

- Cell: Cell type or genotype used in the assay. Groups are WT, DuxKO, P7KO, and P7OE_DuxKO.

- Con: Treatment condition, either DMSO or GSK-3484862.

- Gene: Target gene, either Gpx2, Zfp980, or Pou3f1.

- DeltaCt: ΔCt value calculated as Ct(Gapdh) − Ct(Target).

Replicates

The RT-qPCR analysis was performed using three biological replicates per gene, cell type, and treatment condition.

Group counts

Gpx2:

- WT / DMSO: n = 3

- WT / GSK-3484862: n = 3

- DuxKO / DMSO: n = 3

- DuxKO / GSK-3484862: n = 3

- P7KO / DMSO: n = 3

- P7KO / GSK-3484862: n = 3

- P7OE_DuxKO / DMSO: n = 3

- P7OE_DuxKO / GSK-3484862: n = 3

Zfp980:

- WT / DMSO: n = 3

- WT / GSK-3484862: n = 3

- DuxKO / DMSO: n = 3

- DuxKO / GSK-3484862: n = 3

- P7KO / DMSO: n = 3

- P7KO / GSK-3484862: n = 3

- P7OE_DuxKO / DMSO: n = 3

- P7OE_DuxKO / GSK-3484862: n = 3

Pou3f1:

- WT / DMSO: n = 3

- WT / GSK-3484862: n = 3

- DuxKO / DMSO: n = 3

- DuxKO / GSK-3484862: n = 3

- P7KO / DMSO: n = 3

- P7KO / GSK-3484862: n = 3

- P7OE_DuxKO / DMSO: n = 3

- P7OE_DuxKO / GSK-3484862: n = 3

Plotting

For Figure 8G, data were grouped by gene, cell type, and treatment condition. Within each cell type, DMSO and GSK-3484862 conditions are shown side by side. Bars indicate the mean ΔCt value. Error bars indicate SEM. Individual data points represent biological replicates.

For visualization, bars were drawn upward from gene-specific baseline values to make negative ΔCt values easier to read. This baseline shift was used only for graphical display; statistical analyses were performed using the original ΔCt values in DeltaCt.xlsx.

Gene-specific plotting baselines

- Gpx2: baseline = −15.

- Zfp980: baseline = −9.

- Pou3f1: baseline = −13.

DeltaCt_planned_comparisons.csv

This file contains the planned-comparison results for Gpx2, Zfp980, and Pou3f1.

Statistical analysis

Statistical analysis was performed separately for each gene using a two-way ANOVA model: DeltaCt ~ Cell × Treatment. Planned comparisons were then performed using estimated marginal means. P values were adjusted using Holm correction within each gene. The main P value column used for figure annotation is p_adj_holm_within_gene.

Planned comparisons

- Cell-type comparisons under the DMSO condition.

- Cell-type comparisons under the GSK-3484862 condition.

- DMSO versus GSK-3484862 comparisons within each cell type.

Columns in DeltaCt_planned_comparisons.csv

- Gene: Target gene.

- comparison_type: Type of planned comparison.

- comparison_label: Readable comparison label.

- group1 and group2: Compared groups.

- estimate_group2_minus_group1: Estimated difference between group2 and group1.

- SE: Standard error.

- df: Degrees of freedom.

- t.ratio: t statistic from the planned comparison.

- raw_p: Unadjusted P value.

- p_adj_holm_within_gene: Holm-adjusted P value within each gene.

- p_adj_bonferroni_within_gene: Bonferroni-adjusted P value within each gene.

- significance: Significance label based on p_adj_holm_within_gene.

- test: Statistical test used.

- p_adj_holm_global: Holm-adjusted P value across all planned comparisons for all genes.

- p_adj_bonferroni_global: Bonferroni-adjusted P value across all planned comparisons for all genes.

- p_adj_holm_global_significance: Significance label based on p_adj_holm_global.

Significance labels

- *: P < 0.05

- **: P < 0.01

- ***: P < 0.001

- ****: P < 0.0001

- n.s.: not significant

Significant planned comparisons based on p_adj_holm_within_gene

Gpx2:

- WT DMSO vs Dux KO DMSO: Holm-adjusted P = 0.004036 (**)

- Dux KO DMSO vs Pramel7 KO DMSO: Holm-adjusted P = 1.03e-04 (***)

- Dux KO DMSO vs Dux KO/Pramel7-OE DMSO: Holm-adjusted P = 1.01e-04 (***)

- WT GSK-3484862 vs Dux KO GSK-3484862: Holm-adjusted P = 0.001408 (**)

- Dux KO GSK-3484862 vs Pramel7 KO GSK-3484862: Holm-adjusted P = 0.04007 (*)

- Dux KO GSK-3484862 vs Dux KO/Pramel7-OE GSK-3484862: Holm-adjusted P = 0.02169 (*)

- WT DMSO vs GSK-3484862: Holm-adjusted P = 0.002662 (**)

- Dux KO DMSO vs GSK-3484862: Holm-adjusted P = 0.007684 (**)

Zfp980:

- WT DMSO vs Dux KO DMSO: Holm-adjusted P = 0.005969 (**)

- WT DMSO vs Pramel7 KO DMSO: Holm-adjusted P = 0.00223 (**)

- WT DMSO vs Dux KO/Pramel7-OE DMSO: Holm-adjusted P = 7.60e-06 (****)

- Dux KO DMSO vs Dux KO/Pramel7-OE DMSO: Holm-adjusted P = 3.25e-08 (****)

- Pramel7 KO DMSO vs Dux KO/Pramel7-OE DMSO: Holm-adjusted P = 1.71e-08 (****)

- WT GSK-3484862 vs Dux KO/Pramel7-OE GSK-3484862: Holm-adjusted P = 0.02838 (*)

- Dux KO GSK-3484862 vs Dux KO/Pramel7-OE GSK-3484862: Holm-adjusted P = 0.002782 (**)

- Pramel7 KO GSK-3484862 vs Dux KO/Pramel7-OE GSK-3484862: Holm-adjusted P = 0.001009 (**)

- WT DMSO vs GSK-3484862: Holm-adjusted P = 4.31e-06 (****)

- Dux KO DMSO vs GSK-3484862: Holm-adjusted P = 9.65e-08 (****)

- Pramel7 KO DMSO vs GSK-3484862: Holm-adjusted P = 9.65e-08 (****)

- Dux KO/Pramel7-OE DMSO vs GSK-3484862: Holm-adjusted P = 0.01449 (*)

Pou3f1:

- WT GSK-3484862 vs Pramel7 KO GSK-3484862: Holm-adjusted P = 0.01727 (*)

- Dux KO GSK-3484862 vs Pramel7 KO GSK-3484862: Holm-adjusted P = 0.04213 (*)

Notes

Complete planned-comparison results and exact P values, including non-significant comparisons, are provided in DeltaCt_planned_comparisons.csv.
