# Supplementary material for: CtBP1/2 restrict DUX-dependent and independent 2C-like programs in mouse embryonic stem cells
Source: EMBO Rep. 2026 Aug 3;27(18):5317–40. doi: 10.1038/s44319-026-00881-7 (PMC13601577; doi:10.1038/s44319-026-00881-7)
Supplement: Supplementary file 8 [file 44319_2026_881_MOESM15_ESM.zip › Figure_8_source_data/Panel_F/README.docx]

**README**

Source data and statistical analysis for Figure 8F

Files included

1. Raw.csv

2. Mediancsv.csv

3. UHRF1_ZSCAN4_norm_2panel_wilcox_WT_vs_KO.csv

Description

These files contain the source data and statistical analysis used to generate the relative UHRF1 intensity violin plot shown in Figure 8F. Figure 8F shows relative UHRF1 intensity in WT and Pramel7 KO cells separated into ZSCAN4-negative and ZSCAN4-positive populations.

Raw.csv

This file contains the raw single-cell/ROI measurements.

Columns

- Plot_order: Plotting order used for the four groups.

- Cell: Genotype, either WT or KO.

- ZSCAN4_status: ZSCAN4 classification, either ZSCAN4- or ZSCAN4+.

- Image_ID: Source image identifier.

- ROI: ROI/cell identifier within each image.

- ZSCAN4_mean_intensity: Raw ZSCAN4 mean intensity.

- UHRF1_mean_intensity: Raw UHRF1 mean intensity.

- ZSCAN4_threshold: ZSCAN4 mean intensity threshold used for classification.

Mediancsv.csv

This file contains the normalized single-cell/ROI values used for plotting Figure 8F.

Additional columns in Mediancsv.csv

- group4: Combined plotting group.

- xpos: X-axis plotting position.

- ZSCAN4neg_n: Number of ZSCAN4-negative cells/ROIs used to calculate the genotype-specific baseline.

- ZSCAN4neg_median_UHRF1: Median UHRF1 intensity of ZSCAN4-negative cells within each genotype.

- ZSCAN4neg_mean_UHRF1: Mean UHRF1 intensity of ZSCAN4-negative cells within each genotype.

- UHRF1_norm_to_ZSCAN4neg_median: UHRF1_mean_intensity divided by the genotype-specific ZSCAN4-negative median. This value was used for the y-axis.

- UHRF1_percent_of_ZSCAN4neg_median: UHRF1_norm_to_ZSCAN4neg_median multiplied by 100.

Classification and normalization

Cells/ROIs were classified as ZSCAN4-negative when ZSCAN4_mean_intensity < 20 and ZSCAN4-positive when ZSCAN4_mean_intensity >= 20. UHRF1 intensity was normalized to the genotype-specific median UHRF1 intensity of ZSCAN4-negative cells.

Normalization formula

UHRF1_norm_to_ZSCAN4neg_median = UHRF1_mean_intensity / ZSCAN4neg_median_UHRF1

Group counts

- WT ZSCAN4-: n = 738

- KO ZSCAN4-: n = 2263

- WT ZSCAN4+: n = 36

- KO ZSCAN4+: n = 60

Baseline values used for normalization

- WT: ZSCAN4neg_n = 738, ZSCAN4neg_median_UHRF1 = 43.635, ZSCAN4neg_mean_UHRF1 = 45.71789024390244.

- KO: ZSCAN4neg_n = 2263, ZSCAN4neg_median_UHRF1 = 25.857, ZSCAN4neg_mean_UHRF1 = 28.182554131683606.

Plotting

Violin plots show the distribution of UHRF1_norm_to_ZSCAN4neg_median. Boxes indicate the median and interquartile range. Black dots indicate individual cells/ROIs. The number of cells/ROIs in each group is indicated above the corresponding violin plot.

UHRF1_ZSCAN4_norm_2panel_wilcox_WT_vs_KO.csv

This file contains the statistical results for WT versus KO comparisons within each ZSCAN4-status group.

Statistical analysis

WT and KO cells were compared separately within ZSCAN4-negative and ZSCAN4-positive populations using two-sided Wilcoxon rank-sum tests. Adjusted P values were calculated by multiple-testing correction across the two comparisons.

Exact P values

- ZSCAN4- WT versus KO: n = 738 and 2263, raw P = 0.770438, adjusted P = 1 (n.s.).

- ZSCAN4+ WT versus KO: n = 36 and 60, raw P = 6.35e-06, adjusted P = 1.27e-05 (****).

Significance labels

- *: P < 0.05

- **: P < 0.01

- ***: P < 0.001

- ****: P < 0.0001

- n.s.: not significant

Notes

Raw.csv is included for transparency of the original measurements. Mediancsv.csv contains the normalized values used for the plotted relative UHRF1 intensity. Complete statistical results are provided in UHRF1_ZSCAN4_norm_2panel_wilcox_WT_vs_KO.csv.
