# Supplementary material for: CtBP1/2 restrict DUX-dependent and independent 2C-like programs in mouse embryonic stem cells
Source: EMBO Rep. 2026 Aug 3;27(18):5317–40. doi: 10.1038/s44319-026-00881-7 (PMC13601577; doi:10.1038/s44319-026-00881-7)
Supplement: Supplementary file 9 [file 44319_2026_881_MOESM16_ESM.zip › Appendix_Figure_S1_source_data/Panel_E/README.docx]

**README**

Source data for Appendix Figure S1E

Files included

- CtBP1.tif

- CtBP2.tif

- TUBULIN.tif

Description

These files contain the source immunoblot images used to generate Appendix Figure S1E. Appendix Figure S1E shows CTBP1 and CTBP2 protein levels in WT and Ctbp1/2 DKO ESCs, with α-TUBULIN as the corresponding loading control.

File descriptions

- CtBP1.tif: α-CTBP1 immunoblot image.

- CtBP2.tif: α-CTBP2 immunoblot image.

- TUBULIN.tif: α-TUBULIN immunoblot image used as the loading control.

Samples

The lanes correspond to the samples shown in Appendix Figure S1E: WT and Ctbp1/2 DKO.

Image processing

The blot images displayed in Appendix Figure S1E were prepared from these source images.
