# Supplementary material for: CtBP1/2 restrict DUX-dependent and independent 2C-like programs in mouse embryonic stem cells
Source: EMBO Rep. 2026 Aug 3;27(18):5317–40. doi: 10.1038/s44319-026-00881-7 (PMC13601577; doi:10.1038/s44319-026-00881-7)
Supplement: Supplementary file 10 [file 44319_2026_881_MOESM17_ESM.zip › Appendix_Figure_S2_source_data/G/README.docx]

**README**

Source data and statistical analysis for Appendix Figure S2G

Files included

1. CtBP2_binding_regions.xlsx

2. Diff_genes_heatmap_DKO-WT.csv

3. processed_data.xlsx

4. CtBP2_enrichment_statistics_summary.txt

Description

These files contain the source data and statistical summary used to generate the CtBP2-associated gene enrichment bar plot shown in Appendix Figure S2G. Appendix Figure S2G shows the percentage of CtBP2-associated genes among all detected RNA-seq background genes, genes upregulated in Ctbp1/2 DKO ESCs relative to WT ESCs, and genes downregulated in Ctbp1/2 DKO ESCs relative to WT ESCs.

CtBP2_binding_regions.xlsx

This file contains CtBP2 ChIP-seq peak-to-gene association information generated using GREAT version 4.0.4 with the mouse mm10/GRCm38 genome assembly. CtBP2-associated genes were defined using the “Two nearest genes” association rule.

Diff_genes_heatmap_DKO-WT.csv

This file contains the differential-expression table for the Ctbp1/2 DKO versus WT RNA-seq comparison. It was used to define genes upregulated or downregulated in Ctbp1/2 DKO ESCs relative to WT ESCs.

processed_data.xlsx

This file contains the processed gene-level data used to assign genes to the plotted groups and to determine whether each gene was associated with CtBP2-binding regions.

CtBP2_enrichment_statistics_summary.txt

This file contains the values used for plotting and the statistical summary for Appendix Figure S2G.

Gene numbers used for Appendix Figure S2G

- All detected RNA-seq background genes: CtBP2-associated genes = 4,589 / 20,380 genes (22.5%).

- Ctbp1/2 DKO-upregulated genes: CtBP2-associated genes = 451 / 1,481 genes (30.5%).

- Ctbp1/2 DKO-downregulated genes: CtBP2-associated genes = 237 / 820 genes (28.9%).

Statistical analysis

Statistical enrichment of CtBP2-associated genes was assessed for Ctbp1/2 DKO-upregulated and Ctbp1/2 DKO-downregulated genes relative to the RNA-seq background using one-sided Fisher’s exact tests followed by Benjamini-Hochberg correction for multiple testing.

Exact P values

- Ctbp1/2 DKO-upregulated genes: odds ratio = 1.56, raw P = 1.25e-13, Benjamini-Hochberg-adjusted P = 2.50e-13.

- Ctbp1/2 DKO-downregulated genes: odds ratio = 1.42, raw P = 8.21e-06, Benjamini-Hochberg-adjusted P = 8.21e-06.

Significance labels

- *: P < 0.05

- **: P < 0.01

- ***: P < 0.001

- ****: P < 0.0001

- n.s.: not significant

Notes

Complete values used for plotting and statistical interpretation are provided in CtBP2_enrichment_statistics_summary.txt.
