# Supplementary material for: CtBP1/2 restrict DUX-dependent and independent 2C-like programs in mouse embryonic stem cells
Source: EMBO Rep. 2026 Aug 3;27(18):5317–40. doi: 10.1038/s44319-026-00881-7 (PMC13601577; doi:10.1038/s44319-026-00881-7)
Supplement: Supplementary file 10 [file 44319_2026_881_MOESM17_ESM.zip › Appendix_Figure_S2_source_data/H/README.docx]

**README**

Source data and statistical analysis for Appendix Figure S2H

Files included

1. P53 target.xlsx

2. WT_Dux_DKO_TKO.xlsx

3. p53_geneSet_stats.tsv

Description

These files contain the source data and statistical analysis used to generate the p53 target gene set expression plot shown in Appendix Figure S2H. Appendix Figure S2H shows the distribution of log2 expression values of p53 target genes in WT, Dux KO, Ctbp1/2 DKO, and Ctbp1/2/Dux TKO ESCs.

P53 target.xlsx

This file contains the curated p53 target gene set used for the analysis. The p53 target gene set contains 343 genes.

WT_Dux_DKO_TKO.xlsx

This file contains the gene expression matrix used for the analysis.

Columns

- gene_symbol: Gene symbol.

- WT_1, WT_2: Expression values from two biological replicates of WT ESCs.

- Dux_KO_1, Dux_KO_2: Expression values from two biological replicates of Dux KO ESCs.

- DKO_1, DKO_2: Expression values from two biological replicates of Ctbp1/2 DKO ESCs.

- TKO_1, TKO_2: Expression values from two biological replicates of Ctbp1/2/Dux TKO ESCs.

Genes used for plotting and statistics

Of the 343 genes in the p53 target gene set, 275 genes were detected in the RNA-seq dataset and used for visualization and statistical analysis.

Plotting

For visualization, expression values of the detected p53 target genes were averaged across biological replicates for each genotype. Each dot indicates one detected p53 target gene. Violin plots show the distribution of log2 expression values. Boxes indicate the median and interquartile range.

p53_geneSet_stats.tsv

This file contains pairwise Wilcoxon rank-sum test results with Benjamini-Hochberg-adjusted P values for all genotype comparisons.

Statistical analysis

Pairwise comparisons among WT, Dux KO, Ctbp1/2 DKO, and Ctbp1/2/Dux TKO were performed using two-sided Wilcoxon rank-sum tests followed by Benjamini-Hochberg correction for multiple testing.

Exact adjusted P values

- WT versus Dux KO: adjusted P = 0.75.

- WT versus Ctbp1/2 DKO: adjusted P = 0.588.

- WT versus Ctbp1/2/Dux TKO: adjusted P = 0.588.

- Dux KO versus Ctbp1/2 DKO: adjusted P = 0.675.

- Dux KO versus Ctbp1/2/Dux TKO: adjusted P = 0.588.

- Ctbp1/2 DKO versus Ctbp1/2/Dux TKO: adjusted P = 0.75.

Notes

Complete statistical results are provided in p53_geneSet_stats.tsv.
