# Supplementary material for: CtBP1/2 restrict DUX-dependent and independent 2C-like programs in mouse embryonic stem cells
Source: EMBO Rep. 2026 Aug 3;27(18):5317–40. doi: 10.1038/s44319-026-00881-7 (PMC13601577; doi:10.1038/s44319-026-00881-7)
Supplement: Supplementary file 10 [file 44319_2026_881_MOESM17_ESM.zip › Appendix_Figure_S2_source_data/M/README.docx]

**README**

The file WT_vs_DKO_TKO_selected_genes_dotplot_values.csv contains the source data for the selected gene dot plot shown in Panel M.

Panel M summarizes the expression of representative genes from selected gene-signature categories in Ctbp1/2 DKO and Ctbp1/2/Dux TKO ESCs relative to WT ESCs.

The plotted genes are grouped into the following gene-signature categories:
Minor ZGA
Major ZGA
TE
PrE
Naive
Formative
Committed
p53
Housekeeping

The WT_vs_DKO_TKO_selected_genes_dotplot_values.csv file contains the processed values used for plotting.
The columns are as follows:
symbol: gene symbol.
WT_mean: mean expression value in WT ESCs.
gene_group: gene-signature category.
genotype: mutant genotype shown in the plot, either DKO or TKO.
mutant_mean: mean expression value in the indicated mutant genotype.
wt_mean: mean expression value in WT ESCs, used as the reference value.
diff_vs_WT: difference between mutant_mean and wt_mean.

For visualization in the figure, dot size indicates mutant_mean.
Dot color indicates diff_vs_WT, calculated as mutant_mean − wt_mean.

Because the expression values are on a log scale, diff_vs_WT represents a log2FC-like difference relative to WT.

The genotypes are shown in the following order:
DKO
TKO

The selected genes are shown according to their gene-signature categories.
Each row represents one selected gene.
Each dot represents one genotype for the indicated gene.

No statistical hypothesis testing was performed for this panel.
This panel is intended to summarize representative expression changes in Ctbp1/2 DKO and Ctbp1/2/Dux TKO ESCs relative to WT ESCs.
