# Supplementary material for: CtBP1/2 restrict DUX-dependent and independent 2C-like programs in mouse embryonic stem cells
Source: EMBO Rep. 2026 Aug 3;27(18):5317–40. doi: 10.1038/s44319-026-00881-7 (PMC13601577; doi:10.1038/s44319-026-00881-7)
Supplement: Supplementary file 10 [file 44319_2026_881_MOESM17_ESM.zip › Appendix_Figure_S2_source_data/J/README.docx]

**README**

Source data and statistical analysis for Appendix Figure S2J

Files included

1. Obox3.csv

2. Obox4.csv

3. Obox6.csv

4. Obox8.csv

5. Obox_TukeyHSD_within_each_gene.csv

Description

These files contain the source data and statistical analysis used to generate the RT-qPCR plot shown in Appendix Figure S2J. Appendix Figure S2J shows relative expression levels of selected Obox genes in WT, Dux KO, Ctbp1/2 DKO, and Ctbp1/2/Dux TKO ESCs.

Source data files

- Obox3.csv: Obox3 expression data.

- Obox4.csv: Obox4 expression data.

- Obox6.csv: Obox6 expression data.

- Obox8.csv: Obox8 expression data.

Columns in source data files

- Cell: Cell type used in the assay. The plotted groups are WT, Dux KO, Ctbp1/2 DKO, and Ctbp1/2/Dux TKO.

- Relative: Relative expression level.

Replicates

The RT-qPCR analysis was performed using three biological replicates per cell type for each gene.

Group counts

Obox3:

- WT: n = 3

- Dux KO: n = 3

- Ctbp1/2 DKO: n = 3

- Ctbp1/2/Dux TKO: n = 3

Obox4:

- WT: n = 3

- Dux KO: n = 3

- Ctbp1/2 DKO: n = 3

- Ctbp1/2/Dux TKO: n = 3

Obox6:

- WT: n = 3

- Dux KO: n = 3

- Ctbp1/2 DKO: n = 3

- Ctbp1/2/Dux TKO: n = 3

Obox8:

- WT: n = 3

- Dux KO: n = 3

- Ctbp1/2 DKO: n = 3

- Ctbp1/2/Dux TKO: n = 3

Plotting

For visualization, data were grouped by gene and cell type. Bars indicate the mean relative expression level. Error bars indicate SEM. Individual data points represent biological replicates.

Obox_TukeyHSD_within_each_gene.csv

This file contains Tukey's HSD multiple-comparison test results for all pairwise comparisons among WT, Dux KO, Ctbp1/2 DKO, and Ctbp1/2/Dux TKO ESCs within each Obox gene.

Statistical analysis

Statistical significance was assessed separately for each gene by one-way ANOVA followed by Tukey's HSD multiple-comparison test. Adjusted P values were calculated using Tukey's HSD test.

Significance labels

- *: P < 0.05

- **: P < 0.01

- ***: P < 0.001

- ****: P < 0.0001

- n.s.: not significant

Exact adjusted P values

Obox3:

- Dux KO vs WT: adjusted P = 0.9998 (n.s.)

- Ctbp1/2 DKO vs WT: adjusted P = 0.2178 (n.s.)

- Ctbp1/2/Dux TKO vs WT: adjusted P = 0.9624 (n.s.)

- Ctbp1/2 DKO vs Dux KO: adjusted P = 0.1957 (n.s.)

- Ctbp1/2/Dux TKO vs Dux KO: adjusted P = 0.9425 (n.s.)

- Ctbp1/2/Dux TKO vs Ctbp1/2 DKO: adjusted P = 0.3962 (n.s.)

Obox4:

- Dux KO vs WT: adjusted P = 0.08589 (n.s.)

- Ctbp1/2 DKO vs WT: adjusted P = 1.16e-04 (***)

- Ctbp1/2/Dux TKO vs WT: adjusted P = 0.5118 (n.s.)

- Ctbp1/2 DKO vs Dux KO: adjusted P = 1.43e-05 (****)

- Ctbp1/2/Dux TKO vs Dux KO: adjusted P = 0.5439 (n.s.)

- Ctbp1/2/Dux TKO vs Ctbp1/2 DKO: adjusted P = 3.75e-05 (****)

Obox6:

- Dux KO vs WT: adjusted P = 0.9099 (n.s.)

- Ctbp1/2 DKO vs WT: adjusted P = 0.2187 (n.s.)

- Ctbp1/2/Dux TKO vs WT: adjusted P = 0.8967 (n.s.)

- Ctbp1/2 DKO vs Dux KO: adjusted P = 0.08804 (n.s.)

- Ctbp1/2/Dux TKO vs Dux KO: adjusted P = 1 (n.s.)

- Ctbp1/2/Dux TKO vs Ctbp1/2 DKO: adjusted P = 0.08371 (n.s.)

Obox8:

- Dux KO vs WT: adjusted P = 0.08235 (n.s.)

- Ctbp1/2 DKO vs WT: adjusted P = 0.9999 (n.s.)

- Ctbp1/2/Dux TKO vs WT: adjusted P = 0.9978 (n.s.)

- Ctbp1/2 DKO vs Dux KO: adjusted P = 0.0749 (n.s.)

- Ctbp1/2/Dux TKO vs Dux KO: adjusted P = 0.06392 (n.s.)

- Ctbp1/2/Dux TKO vs Ctbp1/2 DKO: adjusted P = 0.9995 (n.s.)

Notes

Complete adjusted P values for all within-gene pairwise comparisons are provided in Obox_TukeyHSD_within_each_gene.csv.
