# Supplementary material for: CtBP1/2 restrict DUX-dependent and independent 2C-like programs in mouse embryonic stem cells
Source: EMBO Rep. 2026 Aug 3;27(18):5317–40. doi: 10.1038/s44319-026-00881-7 (PMC13601577; doi:10.1038/s44319-026-00881-7)
Supplement: Supplementary file 10 [file 44319_2026_881_MOESM17_ESM.zip › Appendix_Figure_S2_source_data/D/README.docx]

**README**

Source data and statistical analysis for Appendix Figure S2D

Files included

1. Zscan4_Luc.xlsx

2. Zscan4_Luc_TukeyHSD_within_each_vector.csv

Description

These files contain the source data and statistical analysis used to generate the luciferase reporter assay plot shown in Appendix Figure S2D. Appendix Figure S2D shows relative luciferase activity from the Zscan4c luciferase reporter under Control, DUX, and Mt2_Mm vector conditions in WT, Dux KO, Ctbp1/2 DKO, and Ctbp1/2/Dux TKO ESCs.

Zscan4_Luc.xlsx

This file contains the relative luciferase activity values used for plotting Appendix Figure S2D.

Columns

- Vec: Vector condition used in the assay. The plotted conditions are Control, DUX, and Mt2_Mm. In the source file, the Mt2_Mm condition is listed as MERVL.

- Cell: Cell type used in the assay. The plotted groups are WT, Dux KO, Ctbp1/2 DKO, and Ctbp1/2/Dux TKO.

- Luc: Relative luciferase activity.

Replicates

The luciferase reporter assay was performed using three biological replicates per vector condition and cell type.

Group counts

- Control / WT: n = 3

- Control / Dux KO: n = 3

- Control / Ctbp1/2 DKO: n = 3

- Control / Ctbp1/2/Dux TKO: n = 3

- DUX / WT: n = 3

- DUX / Dux KO: n = 3

- DUX / Ctbp1/2 DKO: n = 3

- DUX / Ctbp1/2/Dux TKO: n = 3

- Mt2_Mm / WT: n = 3

- Mt2_Mm / Dux KO: n = 3

- Mt2_Mm / Ctbp1/2 DKO: n = 3

- Mt2_Mm / Ctbp1/2/Dux TKO: n = 3

Plotting

For visualization, data were grouped by vector condition and cell type. Bars indicate the mean relative luciferase activity. Error bars indicate SEM. Individual data points represent biological replicates.

Zscan4_Luc_TukeyHSD_within_each_vector.csv

This file contains Tukey's HSD multiple-comparison test results for all pairwise comparisons among WT, Dux KO, Ctbp1/2 DKO, and Ctbp1/2/Dux TKO ESCs within each vector condition.

Statistical analysis

Statistical significance was assessed within each vector condition by one-way ANOVA followed by Tukey's HSD multiple-comparison test. Adjusted P values were calculated using Tukey's HSD test.

Significance labels

- *: P < 0.05

- **: P < 0.01

- ***: P < 0.001

- ****: P < 0.0001

- n.s.: not significant

Exact adjusted P values

Control:

- Dux KO vs WT: adjusted P = 1 (n.s.)

- Ctbp1/2 DKO vs WT: adjusted P = 1 (n.s.)

- TKO vs WT: adjusted P = 1 (n.s.)

- Ctbp1/2 DKO vs Dux KO: adjusted P = 1 (n.s.)

- TKO vs Dux KO: adjusted P = 1 (n.s.)

- TKO vs Ctbp1/2 DKO: adjusted P = 1 (n.s.)

DUX:

- Dux KO vs WT: adjusted P = 0.5857 (n.s.)

- Ctbp1/2 DKO vs WT: adjusted P = 1.91e-06 (****)

- TKO vs WT: adjusted P = 0.7313 (n.s.)

- Ctbp1/2 DKO vs Dux KO: adjusted P = 9.94e-07 (****)

- TKO vs Dux KO: adjusted P = 0.9932 (n.s.)

- TKO vs Ctbp1/2 DKO: adjusted P = 1.13e-06 (****)

Mt2_Mm:

- Dux KO vs WT: adjusted P = 0.4798 (n.s.)

- Ctbp1/2 DKO vs WT: adjusted P = 5.34e-06 (****)

- TKO vs WT: adjusted P = 0.891 (n.s.)

- Ctbp1/2 DKO vs Dux KO: adjusted P = 2.35e-06 (****)

- TKO vs Dux KO: adjusted P = 0.2004 (n.s.)

- TKO vs Ctbp1/2 DKO: adjusted P = 8.14e-06 (****)

Notes

Complete adjusted P values for all within-vector pairwise comparisons are provided in Zscan4_Luc_TukeyHSD_within_each_vector.csv.
