# Supplementary material for: CtBP1/2 restrict DUX-dependent and independent 2C-like programs in mouse embryonic stem cells
Source: EMBO Rep. 2026 Aug 3;27(18):5317–40. doi: 10.1038/s44319-026-00881-7 (PMC13601577; doi:10.1038/s44319-026-00881-7)
Supplement: Supplementary file 10 [file 44319_2026_881_MOESM17_ESM.zip › Appendix_Figure_S2_source_data/E/README.docx]

**README**

Source data and statistical analysis for Appendix Figure S2E

Files included

1. Naalad2 Luc.xlsx

2. Naalad2_Luc_TukeyHSD_within_each_cell.csv

Description

These files contain the source data and statistical analysis used to generate the luciferase reporter assay plot shown in Appendix Figure S2E. Appendix Figure S2E shows relative luciferase activity from the Control reporter, MT2A reporter, and MT2C reporter in WT, Dux KO, Ctbp1/2 DKO, and Ctbp1/2/Dux TKO ESCs.

Naalad2 Luc.xlsx

This file contains the relative luciferase activity values used for plotting Appendix Figure S2E.

Columns

- Vec: Reporter construct used in the assay, either Control, MT2A, or MT2C.

- Cell: Cell type used in the assay. The plotted groups are WT, Dux KO, Ctbp1/2 DKO, and Ctbp1/2/Dux TKO.

- Luc: Relative luciferase activity.

Replicates

The luciferase reporter assay was performed using three biological replicates per reporter construct and cell type.

Group counts

- Control / WT: n = 3

- Control / Dux KO: n = 3

- Control / Ctbp1/2 DKO: n = 3

- Control / Ctbp1/2/Dux TKO: n = 3

- MT2A / WT: n = 3

- MT2A / Dux KO: n = 3

- MT2A / Ctbp1/2 DKO: n = 3

- MT2A / Ctbp1/2/Dux TKO: n = 3

- MT2C / WT: n = 3

- MT2C / Dux KO: n = 3

- MT2C / Ctbp1/2 DKO: n = 3

- MT2C / Ctbp1/2/Dux TKO: n = 3

Plotting

For visualization, data were grouped by reporter construct and cell type. Bars indicate the mean relative luciferase activity. Error bars indicate SEM. Individual data points represent biological replicates.

Naalad2_Luc_TukeyHSD_within_each_cell.csv

This file contains Tukey's HSD multiple-comparison test results for all pairwise comparisons among Control, MT2A, and MT2C within each cell type.

Statistical analysis

Statistical significance was assessed within each cell type by one-way ANOVA followed by Tukey's HSD multiple-comparison test. Adjusted P values were calculated using Tukey's HSD test.

Significance labels

- *: P < 0.05

- **: P < 0.01

- ***: P < 0.001

- ****: P < 0.0001

- n.s.: not significant

Exact adjusted P values

WT:

- MT2A vs Control: adjusted P = 0.031 (*)

- MT2C vs Control: adjusted P = 0.9357 (n.s.)

- MT2C vs MT2A: adjusted P = 0.02066 (*)

Dux KO:

- MT2A vs Control: adjusted P = 0.9266 (n.s.)

- MT2C vs Control: adjusted P = 0.01098 (*)

- MT2C vs MT2A: adjusted P = 0.007449 (**)

Ctbp1/2 DKO:

- MT2A vs Control: adjusted P = 3.45e-04 (***)

- MT2C vs Control: adjusted P = 0.9081 (n.s.)

- MT2C vs MT2A: adjusted P = 4.56e-04 (***)

Ctbp1/2/Dux TKO:

- MT2A vs Control: adjusted P = 0.009354 (**)

- MT2C vs Control: adjusted P = 0.9599 (n.s.)

- MT2C vs MT2A: adjusted P = 0.01249 (*)

Notes

Complete adjusted P values for all within-cell pairwise comparisons are provided in Naalad2_Luc_TukeyHSD_within_each_cell.csv.
