# Supplementary material for: CtBP1/2 restrict DUX-dependent and independent 2C-like programs in mouse embryonic stem cells
Source: EMBO Rep. 2026 Aug 3;27(18):5317–40. doi: 10.1038/s44319-026-00881-7 (PMC13601577; doi:10.1038/s44319-026-00881-7)
Supplement: Supplementary file 10 [file 44319_2026_881_MOESM17_ESM.zip › Appendix_Figure_S2_source_data/B/README.docx]

**READsME**

Source data and statistical analysis for Appendix Figure S2B

Files included

1. MERVL-Luc2.xlsx

2. MERVL_Luc_TukeyHSD_within_each_cell.csv

Description

These files contain the source data and statistical analysis used to generate the luciferase reporter assay plot shown in Appendix Figure S2B. Appendix Figure S2B shows relative luciferase activity from the Control reporter, MERVL-Luc reporter, and DUX-binding motif-mutated MERVL-Luc reporter in WT, Dux KO, Ctbp1/2 DKO, and Ctbp1/2/Dux TKO ESCs.

MERVL-Luc2.xlsx

This file contains the relative luciferase activity values used for plotting Appendix Figure S2B.

Columns

- Cell: Cell type used in the assay, either WT, Dux KO, Ctbp1/2 DKO, or Ctbp1/2/Dux TKO.

- Vector: Reporter construct used in the assay, either Control, MERVL-Luc, or DUXm MERVL-Luc.

- Luc: Relative luciferase activity.

Replicates

The luciferase reporter assay was performed using three biological replicates per cell type and reporter construct.

Group counts

- WT / Control: n = 3

- WT / MERVL-Luc: n = 3

- WT / DUXm MERVL-Luc: n = 3

- Dux KO / Control: n = 3

- Dux KO / MERVL-Luc: n = 3

- Dux KO / DUXm MERVL-Luc: n = 3

- DKO / Control: n = 3

- DKO / MERVL-Luc: n = 3

- DKO / DUXm MERVL-Luc: n = 3

- TKO / Control: n = 3

- TKO / MERVL-Luc: n = 3

- TKO / DUXm MERVL-Luc: n = 3

Plotting

For visualization, data were grouped by cell type and reporter construct. Bars indicate the mean relative luciferase activity. Error bars indicate SEM. Individual data points represent biological replicates.

MERVL_Luc_TukeyHSD_within_each_cell.csv

This file contains Tukey's HSD multiple-comparison test results for all pairwise comparisons among Control, MERVL-Luc, and DUXm MERVL-Luc within each cell type.

Statistical analysis

Statistical significance was assessed within each cell type by one-way ANOVA followed by Tukey's HSD multiple-comparison test. Adjusted P values were calculated using Tukey's HSD test.

Significance labels

- *: P < 0.05

- **: P < 0.01

- ***: P < 0.001

- ****: P < 0.0001

- n.s.: not significant

Exact adjusted P values

WT:

- MERVL-Luc vs Control: adjusted P = 1.06e-04 (***)

- DUXm MERVL-Luc vs Control: adjusted P = 0.02245 (*)

- DUXm MERVL-Luc vs MERVL-Luc: adjusted P = 1.79e-05 (****)

Dux KO:

- MERVL-Luc vs Control: adjusted P = 1.52e-04 (***)

- DUXm MERVL-Luc vs Control: adjusted P = 3.22e-04 (***)

- DUXm MERVL-Luc vs MERVL-Luc: adjusted P = 0.4805 (n.s.)

Ctbp1/2 DKO:

- MERVL-Luc vs Control: adjusted P = 1.69e-06 (****)

- DUXm MERVL-Luc vs Control: adjusted P = 0.4083 (n.s.)

- DUXm MERVL-Luc vs MERVL-Luc: adjusted P = 2.38e-06 (****)

Ctbp1/2/Dux TKO:

- MERVL-Luc vs Control: adjusted P = 0.01686 (*)

- DUXm MERVL-Luc vs Control: adjusted P = 0.01743 (*)

- DUXm MERVL-Luc vs MERVL-Luc: adjusted P = 0.9995 (n.s.)

Notes

Complete adjusted P values for all within-cell pairwise comparisons are provided in MERVL_Luc_TukeyHSD_within_each_cell.csv.
