# Supplementary material for: CtBP1/2 restrict DUX-dependent and independent 2C-like programs in mouse embryonic stem cells
Source: EMBO Rep. 2026 Aug 3;27(18):5317–40. doi: 10.1038/s44319-026-00881-7 (PMC13601577; doi:10.1038/s44319-026-00881-7)
Supplement: Supplementary file 10 [file 44319_2026_881_MOESM17_ESM.zip › Appendix_Figure_S2_source_data/K/README.docx]

**README**

Source data for Appendix Figure S2K

File included

WT_DKO_TKO_Clustering.csv

Description

This file contains the source data used to generate the K-means clustering plot shown in Appendix Figure S2K. Appendix Figure S2K shows cluster-wise expression patterns across WT, Dux KO, Ctbp1/2 DKO, and Ctbp1/2/Dux TKO ESCs.

WT_DKO_TKO_Clustering.csv

Each row corresponds to one gene.

Columns

- symbol: Gene symbol.

- WT_1, WT_2: Z-score values for two biological replicates of WT ESCs.

- Dux_KO_1, Dux_KO_2: Z-score values for two biological replicates of Dux KO ESCs.

- DKO_1, DKO_2: Z-score values for two biological replicates of Ctbp1/2 DKO ESCs.

- DKO_Dux_KO_1, DKO_Dux_KO_2: Z-score values for two biological replicates of Ctbp1/2/Dux TKO ESCs.

- cluster: K-means cluster assignment.

Data processing

K-means clustering was performed with K = 5 using 2,000 genes. The values in the sample columns are gene-wise Z-score-transformed expression values.

Cluster sizes

- Cluster 1: 184 genes.

- Cluster 2: 632 genes.

- Cluster 3: 334 genes.

- Cluster 4: 577 genes.

- Cluster 5: 273 genes.

Plotting

For Appendix Figure S2K, replicate values were averaged for each genotype when summarizing cluster-wise expression dynamics. Gray lines indicate individual genes within each cluster, and black lines indicate the mean Z-score for each cluster.

Notes

The file contains 2,000 genes and eight RNA-seq samples.
