# Supplementary material for: CtBP1/2 restrict DUX-dependent and independent 2C-like programs in mouse embryonic stem cells
Source: EMBO Rep. 2026 Aug 3;27(18):5317–40. doi: 10.1038/s44319-026-00881-7 (PMC13601577; doi:10.1038/s44319-026-00881-7)
Supplement: Supplementary file 10 [file 44319_2026_881_MOESM17_ESM.zip › Appendix_Figure_S2_source_data/L/README.docx]

**README**

Source data for Appendix Figure S2L

Files included

1. Cluster1.txt

2. Cluster2.txt

3. Cluster3.txt

4. Cluster4.txt

5. Preimplantation_Zhang_Nature_2016_high.txt

Description

These files contain the source data used to generate the heatmaps shown in Appendix Figure S2L. Appendix Figure S2L shows expression dynamics of genes in Clusters 1–4 during mouse preimplantation development using the published RNA-seq dataset from Zhang et al. (2016).

Cluster gene lists

- Cluster1.txt: Gene list for Cluster 1.

- Cluster2.txt: Gene list for Cluster 2.

- Cluster3.txt: Gene list for Cluster 3.

- Cluster4.txt: Gene list for Cluster 4.

Gene counts in the cluster-list files

- Cluster 1: 155 genes.

- Cluster 2: 611 genes.

- Cluster 3: 319 genes.

- Cluster 4: 572 genes.

Preimplantation_Zhang_Nature_2016_high.txt

This file contains the preimplantation-stage expression matrix used to generate the heatmaps.

Columns

- Gene: Gene symbol.

- MII oocyte: MII oocyte.

- PN5 zygote: PN5 zygote.

- Early 2-cell: Early 2-cell embryo.

- Late 2-cell: Late 2-cell embryo.

- 4-cell: 4-cell embryo.

- 8-cell: 8-cell embryo.

- ICM: Inner cell mass.

- mESC: Mouse embryonic stem cells.

Data processing

Genes with an average FPKM <= 1 were excluded from the analysis. Heatmaps were generated from the expression values after row-wise Z-score transformation across developmental stages and mESCs.

Notes

Cluster5.txt was not used for Appendix Figure S2L because the panel displays Clusters 1–4 only.
