# Supplementary material for: CtBP1/2 restrict DUX-dependent and independent 2C-like programs in mouse embryonic stem cells
Source: EMBO Rep. 2026 Aug 3;27(18):5317–40. doi: 10.1038/s44319-026-00881-7 (PMC13601577; doi:10.1038/s44319-026-00881-7)
Supplement: Supplementary file 11 [file 44319_2026_881_MOESM18_ESM.zip › Appendix_Figure_S3_source_data/A/README.docx]

**README**

Source data for Appendix Figure S3A

File included

PRAME_Sequence

Description

This file contains the amino acid sequences used to construct the molecular phylogenetic tree shown in Appendix Figure S3A. Appendix Figure S3A shows a maximum-likelihood phylogenetic tree of mouse PRAME family proteins, with LRRC14 used as an outgroup.

PRAME_Sequence

This file is a FASTA-format amino acid sequence file. Each entry contains one protein sequence used for phylogenetic tree construction.

Sequence summary

- Number of sequences: 76

- Minimum sequence length: 164 amino acids

- Maximum sequence length: 520 amino acids

Tree construction

The phylogenetic tree was constructed using MEGA. In MEGA, the tree was generated using the Construct/Test Maximum Likelihood Tree option. Amino acid sequences of mouse PRAME family proteins were used for tree construction, and LRRC14 was used as an outgroup.

Figure annotations

In Appendix Figure S3A, red labels indicate genes upregulated by Ctbp1/2 DKO in the absence of DUX, whereas blue labels indicate genes upregulated by Ctbp1/2 DKO in a DUX-dependent manner. Shaded regions indicate PRAME family gene clusters on the indicated chromosomes.

Notes

No statistical hypothesis testing was performed for Appendix Figure S3A. This panel is intended to show the phylogenetic relationship among PRAME family proteins and their chromosomal cluster annotations.
