# Supplementary material for: CtBP1/2 restrict DUX-dependent and independent 2C-like programs in mouse embryonic stem cells
Source: EMBO Rep. 2026 Aug 3;27(18):5317–40. doi: 10.1038/s44319-026-00881-7 (PMC13601577; doi:10.1038/s44319-026-00881-7)
Supplement: Supplementary file 11 [file 44319_2026_881_MOESM18_ESM.zip › Appendix_Figure_S3_source_data/C/README.docx]

**README**

Source data for Appendix Figure S3C

Files included

1. UHRF1.tif

2. TUBULIN.tif

Description

These files contain the source immunoblot images used to generate Appendix Figure S3C. Appendix Figure S3C shows UHRF1 protein levels in Pramel7 KO ESCs expressing PRAMEL7 (P7) or PRAMEL6 (P6), with α-TUBULIN as the loading control.

File descriptions

- UHRF1.tif: α-UHRF1 immunoblot image.

- TUBULIN.tif: α-TUBULIN immunoblot image used as the loading control.

Samples

The lanes correspond to the samples shown in Appendix Figure S3C:

- Pramel7 KO ESCs expressing PRAMEL7 (P7)

- Pramel7 KO ESCs expressing PRAMEL6 (P6)

Image processing

The blot images displayed in Appendix Figure S3C were prepared from these source images.
