# Supplementary material for: CtBP1/2 restrict DUX-dependent and independent 2C-like programs in mouse embryonic stem cells
Source: EMBO Rep. 2026 Aug 3;27(18):5317–40. doi: 10.1038/s44319-026-00881-7 (PMC13601577; doi:10.1038/s44319-026-00881-7)
Supplement: Supplementary file 16 [file 44319_2026_881_MOESM8_ESM.zip › Figure_1_source_data/Panel_C/README.docx]

README

This document describes the source data files used to generate the GSEA plot shown in Figure 1C. The source data package contains the ranked expression dataset, the gene set definition, and the exported GSEA results.

**Files included**

| **File name** | **Description** |
| --- | --- |
| WT_DKO_logCPM.gct | Expression dataset used as the GSEA input file. |
| WT_DKO_logCPM.csv | Spreadsheet version of the expression dataset. |
| geneset.csv | Gene set file used for the analysis of the 2CLC-enriched gene set. |
| GSEA.xlsx | Exported GSEA results used to prepare the plot shown in Figure 1C. |
