# Supplementary material for: CtBP1/2 restrict DUX-dependent and independent 2C-like programs in mouse embryonic stem cells
Source: EMBO Rep. 2026 Aug 3;27(18):5317–40. doi: 10.1038/s44319-026-00881-7 (PMC13601577; doi:10.1038/s44319-026-00881-7)
Supplement: Supplementary file 16 [file 44319_2026_881_MOESM8_ESM.zip › Figure_1_source_data/Panel_D/README.docx]

README

Source data for Figure 1D

Source data file

FACS_WT_DKO.pptx

Description

This source data file contains the flow cytometry plots and corresponding gate statistics used for Figure 1D. Figure 1D shows representative flow cytometry plots of MERVL-EGFP reporter activity in WT and Ctbp1/2 DKO ESCs.

The PowerPoint file contains one slide for each sample. Each slide shows the exported flow cytometry plot together with the corresponding gate statistics.

Samples included

WT-1

WT-2

WT-3

WT-4

WT-5

WT-6

DKO-1

DKO-2

DKO-3

DKO-4

DKO-5

DKO-6

Replicates

WT-1 to WT-6 and DKO-1 to DKO-6 represent biological replicate samples.

Gate statistics

Gate statistics indicate the percentage of MERVL-EGFP-positive cells in each sample. The same gate setting was applied consistently to WT and Ctbp1/2 DKO ESC samples.

Notes

No statistical test was performed for Figure 1D because this panel shows representative flow cytometry plots. The corresponding gate statistics for all samples are provided in the source data file.
