# Supplementary material for: CtBP1/2 restrict DUX-dependent and independent 2C-like programs in mouse embryonic stem cells
Source: EMBO Rep. 2026 Aug 3;27(18):5317–40. doi: 10.1038/s44319-026-00881-7 (PMC13601577; doi:10.1038/s44319-026-00881-7)
Supplement: Supplementary file 16 [file 44319_2026_881_MOESM8_ESM.zip › Figure_1_source_data/Panel_E/README.docx]

README for source image files

This folder contains representative fluorescence microscopy images used for the corresponding figure panel.

File descriptions:

- DAPI.tif: DAPI channel image showing nuclei.

- GFP.tif: GFP fluorescence channel image.

- MERVL.tif: MERVL reporter or MERVL-related fluorescence channel image.

- OCT4.tif: OCT4 immunofluorescence channel image.

- ZSCAN4.tif: ZSCAN4 immunofluorescence channel image.

Image format:

- All images are provided as TIFF files.

- Images correspond to the individual fluorescence channels used to generate the representative merged images shown in the figure.

- No quantitative processing was applied to these files for statistical analysis.

- Brightness and contrast may have been adjusted uniformly for visualization in the figure, without altering the interpretation of the data.
