# Supplementary material for: CtBP1/2 restrict DUX-dependent and independent 2C-like programs in mouse embryonic stem cells
Source: EMBO Rep. 2026 Aug 3;27(18):5317–40. doi: 10.1038/s44319-026-00881-7 (PMC13601577; doi:10.1038/s44319-026-00881-7)
Supplement: Supplementary file 16 [file 44319_2026_881_MOESM8_ESM.zip › Figure_1_source_data/Panel_B/README.docx]

**README**

Source data file

DESeq2_WT_DKO_repeat_results.xlsx

Description

This file contains the DESeq2 differential-expression results for repetitive elements used to generate the MA plot in Figure 1B. Each row represents one repetitive element or repeat-family annotation. The x-axis in Figure 1B shows log2(mean normalized counts), calculated from the DESeq2 baseMean value. The y-axis shows the log2 fold change for Ctbp1/2 DKO versus WT ESCs.

The RNA-seq analysis was performed using two biological replicates per genotype.

Columns

- baseMean: Mean of DESeq2-normalized counts across the analyzed WT and Ctbp1/2 DKO RNA-seq samples.

- log2FoldChange: DESeq2-estimated log2 fold change, calculated as Ctbp1/2 DKO relative to WT.

- pvalue: Nominal P value from the DESeq2 Wald test.

- padj: Benjamini-Hochberg-adjusted P value.

- gene_id: Repetitive element or repeat-family identifier used for plotting and labeling selected elements.

- log(baseMean): Auxiliary log-transformed baseMean value included in the source data file.

Statistical analysis

Differential expression of repetitive elements was assessed using DESeq2 with a Wald test. P values were adjusted for multiple testing using the Benjamini-Hochberg method.

Plotting and color coding

- Red dots: significantly upregulated repetitive elements in Ctbp1/2 DKO versus WT ESCs.

- Blue dots: significantly downregulated repetitive elements in Ctbp1/2 DKO versus WT ESCs.

- Gray dots: repetitive elements that did not meet the significance and fold-change thresholds used for the MA plot.

Notes

No separate P-value annotations are shown in Figure 1B because the panel is a transcriptome-wide MA plot for repetitive elements. Exact nominal P values and adjusted P values for all repetitive elements are provided in the source data file.
