# Supplementary material for: CtBP1/2 restrict DUX-dependent and independent 2C-like programs in mouse embryonic stem cells
Source: EMBO Rep. 2026 Aug 3;27(18):5317–40. doi: 10.1038/s44319-026-00881-7 (PMC13601577; doi:10.1038/s44319-026-00881-7)
Supplement: Supplementary file 16 [file 44319_2026_881_MOESM8_ESM.zip › Figure_1_source_data/Panel_A/README.docx]

**README**

Source data file

DESeq2_WT_DKO_result.xls

Description

This file contains the DESeq2 differential-expression results used to generate the MA plot in Figure 1A. Each row represents one gene. The x-axis in Figure 1A shows log2(mean normalized counts), derived from the DESeq2 baseMean value. The y-axis shows log2 fold change for Ctbp1/2 DKO versus WT ESCs.

Columns

- gene_id: Gene symbol or gene identifier used for plotting and labeling selected genes.

- baseMean: Mean of DESeq2-normalized counts across the analyzed WT and Ctbp1/2 DKO RNA-seq samples.

- log2FoldChange: DESeq2-estimated log2 fold change, calculated as Ctbp1/2 DKO relative to WT.

- lfcSE: Standard error of the log2 fold-change estimate.

- stat: Wald test statistic calculated by DESeq2.

- pvalue: Nominal P value from the DESeq2 Wald test.

- padj: Benjamini-Hochberg-adjusted P value.

Statistical analysis

Differential expression was assessed using DESeq2 with a Wald test. P values were adjusted for multiple testing using the Benjamini-Hochberg method. Genes were considered significantly differentially expressed when adjusted P <= 0.05 and absolute fold change >= 2, corresponding to |log2FoldChange| >= 1.

Plotting and color coding

- Red dots: significantly upregulated genes in Ctbp1/2 DKO versus WT ESCs (padj <= 0.05 and log2FoldChange >= 1).

- Blue dots: significantly downregulated genes in Ctbp1/2 DKO versus WT ESCs (padj <= 0.05 and log2FoldChange <= -1).

- Gray dots: genes that did not meet the significance and fold-change thresholds.

Summary shown in the figure

- Upregulated genes: 1,195

- Downregulated genes: 1,368

Notes

No separate P-value annotations are shown in Figure 1A because the panel is a transcriptome-wide MA plot. Exact nominal P values and adjusted P values for all genes are provided in the source data file.
