# Supplementary material for: CtBP1/2 restrict DUX-dependent and independent 2C-like programs in mouse embryonic stem cells
Source: EMBO Rep. 2026 Aug 3;27(18):5317–40. doi: 10.1038/s44319-026-00881-7 (PMC13601577; doi:10.1038/s44319-026-00881-7)
Supplement: Supplementary file 17 [file 44319_2026_881_MOESM9_ESM.zip › Figure_2_source_data/Panel_J/README.docx]

**README**

Source data and statistical analysis for Figure 2J

Files included

1. max_change.csv

2. Max_change_pvalues_Bonferroni.csv

Description

These files contain the numerical source data and statistical analysis used to generate the violin plot shown in Figure 2J. Figure 2J shows the maximal fluorescence change per hour for each tracked MERVL-EGFP-positive cell in WT, Ctbp1/2 DKO and Ctbp1/2/Dux TKO ESCs.

max_change.csv

This file contains the source values plotted in Figure 2J.

Columns

- Cell: Cell type or genotype analyzed.

- Change: Maximal fluorescence change per hour for each tracked cell.

Fluorescence intensity traces were processed using a 3-frame rolling median. The maximal fluorescence change per hour was calculated for each cell from the smoothed trace. Cells with an initial fluorescence intensity of 200 or greater were excluded from this analysis.

Tracked cells included

- WT: n = 71 tracked cells

- Ctbp1/2 DKO: n = 107 tracked cells

- Ctbp1/2/Dux TKO: n = 71 tracked cells

Max_change_pvalues_Bonferroni.csv

This file contains the statistical results for pairwise comparisons among genotypes.

Statistical analysis

Statistical significance was assessed using two-sided Wilcoxon rank-sum tests followed by Bonferroni correction for multiple comparisons.

Significance labels

- *: P < 0.05

- **: P < 0.01

- ***: P < 0.001

- ****: P < 0.0001

- n.s.: not significant

Exact adjusted P values

- WT versus Ctbp1/2 DKO: adjusted P = 3.92 × 10^-11 (****)

- WT versus Ctbp1/2/Dux TKO: adjusted P = 1.10 × 10^-11 (****)

- Ctbp1/2 DKO versus Ctbp1/2/Dux TKO: adjusted P = 0.0745 (n.s.)

Notes

Only selected comparisons are annotated in the figure. Complete nominal P values and Bonferroni-adjusted P values are provided in Max_change_pvalues_Bonferroni.csv.
