# Supplementary material for: CtBP1/2 restrict DUX-dependent and independent 2C-like programs in mouse embryonic stem cells
Source: EMBO Rep. 2026 Aug 3;27(18):5317–40. doi: 10.1038/s44319-026-00881-7 (PMC13601577; doi:10.1038/s44319-026-00881-7)
Supplement: Supplementary file 17 [file 44319_2026_881_MOESM9_ESM.zip › Figure_2_source_data/Panel_C/README.docx]

**README**

Source data and statistical analysis for Figure 2C

Source data file

cell growth.xlsx

Analysis script

Figure2C_growth_statistics.R

Output files generated by the analysis script

- Figure2C_summary_stats.csv

- Figure2C_pairwise_Welch_Bonferroni.csv

- Figure2C_growth_curve.pdf

- Figure2C_growth_curve.png

Description

These files contain the numerical source data and statistical analysis used for the growth curve shown in Figure 2C. Figure 2C shows relative cell numbers for wild-type (WT), Dux KO, Ctbp1/2 DKO and Ctbp1/2/Dux TKO ESCs over the indicated culture period.

Replicates

The analysis was performed using three biological replicates per genotype at each time point.

Input data columns

- Cell: Genotype or cell line analyzed.

- day: Culture day.

- Relative: Relative cell number.

Plotting

Individual data points represent biological replicates. Lines show the mean relative cell number for each genotype. Error bars indicate SEM. The y-axis is shown on a logarithmic scale.

Statistical analysis

Statistical testing was performed on log10-transformed Relative values because the growth curve spans several orders of magnitude and is displayed on a logarithmic y-axis.

For each non-baseline time point, pairwise comparisons among genotypes were performed using a two-sided Welch's t-test. P values were adjusted using Bonferroni correction. The output file provides both Bonferroni-adjusted P values within each day and Bonferroni-adjusted P values across all pairwise comparisons at days 2, 4, 6 and 8.

Day 0 was not tested because all samples were set to the baseline relative cell number of 1.

Significance labels

- *: P < 0.05

- **: P < 0.01

- ***: P < 0.001

- ****: P < 0.0001

- n.s.: not significant

Notes

Complete pairwise nominal P values and Bonferroni-adjusted P values are provided in Figure2C_pairwise_Welch_Bonferroni.csv. If only selected comparisons are annotated in the figure, the remaining comparisons are documented in the source data file.
