# Supplementary material for: CtBP1/2 restrict DUX-dependent and independent 2C-like programs in mouse embryonic stem cells
Source: EMBO Rep. 2026 Aug 3;27(18):5317–40. doi: 10.1038/s44319-026-00881-7 (PMC13601577; doi:10.1038/s44319-026-00881-7)
Supplement: Supplementary file 17 [file 44319_2026_881_MOESM9_ESM.zip › Figure_2_source_data/Panel_B/README.docx]

**README**

Source data for Figure 2B

Source data files

FACS_data.csv

WT_DuxKO_DKO_TKO_pairwise_ttest_Bonferroni.csv

Description

These files contain the numerical data and statistical analysis used to generate the bar plot shown in Figure 2B. Figure 2B shows the percentage of MERVL-EGFP-positive cells in wild-type (WT), Dux KO, Ctbp1/2 DKO and Ctbp1/2/Dux TKO ESCs.

Replicates

The analysis was performed using three biological replicates per genotype.

FACS_data.csv

This file contains the source values plotted in Figure 2B.

Columns

- Cell: Genotype or cell line analyzed.

- Col: Measured population. GFP indicates MERVL-EGFP-positive cells.

- Percent: Percentage of MERVL-EGFP-positive cells.

Plotting

Bars indicate the mean percentage of MERVL-EGFP-positive cells. Error bars indicate SEM. Individual data points are shown.

WT_DuxKO_DKO_TKO_pairwise_ttest_Bonferroni.csv

This file contains the pairwise statistical comparisons used for Figure 2B.

Columns

- Col: Measured population.

- group1, group2: Pairwise comparison groups.

- n1, n2: Number of biological replicates in each group.

- mean1, mean2: Mean percentage values for each group.

- mean_difference_group2_minus_group1: Difference in means, calculated as group2 minus group1.

- statistic: Welch's t statistic.

- df: Degrees of freedom.

- p_value: Nominal P value from the two-sided Welch's t-test.

- p_adj_bonferroni: Bonferroni-adjusted P value.

- significance: Significance label after Bonferroni correction.

- p_value_formatted: Formatted nominal P value.

- p_adj_bonferroni_formatted: Formatted Bonferroni-adjusted P value.

- test: Statistical test used.

- adjustment: Multiple-testing correction method.

Statistical analysis

Statistical significance was assessed by two-sided Welch's t-test followed by Bonferroni correction for multiple comparisons.

Significance labels are defined as follows:

- *: P < 0.05

- **: P < 0.01

- ***: P < 0.001

- ****: P < 0.0001

- n.s.: not significant

Exact adjusted P values

- WT versus Dux KO: adjusted P = 0.0273

- WT versus Ctbp1/2 DKO: adjusted P = 0.00640

- WT versus Ctbp1/2/Dux TKO: adjusted P = 3.29 x 10^-6

- Dux KO versus Ctbp1/2 DKO: adjusted P = 0.00745

- Dux KO versus Ctbp1/2/Dux TKO: adjusted P = 0.000193

- Ctbp1/2 DKO versus Ctbp1/2/Dux TKO: adjusted P = 0.00117

Notes

Only selected comparisons are annotated in the figure. Complete pairwise nominal P values and Bonferroni-adjusted P values are provided in WT_DuxKO_DKO_TKO_pairwise_ttest_Bonferroni.csv.
