# Supplementary material for: CtBP1/2 restrict DUX-dependent and independent 2C-like programs in mouse embryonic stem cells
Source: EMBO Rep. 2026 Aug 3;27(18):5317–40. doi: 10.1038/s44319-026-00881-7 (PMC13601577; doi:10.1038/s44319-026-00881-7)
Supplement: Supplementary file 17 [file 44319_2026_881_MOESM9_ESM.zip › Figure_2_source_data/Panel_K/README.docx]

**README**

**Panel K. Distribution of total divisions per MERVL-EGFP-positive founder.**

This source data file contains the raw values used to calculate the fraction of founders shown in Panel K. Each row represents one MERVL-EGFP-positive founder. Cell indicates the genotype group (WT, DKO, or TKO), and Splits indicates the cumulative total number of divisions produced by a single founder during tracking. Thus, a founder that divided once was assigned a value of 1, whereas a founder that reached the second generation was assigned a value of 3, reflecting one division in the first generation and two additional divisions in the second generation (1 + 2 = 3). Founders that reached the third generation or beyond were grouped as 4+. The fraction of founders in each category was then calculated within each genotype group.
