# Supplementary material for: CtBP1/2 restrict DUX-dependent and independent 2C-like programs in mouse embryonic stem cells
Source: EMBO Rep. 2026 Aug 3;27(18):5317–40. doi: 10.1038/s44319-026-00881-7 (PMC13601577; doi:10.1038/s44319-026-00881-7)
Supplement: Supplementary file 17 [file 44319_2026_881_MOESM9_ESM.zip › Figure_2_source_data/Panel_D_E/README.docx]

**README**

Source data and statistical analysis for Figure 2D,E

Files included

1. AP_number.csv

2. AP_number_TukeyHSD_all_comparisons.csv

Description

These files contain the numerical source data and statistical analysis for Figure 2E, which quantifies alkaline phosphatase (AP)-positive colony numbers in WT, Dux KO, Ctbp1/2 DKO and Ctbp1/2/Dux TKO ESCs. Figure 2D shows representative AP-staining images corresponding to the same experiment.

Figure 2D

Figure 2D shows representative images of AP-positive colonies. No statistical test was performed for Figure 2D because this panel shows representative images.

Figure 2E

Figure 2E shows quantification of AP-positive colony numbers.

AP_number.csv

This file contains the source values plotted in Figure 2E.

Columns

- Cell: Cell type or genotype analyzed.

- Number: Number of AP-positive colonies counted in each biological replicate.

Replicates

The analysis was performed using three biological replicates per genotype.

Plotting

Bars indicate the mean number of AP-positive colonies. Error bars indicate SEM. Individual data points represent biological replicates.

Summary statistics

- WT: n = 3, mean = 125.33, SEM = 48.76

- Dux KO: n = 3, mean = 90.33, SEM = 9.53

- Ctbp1/2 DKO: n = 3, mean = 36.67, SEM = 14.52

- Ctbp1/2/Dux TKO: n = 3, mean = 49.00, SEM = 11.50

AP_number_TukeyHSD_all_comparisons.csv

This file contains the statistical results for all pairwise comparisons among genotypes.

Columns

- comparison: Pairwise genotype comparison.

- difference: Difference in mean AP-positive colony number between the compared groups.

- lower_CI: Lower bound of the Tukey HSD confidence interval.

- upper_CI: Upper bound of the Tukey HSD confidence interval.

- p_adj_Tukey: Tukey HSD-adjusted P value.

- significance: Significance label after Tukey HSD correction.

- p_adj_Tukey_formatted: Formatted adjusted P value.

- test: Statistical test used.

Statistical analysis

Statistical significance was assessed by one-way ANOVA followed by Tukey's HSD multiple-comparison test.

Significance labels

- *: P < 0.05

- **: P < 0.01

- ***: P < 0.001

- ****: P < 0.0001

- n.s.: not significant

Exact adjusted P values

- WT versus Dux KO: adjusted P = 0.789 (n.s.)

- WT versus Ctbp1/2 DKO: adjusted P = 0.162 (n.s.)

- WT versus Ctbp1/2/Dux TKO: adjusted P = 0.252 (n.s.)

- Dux KO versus Ctbp1/2 DKO: adjusted P = 0.516 (n.s.)

- Dux KO versus Ctbp1/2/Dux TKO: adjusted P = 0.698 (n.s.)

- Ctbp1/2 DKO versus Ctbp1/2/Dux TKO: adjusted P = 0.987 (n.s.)

Notes

No significant differences were detected among the groups after Tukey HSD correction. Complete AP-positive colony counts and all adjusted P values are provided in the source data files listed above.
