# Supplementary material for: CtBP1/2 restrict DUX-dependent and independent 2C-like programs in mouse embryonic stem cells
Source: EMBO Rep. 2026 Aug 3;27(18):5317–40. doi: 10.1038/s44319-026-00881-7 (PMC13601577; doi:10.1038/s44319-026-00881-7)
Supplement: Supplementary file 17 [file 44319_2026_881_MOESM9_ESM.zip › Figure_2_source_data/Panel_I/README.docx]

**README**

**Source data and statistical analysis for Figure 2I**

**Files included**

**1. Cell_MaxIntensity.csv**

**2. Max_GFP_intensity_pvalues_Bonferroni.csv**

**Description**

**These files contain the numerical source data and statistical analysis used to generate the violin plot shown in Figure 2I. Figure 2I shows the maximal MERVL-EGFP fluorescence intensity per tracked cell in WT, Ctbp1/2 DKO and Ctbp1/2/Dux TKO ESCs.**

**Cell_MaxIntensity.csv**

**This file contains the source values plotted in Figure 2I.**

**Columns**

**- Cell: Cell type or genotype analyzed.**

**- Intensity: Maximal MERVL-EGFP fluorescence intensity per tracked cell.**

**To reduce the influence of transient fluctuations and single-frame intensity spikes, fluorescence values were smoothed using a sliding median of three consecutive frames. The maximum value of the smoothed fluorescence trace was used for plotting.**

**Tracked cells included**

**- WT: n = 76 tracked cells**

**- Ctbp1/2 DKO: n = 107 tracked cells**

**- Ctbp1/2/Dux TKO: n = 71 tracked cells**

**Max_GFP_intensity_pvalues_Bonferroni.csv**

**This file contains the statistical results for pairwise comparisons among genotypes.**

**Statistical analysis**

**Statistical significance was assessed using two-sided Wilcoxon rank-sum tests followed by Bonferroni correction for multiple comparisons.**

**Significance labels**

**- *: P < 0.05**

**- **: P < 0.01**

**- ***: P < 0.001**

**- ****: P < 0.0001**

**- n.s.: not significant**

**Exact adjusted P values**

**- WT versus Ctbp1/2 DKO: adjusted P = 1.65 × 10^-17 (****)**

**- WT versus Ctbp1/2/Dux TKO: adjusted P = 1.09 × 10^-18 (****)**

**- Ctbp1/2 DKO versus Ctbp1/2/Dux TKO: adjusted P = 0.07752 (n.s.)**

**Notes**

**Only selected comparisons are annotated in the figure. Complete nominal P values and Bonferroni-adjusted P values are provided in Max_GFP_intensity_pvalues_Bonferroni.csv.**
