# Supplementary material for: CtBP1/2 restrict DUX-dependent and independent 2C-like programs in mouse embryonic stem cells
Source: EMBO Rep. 2026 Aug 3;27(18):5317–40. doi: 10.1038/s44319-026-00881-7 (PMC13601577; doi:10.1038/s44319-026-00881-7)
Supplement: Supplementary file 17 [file 44319_2026_881_MOESM9_ESM.zip › Figure_2_source_data/Panel_G/README.docx]

**README**
This folder contains source videos for the Mervl-EGFP time-lapse imaging experiments in WT, *Ctbp1/2* DKO, and *Ctbp1/2/Dux* TKO ESCs.

**WT.avi**: Full-field time-lapse movie showing Mervl-EGFP reporter dynamics in WT cells.
**WT_Combined.avi**: Composite movie showing the tracking results together with the corresponding bright-field movie in WT cells.

**DKO.avi**: Full-field time-lapse movie showing Mervl-EGFP reporter dynamics in *Ctbp1/2* DKO cells.
**DKO_division.avi**: Representative composite movie showing a Mervl-EGFP-positive *Ctbp1/2 DKO* cell that undergoes cell division during live imaging.
**DKO_collapse.avi**: Representative composite movie showing a Mervl-EGFP-positive *Ctbp1/2 DKO* cell that collapses during live imaging.

**TKO.avi**: Full-field time-lapse movie showing Mervl-EGFP reporter dynamics in *Ctbp1/2/Dux* TKO cells.
**TKO_Combined.avi**: Composite movie showing the tracking results together with the corresponding bright-field movie in *Ctbp1/2/Dux* TKO cells.
