# Supplementary material for: CtBP1/2 restrict DUX-dependent and independent 2C-like programs in mouse embryonic stem cells
Source: EMBO Rep. 2026 Aug 3;27(18):5317–40. doi: 10.1038/s44319-026-00881-7 (PMC13601577; doi:10.1038/s44319-026-00881-7)
Supplement: Supplementary file 17 [file 44319_2026_881_MOESM9_ESM.zip › Figure_2_source_data/Panel_A/README.docx]

**README**

Source data for Figure 2A

Source data file

WT_DuxKO_DKO_TKO_FACS.pptx

Description

This source data file contains the flow cytometry plots and corresponding gate statistics used for Figure 2A. Figure 2A shows representative flow cytometry analysis of MERVL-EGFP reporter activity in wild-type (WT), Dux KO, Ctbp1/2 DKO and Ctbp1/2/Dux TKO ESCs.

The PowerPoint file contains exported flow cytometry plots for each genotype. The plots show MERVL-EGFP fluorescence and the corresponding gate used to define MERVL-EGFP-positive cells.

Samples included

WT

Dux KO

Ctbp1/2 DKO

Ctbp1/2/Dux TKO

Gate statistics

Gate statistics indicate the percentage of MERVL-EGFP-positive cells in each sample. The same gate setting was applied consistently across WT, Dux KO, Ctbp1/2 DKO and Ctbp1/2/Dux TKO ESC samples.

Notes

No statistical test was performed for Figure 2A because this panel shows representative flow cytometry plots. Quantification and statistical analysis of the MERVL-EGFP-positive population are shown in Figure 2B.
