# Supplementary material for: CtBP1/2 restrict DUX-dependent and independent 2C-like programs in mouse embryonic stem cells
Source: EMBO Rep. 2026 Aug 3;27(18):5317–40. doi: 10.1038/s44319-026-00881-7 (PMC13601577; doi:10.1038/s44319-026-00881-7)
Supplement: Supplementary file 17 [file 44319_2026_881_MOESM9_ESM.zip › Figure_2_source_data/Panel_F/README.docx]

**README**

Source data for Figure 2F

File included

Sorted_FACS_data.pptx

Description

This source data file contains the flow cytometry plots and corresponding gate statistics used for Figure 2F. Figure 2F shows representative flow cytometry analysis of sorted MERVL-EGFP-positive cells from Ctbp1/2 DKO and Ctbp1/2/Dux TKO ESCs immediately after sorting and after 8 days of re-culture.

Sorted_FACS_data.pptx

This PowerPoint file contains the exported flow cytometry plots for the following samples:

- Ctbp1/2 DKO, day 0

- Ctbp1/2 DKO, day 8

- Ctbp1/2/Dux TKO, day 0

- Ctbp1/2/Dux TKO, day 8

Gate statistics

Gate statistics indicate the percentage of MERVL-EGFP-positive cells in each sample. The same gate setting was applied consistently to the day 0 and day 8 samples within each genotype.

Notes

No statistical test was performed for Figure 2F because this panel shows representative flow cytometry plots. The corresponding gate statistics are provwided in the source data file.
