# Supplementary material for: CtBP1/2 restrict DUX-dependent and independent 2C-like programs in mouse embryonic stem cells
Source: EMBO Rep. 2026 Aug 3;27(18):5317–40. doi: 10.1038/s44319-026-00881-7 (PMC13601577; doi:10.1038/s44319-026-00881-7)
Supplement: Supplementary file 17 [file 44319_2026_881_MOESM9_ESM.zip › Figure_2_source_data/Panel_H/README.docx]

**README**

**Source data for Figure 2H**

**File included**

**Fluorescence_timecourse.csv**

**Description**

**This file contains the source fluorescence time-course data used to generate the heatmap shown in Figure 2H. Figure 2H shows GFP fluorescence intensity traces of individual tracked MERVL-EGFP-positive cells from WT, Ctbp1/2 DKO and Ctbp1/2/Dux TKO ESCs, aligned to the onset of detectable MERVL-EGFP signal.**

**Fluorescence_timecourse.csv**

**Each row corresponds to one time-point measurement from one tracked cell or lineage.**

**Columns**

**- CellType: Cell type or genotype analyzed.**

**- TrackID: Identifier for each tracked cell or lineage.**

**- Time_min: Time in minutes from the onset of detectable MERVL-EGFP signal.**

**- Fluorescence_raw: Raw GFP fluorescence intensity value measured at the indicated time point.**

**- Fluorescence_med3: GFP fluorescence intensity after 3-point median smoothing. This value was used for heatmap visualization.**

**Tracked cells/lineages included in this file**

**- WT: 50 tracked cells/lineages; time range, 0 to 4020 min from GFP onset**

**- Ctbp1/2 DKO: 90 tracked cells/lineages; time range, 0 to 4290 min from GFP onset**

**- Ctbp1/2/Dux TKO: 62 tracked cells/lineages; time range, 0 to 4290 min from GFP onset**

**Plotting**

**Heatmaps were generated from the fluorescence time-course values aligned to GFP onset. Tracked cells/lineages were ordered within each genotype using the same order as in the plotted heatmap.**

**Statistical analysis**

**No statistical test was performed for Figure 2H because this panel visualizes fluorescence trajectories as a heatmap. Quantitative statistical analyses of the time-lapse imaging data are shown in Figure 2I and Figure 2J, and division counts are summarized in Figure 2K.**

**Notes**

**The number of tracked cells/lineages shown in Figure 2H should match the number of unique TrackID values for each genotype in this source data file.**
